# Identification of novel pyrrolopyrimidine and pyrrolopyridine derivatives as potent ENPP1 inhibitors

**DOI:** 10.1080/14756366.2022.2119566

**Published:** 2022-09-07

**Authors:** Hee Jin Jeong, Hye Lim Lee, Sung Joon Kim, Jeong Hyun Jeong, Su Hyun Ji, Han Byeol Kim, Miso Kang, Hwan Won Chung, Chan Sun Park, Hyunah Choo, Hyo Jae Yoon, Nam-Jung Kim, Duck-Hyung Lee, Sang Hee Lee, Seo-Jung Han

**Affiliations:** a Chemical & Biological Integrative Research Center, Korea Institute of Science and Technology, Seoul, Republic of Korea; b Department of Chemistry, Korea University, Seoul, Republic of Korea; c Brain Science Institute, Korea Institute of Science and Technology, Seoul, Republic of Korea; d Department of Basic Pharmaceutical Science, College of Pharmacy, Kyung Hee University, Seoul, Republic of Korea; e TXINNO Bioscience Inc, Yongin-si, Gyeonggi-do, Republic of Korea; f Department of Chemistry, Sogang University, Seoul, Republic of Korea; g Computational Science Research Center, Korea Institute of Science and Technology, Seoul, Republic of Korea; h Department of Life and Nanopharmaceutical Sciences, Graduate School, Kyung Hee University, Seoul, Republic of Korea; i Department for HY-KIST Bio-convergence, Hanyang University, Seoul, Republic of Korea; j Division of Bio-Medical Science & Technology, KIST School, University of Science and Technology, Seoul, Republic of Korea

**Keywords:** ENPP1, STING, innate immunity, cancer immunotherapy

## Abstract

In an effort to discover novel scaffolds of non-nucleotide-derived Ectonucleotide pyrophosphatase/phosphodiesterase 1 (ENPP1) inhibitors to stimulate the Stimulator of Interferon Genes (STING) pathway, we designed and synthesised pyrrolopyrimidine and pyrrolopyridine derivatives and performed structure-activity relationship (SAR) study. We found **18p** possessed high potency (IC_50_ = 25.0 nM) against ENPP1, and activated STING pathway in a concentration dependent manner. Also, in response to STING pathway activation, cytokines such as IFN-*β* and IP-10 were induced by **18p** in a concentration dependent manner. Finally, we discovered that **18p** causes inhibition of tumour growth in 4T1 syngeneic mouse model. This study provides new insight into the designing of novel ENPP1 inhibitors and warrants further development of small molecule immune modulators for cancer immunotherapy.

## 1. Introduction

Cancer immunotherapy has recently emerged as a new paradigm in treating cancer patients in that patient’s immune system components are involved in cancer therapies^1^. Although several adaptive immune checkpoint inhibitors such as anti-PD-1, anti-PD-L1, and anti-CTLA-4 have received FDA approval, limitations of the immune checkpoint inhibitors have been reported including low response rate in many cases^2^. The reason of initial resistance to immune checkpoint inhibitors is that most tumours lack sufficient T cell infiltration (i.e. “cold” tumour). Thus, activating innate immune system, which is upstream of adaptive immune tumour-infiltrating lymphocytes (TILs), to turn “cold” tumour into “hot” tumour could be a novel strategy in cancer immunotherapy^3^.

The innate immune system is the first responder against microbial infection. When the foreign DNAs from pathogens or dead cells are detected by cGAS (cyclic GMP-AMP synthase), cGAS-STING pathway is activated^4^. Activated cGAS by DNA binding synthesises cGAMP (cyclic GMP-AMP), which activates STING protein (stimulator of interferon genes) on the endoplasmic reticulum. Activated STING then transmit a signal that leads to production of interferons (IFNs) and ultimately recruits tumour-infiltrated lymphocytes (TIL) around tumour. Ectonucleotide pyrophosphatase/phosphodiesterase 1 (ENPP1) has been discovered as the principal hydrolase for cGAMP and thus, ENPP1 restrains innate immune responses to cancer. It has been reported that both genetic knockdown and inhibition of ENPP1 by small molecule could increase tumour-infiltrating DCs and decrease tumour growth^5^.

**Figure 1.**
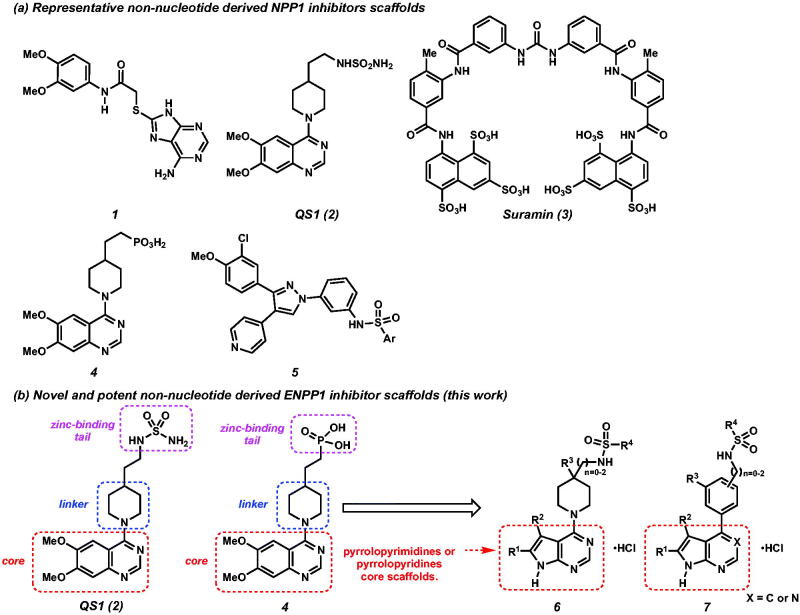
(a) Representative ENPP1 inhibitors. (b) Design of novel ENPP1 inhibitors possessing pyrrolopyrimidines and pyrrolopyridines core scaffolds.

Although ENPP1 have recently drawn much attention in the fields of medicinal chemistry^5^^,^^6^ and efforts to synthesise small molecule ENPP1 inhibitor have been reported, development of novel and potent drug-like ENPP1 inhibitor has proven challenging (Figure 1a). Therefore, discovery of a novel scaffold for synthesis of non-nucleotide derived ENPP1 inhibitor is highly important. Inspired by the reported ENPP1 inhibitors, QS1(**2**)^6^^h,i^ and **4**^5^^,^^6^^a^, we designed and synthesised a series of new sulfamide derivatives possessing the pyrrolopyrimidine and pyrrolopyridine core scaffolds and performed SAR study in an effort to discover novel and potent ENPP1 inhibitors (Figure 1b).

## 2. Results and discussion

### 2.1. Chemistry

#### 2.1.1. Synthesis of pyrrolopyrimidine and pyrrolopyridine derivatives 18, 20, and 25

**Scheme 1.**
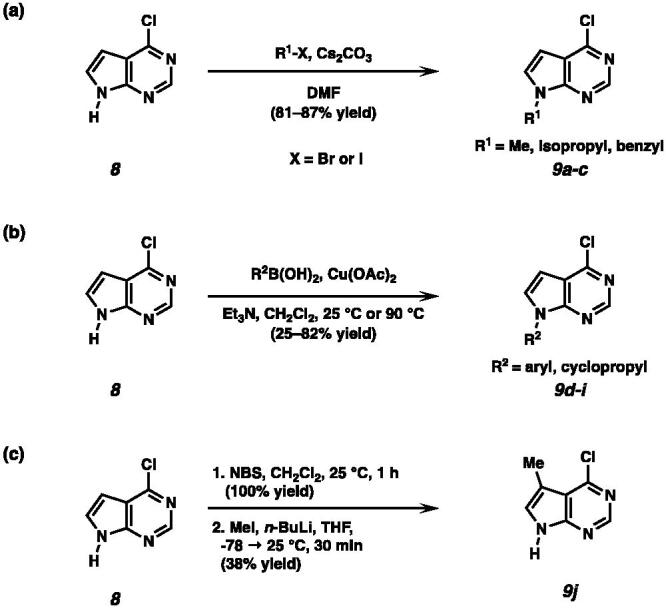
(a) Synthesis of *N*-alkylated pyrrolopyrimidines under basic conditions. (b) Synthesis of *N*-aryl or *N*-cyclopropyl substituted pyrrolopyrimidines by Chan-Lam coupling. (c) Synthesis of 4-chloro-5-methyl pyrrolopyrimidine **9j**.

Alkylation of 6-chloro-7-deazapurine **8** with methyl, isopropyl, and benzyl halides provided *N*-alkylated pyrrolopyrimidines in good yields (Scheme 1a)^7^. Aryl and cyclopropyl substituted pyrrolopyrimidines were synthesised by Chan-Lam coupling reaction (Scheme 1b)^8^. To investigate the effect of the methyl group at C(5) of the pyrrolopyrimidine core on ENPP1 inhibitory activities, 4-chloro-5-methyl pyrrolopyrimidine **9j** was synthesised by bromination of 6-chloro-7-deazapurine **8**, subsequent lithium-halogen exchange and quenching with iodomethane (Scheme 1c)^9^.

**Scheme 2.**
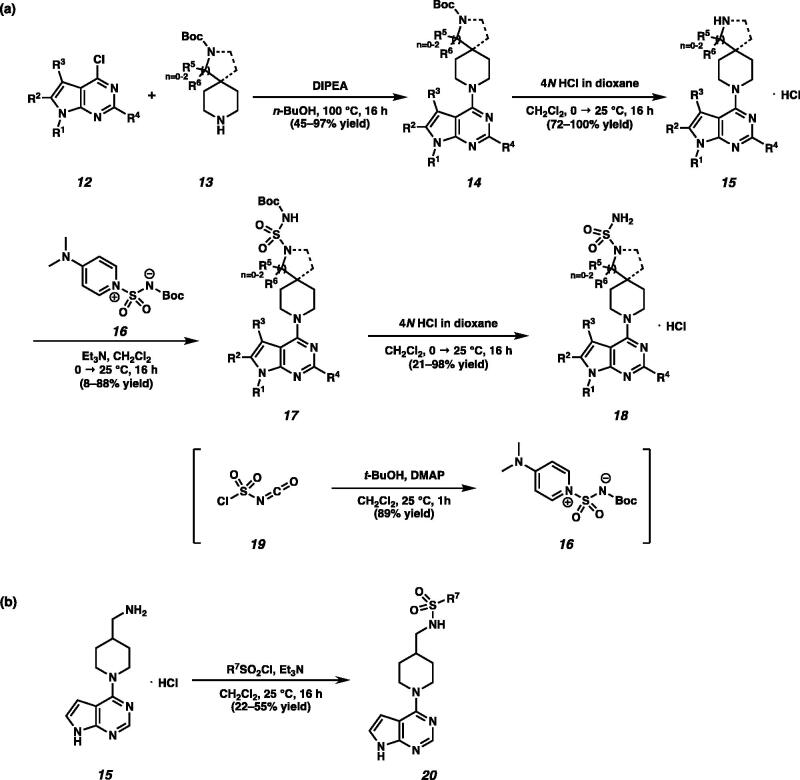
(a) Synthesis of piperidine substituted sulfamides **18**. (b) Synthesis of sulfone amides **20**.

Nucleophilic aromatic substitution of pyrrolopyrimidines or pyrrolopyridines **12** with piperidines **13** afforded carbamates **14** in 45–97% yields^10^. Removal of the Boc group under acidic conditions provided amines **15** in good yields. Reaction between amines **15** and Burgess-type reagent **16**, which was developed by Winum et al. provided **17**^11^. Cleavage of the Boc group in compounds **17** with 4 *N* HCl in dioxane formed sulfamides **18** (Scheme 2a). Sulfone amides **20** were also synthesised by *N*-sulfonylation of amines **15** (Scheme 2b)^12^.

**Scheme 3.**
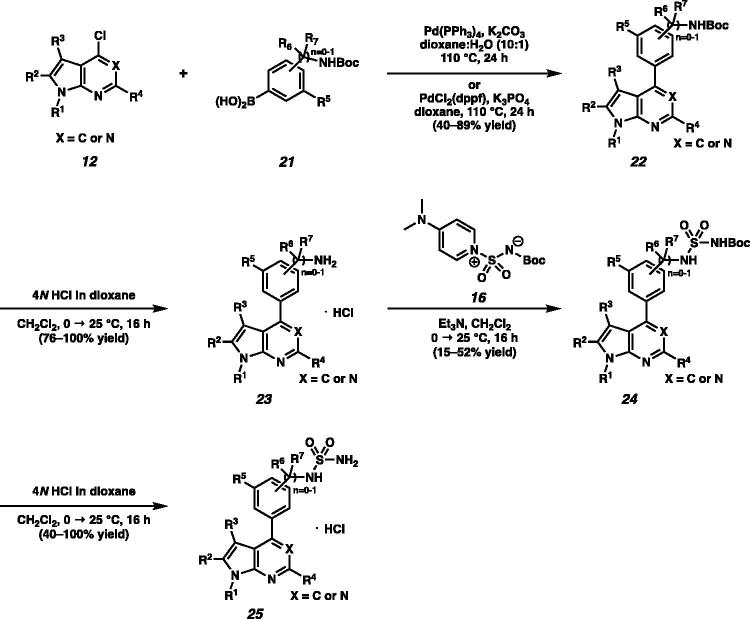
Synthesis of phenyl substituted sulfamide **25**.

In addition, we attempted to prepare 4-phenyl substituted pyrrolopyrimidines or pyrrolopyridines **25** (Scheme 3). The Suzuki coupling of 4-chloropyrrolopyrimidines or 4-chloropyrrolopyridines **12** and boronic acids **21** generated Boc-protected amines **22** in moderate to good yields (Scheme 3).^10a^ Then, cleavage of the Boc group under acidic conditions and subsequent addition of sulfamide moieties to the free amines **23** followed by the removal of the Boc group afforded **25**.

### 2.2. Structure-activity relationships

With the compounds in hand, % inhibition and IC_50_ values for all synthetic 4-piperidine and 4-aryl substituted pyrrolopyridines or pyrrolopyrimidines (i.e. **18**, **20**, and **25**) were determined using *in vitro* recombinant human ENPP1 enzymatic assay with AMP-Glo assay (Table 1)^13^. Since MV658, discovered by Mavupharma, was reported to possess less than 100 nM K_i_ value, We used MV658 as a positive control in enzymatic assay (Table 1, entry 1)^10a^. Compound **18a**, which possessed the ethylene group between the piperidine and the sulfamide showed IC_50_ = 1890 nM against ENPP1 (Table 1, entry 2). Addition of methyl sulphide at C(2) position of pyrrolopyrimidine resulted in lower activity (IC_50_ = 21610 nM; Table 1, entry 3). Interestingly, shorter linker between the piperidine and the sulfamide resulted in remarkable potency (IC_50_ = 19.0 nM; entry 4). Although *N*-methyl pyrrolopyrimidine possessed high potency (IC_50_ = 51.4 nM; entry 5), isopropyl and cyclopropyl substituted compounds, **18e** and **18f** showed diminished activities (IC_50_ = 20660 nM and 74.0 nM, respectively; entries 6 and 7). The *N*-phenyl substituted pyrrolopyrimidine also possessed moderate potency (IC_50_ = 298 nM; entry 8). However, 4-fluorophenyl and 4-methoxyphenyl substituted pyrrolopyrimidines exhibited lower ENPP1 percent inhibitory activities (entries 9 and 10). Also, benzyl substituted pyrrolopyrimidine possessed only 17% ENPP1 inhibitory activity (entry 11). IC_50_ values of heterocycle substituted pyrrolopyrimidines were also determined (entries 12 and 13). Furan substituted pyrrolopyrimidine **18k** resulted in high potency (IC_50_ = 54.1 nM; entry 12). However, *N*-pyridine substituted pyrrolopyrimidine **18l** showed diminished potency (IC_50_ = 1853 nM; entry 13). Addition of the methyl substituent at C(5) and C(6) positions negatively impact on the ENPP1 inhibitory activities (entries 14 and 15). Addition of the methyl group at alkyl linker resulted in lower activity (entry 16). Interestingly, piperidine **18p**, possessing a quaternary centre showed highly enhanced ENPP1 inhibitory activity (IC_50_ = 25.0 nM; entry 17). Compound **18q**, which possessed no alkyl linkers between the sulfamide and the piperidine exhibited lower activities (IC_50_ = 3323 nM; entry 18). Spiropiperidine **18r** also exhibited high potency (IC_50_ = 60.5 nM; entry 19). Introduction of the sulfone amide functional groups instead of the sulfamide exhibited lower activities (entries 20–22). Although **18c** showed similar ENPP1 inhibitory activity compared to **18p**, we decided to evaluate cell-based assay with **18p**, which possessed larger cLogP values.

**Table 1.** Enzymatic inhibitory activities of 4-piperidine substituted sulfamides **18** and sulfone amides **20** against ENPP1.

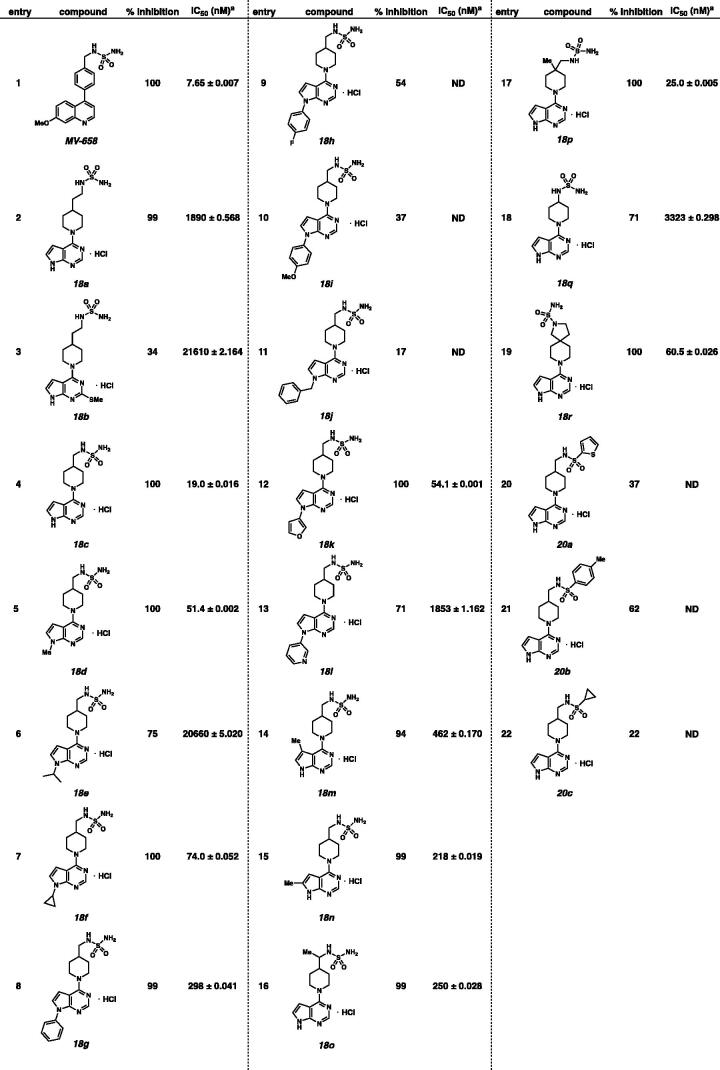

^a^Average ± *SD* from two or three independent repeats.

Moreover, percent inhibition and IC_50_ values against ENPP1 with 4-phenyl substituted sulfamides **25** were evaluated (Table 2). The percent inhibitory activities of 4-phenyl substituted sulfamides **25** was quite low compared to those with 4-piperidine substituted sulfamides **18**. Both **25a** and **25b** displayed low percent inhibitory activities (entries 2 and 3). *N*-benzyl sulfamides **25c** and **25d** were found to have weak potency to ENPP1 (entries 4 and 5). A similar inhibitory potency was observed with *N*-methylated pyrrolopyrimidine **25e** (entry 6). Pyrrolopyridine **25f** was more potent than pyrrolopyrimidine **25d** (entries 5 and 7). Addition of the sulfamide functional group at the meta position of phenyl has no beneficial effect on potency (entry 8). Interestingly, compound **25h**, containing gen-dimethyl group exhibited enhanced potency (entry 9). However, *N*-pyridyl compound **25i** showed no activity (entry 10). Surprisingly, addition of mono-methyl substitution at the benzylic position resulted in remarkable potency against ENPP1 (**25j**, IC_50_ = 28.3 nM, entry 11).

**Table 2.** Enzymatic inhibitory activities of sulfamides **25** against ENPP1.

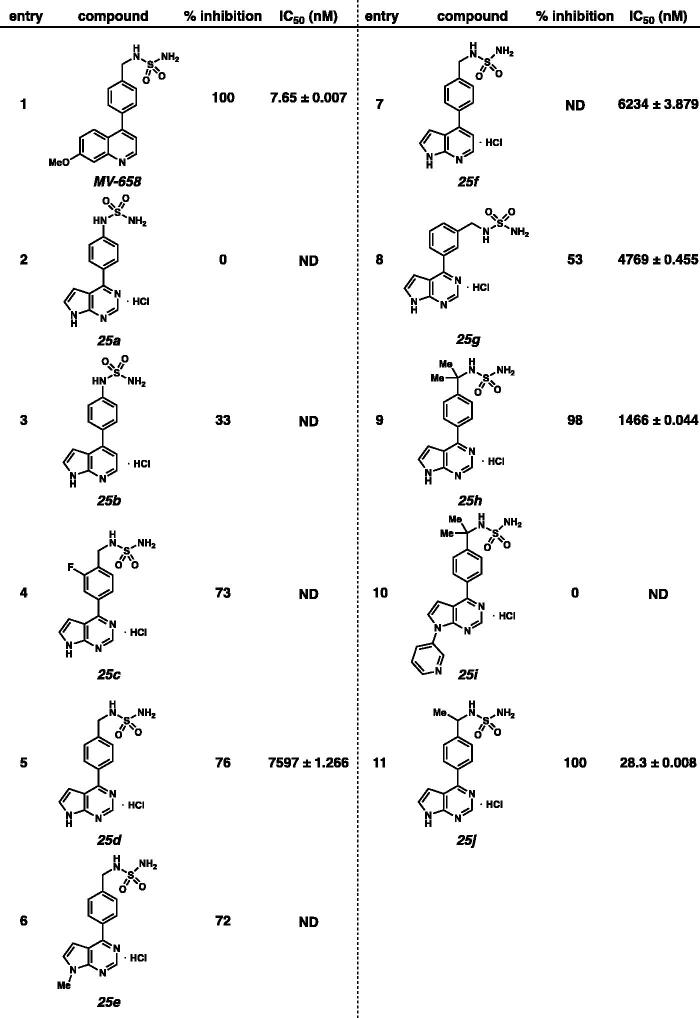

^a^Average ± *SD* from two or three independent repeats.

### 2.3. Docking studies

**Figure 2.**
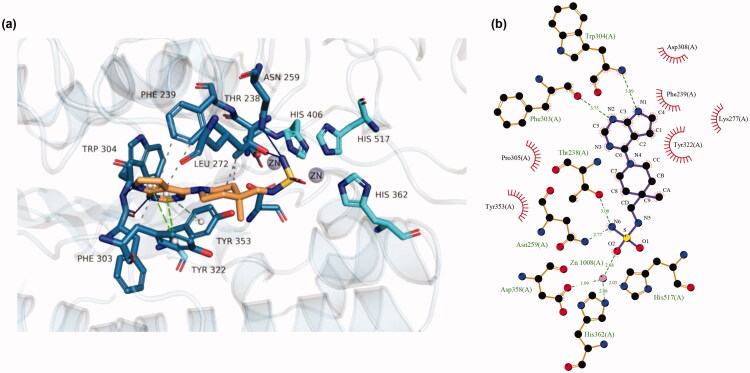
(a) Docking model of **18p** bound to ENPP1. **18p** (light gold) is represented in stick model and other protein domains are briefly shown in cartoon model. Two zinc (gray) and their coordinating Histidines (cyan) are shown. Several key residues interacting with **18p** are shown in blue. (b) The ligand (purple) and protein (brown) were represented in ball (C: black; N: blue; O: red; Zn: cyan) and stick (bond) model, green dash shows hydrogen bonding or metal coordination. Hydrophobic intermolecular interactions are described by half whisker shapes with its amino acid residues.

The three-dimensional structures of ENPP1 in complex with **18p** were predicted by protein-ligand docking study. Compound **18p** occupies the known cGAMP binding site of ENPP1. The pyrrololpyrimidine core forms π-π interactions with Y322 and Y353. In addition, nitrogens on the pyrrolopyrimidine forms hydrogen bond interactions with W304 and F303. The sulfamide functional group interacts with the zinc ion and the NH_2_ of the sulfamide forms hydrogen bonds with T238 and N259. The piperidine linker forms hydrophobic interactions with L272 (Figure 2).

### 2.4. Inhibition of CYPs, microsomal stabilities, and PK profiling

Encouraged by the great inhibitory activities of **18p** against ENPP1, we evaluated the inhibitory activities of **18p** against cytochrome P450s (CYPs) (Table 3). We observed that five cytochrome P450 enzymes, CYP1A2, CYP2C9, CYP2C19, CYP2D6 and CYP3A4, were only slightly inhibited by **18p**. We also found that **18p** possesses good microsomal stabilities (>60%) against human, rat, and mouse liver microsomes (Table 4). Moreover, the mouse pharmacokinetic profile of compound **18p** showed great oral bioavailability, moderately long half-life, and a plasma exposure of 1698 ng•h/mL following an oral dose of 10 mg/kg (Table 5).

**Table 3.** CYP inhibition profile.

compound (10 μM)	% inhibition				
	CYP1A2	CYP2C9	CYP2C19	CYP2D6	CYP3A4
18p	12.0	0.0	10.3	0.5	8.9

**Table 4.** Human, rat, and mouse liver microsomal stability of **18p** (% remaining during 30 min at 1 µM).

	Microsomal stability (%)		
	Human	Rat	Mouse
18p	93	96	83

**Table 5.** Pharmacokinetic properties of **18p** in BALB/c mice.

Route	Dose (mg/kg)	*T*_1/2_ (h)	*C*_max_ (ng/mL)	*T*_max_ (h)	AUC_0-t_ (ng·h/mL)	AUC (ng·h/mL)	CI (L/h/kg)	*V*_ss_(L/kg)	*F*(%)
PO	10	1.56	1554	0.5	1694	1698			>100
IV	1	0.25	303	0.083	125	126	7.5	2.3	

### 2.5. Biological evaluation

**Figure 3.**
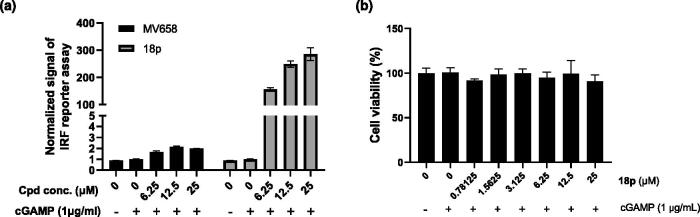
(a) Luciferase assay for measuring ISRE-mediated reporter gene induction of **18p** in THP-1 cells. THP-1 cells were stimulated by the indicated concentration of **18p** for 24 h. Y-axis indicates the normalized luciferase signal by DMSO control. (b) Cell viability of **18p** in THP-1 cells.

To verify the cellular activity of **18p** with cGAMP, we tested whether the compound could stimulate type I IFN response using THP-1 human monocyte cells bearing interferon sensitive response elements (ISRE) luciferase reporter system because THP-1 is well-known monocyte cell line and widely used to evaluate cGAS-STING mediated innate immune activity. We used MV658 as a positive control for the cell-based assay^10a^. While the low concentration of cGAMP (1 μg/mL) only induce luciferase signal slightly, co-treatment of **18p** clearly increased the cGAMP-induced ISRE activation in dose-dependent manner (Figure 3a). Treatment of **18p** dramatically increased ISRE signal compared to the result with MV658. Furthermore, no obvious toxicity was observed in **18p**-treatment THP-1 cells in all concentrations tested (Figure 3b).

**Figure 4.**
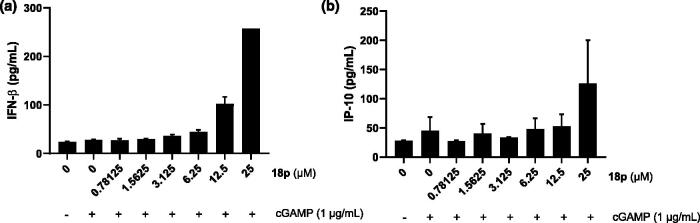
(a) Determination of IFN-*β* cytokine release by ELISA. THP-1 cells were stimulated by the indicated concentration of **18p** for 8 h. (b) Determination of IP-10 cytokine secretion by ELISA. THP-1 cells were stimulated by the indicated concentration of **18p** for 8 h.

To elucidate the endogenous immune activation by **18p**, we investigated the secretion of innate immune-related cytokines such as IFN-*β* and IP-10 which have been mainly considered as biomarkers for STING activation. Extracellular secretion of IFN-*β* and IP-10 was measured by enzyme-linked immunosorbent assay (ELISA). We discovered that co-treatment of **18p** with cGAMP secreted more cytokine than with cGAMP alone in both IFN-*β* and IP-10 analysis (Figure 4).

**Figure 5.**
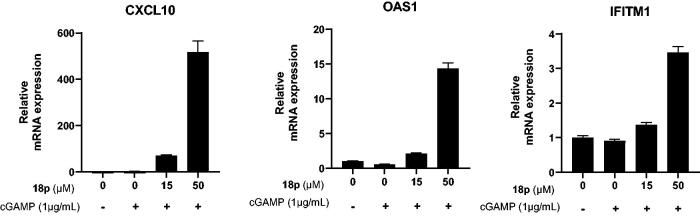
Real-time PCR analysis for gene expression of various interferon stimulated genes (ISG), CXCL10, OAS1, and IFITM1. THP-1 cells were stimulated by indicated concentration of **18p** for 4 h. The relative gene expression level was normalized by GAPDH as an internal standard. Graphs depict mean and RQ max values.

Next, we further explored type I IFN response induced by activation of STING signalling. The gene expression for various interferon stimulated genes (ISGs) such as CXCL10, OAS1, and IFITM1 was analysed by real-time PCR. Interestingly, co-treatment of **18p** with cGAMP induced expression of all ISG genes we tested, and triggered transcription of the key activation markers like CXCL10. The result was corresponded to ELISA result shown in Figure 5.

**Figure 6.**
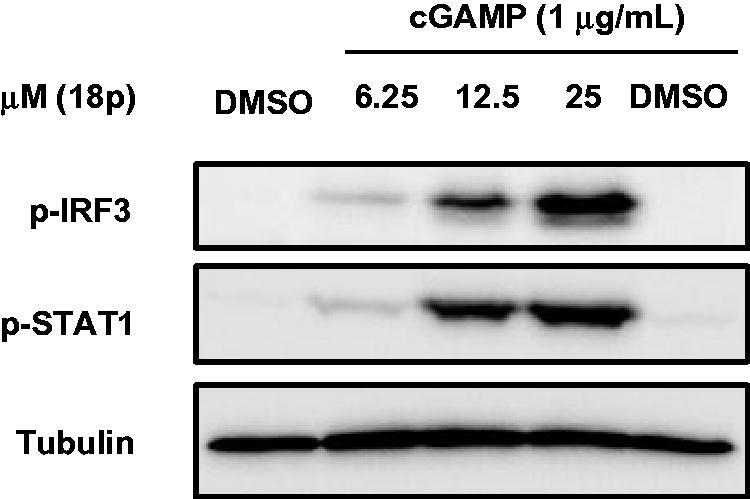
Western blot analysis for STING downstream signalling pathway. THP-1 cells were treated with cGAMP (1 μg/mL) first, and then treated with either DMSO or **18p** at indicated concentration for 6 h. Tubulin was used as a loading control.

To evaluate whether **18p** induced desired immune response via activation of STING pathway, we investigated the activation of downstream effector proteins, IRF3 and STAT1 that were known as major regulatory proteins of STING and IFN-*β*, respectively. By western blot analysis, the increased phosphorylation of IRF3 and STAT1 was observed in dose-dependent manner by co-treatment of cGAMP and **18p** in THP-1 cells (Figure 6). Based on all these results, we concluded that potent ENPP1 inhibitor **18p** obviously stimulated cGAMP-mediated STING activation by resulting in the synergy effect with cGAMP for innate immunity.

### 2.6. *In vivo* efficacy

**Figure 7.**
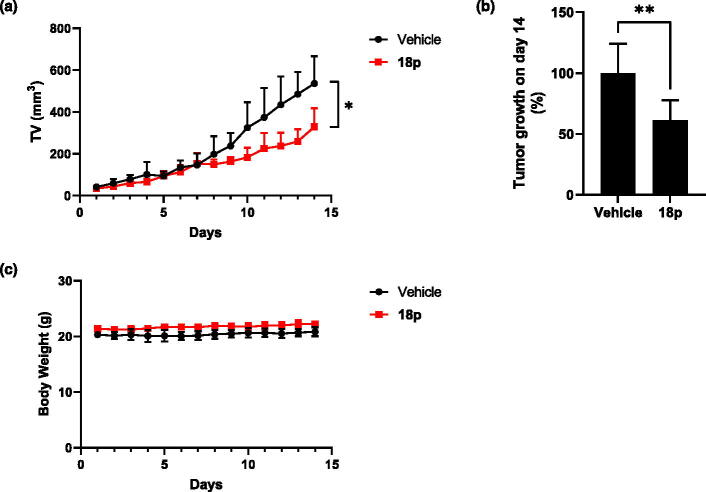
4 × 10^5^ of 4T1 cells were subcutaneously inoculated into right side flank of BLAB/c mice (*n* = 6). Mice were orally administrated by **18p** (40 mg/kg) or vehicle by every day. (a) Tumour growth curve of **18p** or vehicle. X-axis indicates days after treatment. (b) Tumour growth (%) on day 14. (c) Body weight of **18p** or vehicle. X-axis indicates days after treatment. Statistics for tumour volume and tumour growth were analysed by 2way ANOVA and student *t*-test. *p < 0.05. **p < 0.01. Graphs are depicted by mean and SD.

The Bakhoum group showed that *Enpp1*-KO 4T1 mouse model led to significant longer overall survival rate^14^. Thus, we evaluated the anti-cancer efficacy in the 4T1 syngeneic mouse tumour model to test the therapeutic potential of **18p**. We postulated the marginal amount of cGAMP released by tumour could initiate the synergy effect of **18p**, then administrated the compound by single-treatment^5^. After 4T1 tumour cells were implanted and grown in the flank of mice, **18p** was orally administered (40 mg/kg) once daily and monitored the tumour volume and body weight of each mouse. After two weeks of treatment, **18p** significantly suppressed tumour growth (TGI = 39% on day 14, Figure 7(a,b) and no weight loss occurred upon treatment of **18p** (Figure 7c).

## 3. Conclusion

In conclusion, we discovered the novel small molecular ENPP1 inhibitor, **18p** possessing the pyrrolopyrimidine core. Pyrrolopyrimidine **18p** showed high *in vitro* potency (IC_50_ = 25.0 nM) against ENPP1. Also, **18p** increased the cGAMP-induced ISRE activation and induced cytokine secretion in a concentration dependent manner in THP-1 dual cells. These results showed that **18p** obviously stimulated cGAMP-mediated STING activation. Finally, we discovered that **18p** causes suppression of the tumour growth in 4T1 syngeneic mouse model. This study provides insightful guidelines for design and development of novel ENPP1 inhibitors.

## 4. Experimental

### 4.1. Chemistry

#### 4.1.1. General informations

Unless otherwise stated, reactions were performed in flame-dried glassware under a nitrogen atmosphere using dry solvents. Reaction progress was monitored by thin-layer chromatography (TLC). Purified water was obtained using a Barnstead NANOpure Infinity UV/UF system. Brine solutions are saturated aqueous solutions of sodium chloride. Commercially available reagents were purchased from Sigma-Aldrich, Acros Organics, Combi-Blocks, TCI or Alfa Aesar, and BLDpharm used as received unless otherwise stated. Reaction temperatures were controlled by an IKAmag temperature modulator unless otherwise indicated. TLC was performed using E. Merck silica gel 60 F254 precoated glass plates (0.25 mm) and visualised by UV fluorescence quenching, KMnO_4_ staining. Silicycle SiliaFlash P60 Academic Silica gel (particle size 0.040–0.064 mm) was used for flash column chromatography. ^1^H NMR spectra were recorded on Bruker 400 MHz, 600 MHz, and 800 MHz spectrometer and are reported relative to residual CDCl_3_ (δ 7.26 ppm), CD_3_OD (δ 3.31 ppm), D_2_O (δ 4.79 ppm) or (CD_3_)_2_SO (δ 2.50 ppm). ^13^C NMR spectra are recorded on Bruker 400 MHz, 600 MHz, and 800 MHz spectrometer (101 MHz & 151 MHz & 201 MHz) and are reported relative to CD_3_OD (δ 49.00 ppm) or (CD_3_)_2_SO (δ 39.52 ppm). ^19 ^F NMR spectra are recorded on Bruker 400 MHz spectrometer (377 MHz). Data for ^1^H NMR are reported as follows: s = singlet, d = doublet, t = triplet, q = quartet, p = pentet, sept = septuplet, m = multiplet, br s = broad singlet, br d = broad doublet, app = apparent. Data for ^13^C are reported in terms of chemical shifts (δ ppm). High resolution mass spectra (HRMS) were obtained from Bruker ESI Q-TOF Mass Spectrometer with electrospray ionisation (ESI+), atmospheric pressure chemical ionisation (APCI+), or mixed ionisation mode (MM: ESI-APCI+). HPLC was performed on an Agilent 1100 system with an Agilent EC-C18 column (4.6 × 150 mm, 4 µm).

#### 4.1.2. 4-Chloro-7-methyl-7H-pyrrolo[2,3-d]pyrimidine (9a)

To a stirred solution of pyrrolopyrimidine **8** (250 mg, 1.63 mmol, 1.00 equiv) and Cs_2_CO_3_ (800 mg, 2.45 mmol, 1.50 equiv) in DMF (5.40 mL) was added MeI (0.200 mL, 3.26 mmol, 2.00 equiv). The reaction mixture was stirred for 1 h at 25 °C. The solution was filtered through celite and H_2_O was added. The aqueous phase was extracted with EtOAc (3 × 7.00 mL). The combined organic phase was washed with brine, dried over MgSO_4_ and concentrated *in vacuo*. The residue was purified by flash column chromatography on silica gel (EtOAc:hexanes = 1:4) to give methylated pyrrolopyrimidine **2** (224 mg, 82% yield). ^1^H NMR (400 MHz, CDCl_3_) δ 8.65 (s, 1H), 7.22 (d, *J* = 3.5 Hz, 1H), 6.61 (d, *J* = 3.5 Hz, 1H), 3.90 (s, 3H).

#### 4.1.3. 4-Chloro-7-isopropyl-7H-pyrrolo[2,3-d]pyrimidine (9b)

A solution of pyrrolopyrimidine **8** (90.0 mg, 0.586 mmol, 1.00 equiv) and Cs_2_CO_3_ (290 mg, 0.879 mmol, 1.50 equiv) in DMF (1.00 mL) was stirred for 30 min at 25 °C. Then, 2-bromopropane was added and the solution was stirred for 5 h at 70 °C. The solution was filtered through celite and H_2_O was added. The aqueous phase was extracted with EtOAc (3 × 1.50 mL). The combined organic phase was washed with brine, dried over MgSO_4_ and concentrated *in vacuo*. The residue was purified by flash column chromatography on silica gel (EtOAc:hexanes = 1:4) to give pyrrolopyrimidine **9b** (93.0 mg, 81% yield). ^1^H NMR (400 MHz, MeOD) δ 8.55 (s, 1H), 7.70 (d, *J* = 3.7 Hz, 1H), 6.67 (d, *J* = 3.7 Hz, 1H), 5.12 (p, *J* = 6.8 Hz, 1H), 1.55 (d, *J* = 6.8 Hz, 6H).

#### 4.1.4. 7-Benzyl-4-chloro-7H-pyrrolo[2,3-d]pyrimidine (9c)

A solution of pyrrolopyrimidine **8** (200 mg, 1.30 mmol, 1.00 equiv) and Cs_2_CO_3_ (640 mg, 1.95 mmol, 1.50 equiv) in DMF (2.17 mL) was stirred for 30 min at 25 °C. Then, benzyl bromide (232 µL, 1.95 mmol, 1.50 equiv) was added and the solution was stirred for 5 h at 70 °C. The solution was filtered through celite and H_2_O was added. The aqueous phase was extracted with EtOAc (3 × 3.00 mL). The combined organic phase was washed with brine, dried over MgSO_4_ and concentrated *in vacuo*. The residue was purified by flash column chromatography on silica gel (EtOAc:hexanes = 1:4) to give *N*-benzyl pyrrolopyrimidine **9c** (276 mg, 87% yield). ^1^H NMR (400 MHz, MeOD) δ 8.60 (s, 1H), 7.57 (d, *J* = 3.7 Hz, 1H), 7.35 − 7.23 (m, 5H), 6.69 (d, *J* = 3.6 Hz, 1H), 5.52 (s, 2H).

#### 4.1.5. 4-Chloro-7-phenyl-7H-pyrrolo[2,3-d]pyrimidine (9d)

To a stirred solution of pyrrolopyrimidine **8** (90.0 mg, 0.586 mmol, 1.00 equiv) in CH_2_Cl_2_ (2.00 mL) were added phenylboronic acid (79.0 mg, 0.645 mmol, 1.10 equiv), Cu(OAc)_2_ (213 mg, 1.17 mmol, 2.00 equiv), and Et_3_N (0.820 mL, 5.86 mmol, 10.0 equiv). The solution was stirred 12 h at 90 °C. The solution was filtered through celite and H_2_O was added. The aqueous phase was extracted with CH_2_Cl_2_ (3 × 2.50 mL). The combined organic phase was washed with brine, dried over MgSO_4_ and concentrated *in vacuo*. The residue was purified by flash column chromatography on silica gel (EtOAc:hexanes = 1:6) to give *N*-phenyl pyrrolopyrimidine **9d** (107 mg, 80% yield). ^1^H NMR (400 MHz, CDCl_3_) δ 8.71 (s, 1H), 7.71 − 7.67 (m, 2H), 7.60 − 7.52 (m, 3H), 7.46 − 7.39 (m, 1H), 6.80 (d, *J* = 3.7 Hz, 1H).

#### 4.1.6. 4-Chloro-7–(4-fluorophenyl)-7H-pyrrolo[2,3-d]pyrimidine (9e)

To a stirred solution of pyrrolopyrimidine **8** (250 mg, 1.63 mmol, 1.00 equiv) in CH_2_Cl_2_ (5.40 mL) were added 4-fluorophenylboronic acid (251 mg, 1.79 mmol, 1.10 equiv), Cu(OAc)_2_ (590 mg, 3.26 mmol, 2.00 equiv), and Et_3_N (2.27 mL, 16.3 mmol, 10.0 equiv). The solution was stirred 12 h at 90 °C. The solution was filtered through celite and H_2_O was added. The aqueous phase was extracted with CH_2_Cl_2_ (3 × 5.50 mL). The combined organic phase was washed with brine, dried over MgSO_4_ and concentrated *in vacuo*. The residue was purified by flash column chromatography on silica gel (EtOAc:hexanes = 1:4) to give *N*-4-fluorophenyl pyrrolopyrimidine **9e** (101 mg, 25% yield). ^1^H NMR (600 MHz, CDCl_3_) δ 8.70 (s, 1H), 7.70 − 7.63 (m, 2H), 7.27 − 7.23 (m, 2H), 7.50 (d, *J* = 3.7 Hz, 1H), 6.80 (d, *J* = 3.7 Hz, 1H).

#### 4.1.7. 4-Chloro-7–(4-methoxyphenyl)-7H-pyrrolo[2,3-d]pyrimidine (9f)

To a stirred solution of pyrrolopyrimidine **8** (100 mg, 0.651 mmol, 1.00 equiv) in CH_2_Cl_2_ (5.40 mL) were added 4-methoxyphenylboronic acid (110 mg, 0.716 mmol, 1.10 equiv), Cu(OAc)_2_ (240 mg, 1.30 mmol, 2.00 equiv), and Et_3_N (0.91 mL, 6.51 mmol, 10.0 equiv). The solution was stirred 12 h at 25 °C. The solution was filtered through celite and H_2_O was added. The aqueous phase was extracted with CH_2_Cl_2_ (3 × 5.50 mL). The combined organic phase was washed with brine, dried over MgSO_4_ and concentrated *in vacuo*. The residue was purified by flash column chromatography on silica gel (EtOAc:hexanes = 1:4) to give *N*-4-methoxyphenyl pyrrolopyrimidine **9f** (139 mg, 82% yield). ^1^H NMR (400 MHz, DMSO-d_6_) δ 8.67 (s, 1H), 8.06 (d, *J* = 3.7 Hz, 1H), 7.73 − 7.67 (m, 2H), 7.16 − 7.10 (m, 2H), 6.85 (d, *J* = 3.7 Hz, 1H), 3.83 (s, 3H); ^13^C NMR (101 MHz, DMSO-d_6_) δ 158.40, 151.12, 150.94, 150.29, 131.28, 129.61, 125.57, 117.49, 114.51, 99.81, 55.51; HRMS (MM: ESI-APCI+) *m/z* calc’d for C_13_H_10_ClN_3_ONa [M + Na]^+^: 282.0410; found: 282.0408.

#### 4.1.8. 4-Chloro-7-(furan-3-yl)-7H-pyrrolo[2,3-d]pyrimidine (9g)

To a stirred solution of pyrrolopyrimidine **8** (500 mg, 3.26 mmol, 1.00 equiv) in CH_2_Cl_2_ (11.0 mL) were added furan-3-boronic acid (401 mg, 3.59 mmol, 1.10 equiv), Cu(OAc)_2_ (1.18 g, 6.52 mmol, 2.00 equiv), and Et_3_N (4.54 mL, 32.6 mmol, 10.0 equiv). The solution was stirred 12 h at 90 °C. The solution was filtered through celite and H_2_O was added. The aqueous phase was extracted with CH_2_Cl_2_ (3 × 10.0 mL). The combined organic phase was washed with brine, dried over MgSO_4_ and concentrated *in vacuo*. The residue was purified by flash column chromatography on silica gel (EtOAc:hexanes = 1:5) to give *N*-furan pyrrolopyrimidine **9g** (415 mg, 58% yield). ^1^H NMR (400 MHz, CDCl_3_) δ 8.71 (s, 1H), 8.31 − 8.11 (m, 1H), 7.59 − 7.50 (m, 1H), 7.46 (d, *J* = 3.7 Hz, 1H), 6.87 (dd, *J* = 1.9, 0.9 Hz, 1H), 6.75 (d, *J* = 3.7 Hz, 1H).

#### 4.1.9. 4-Chloro-7-(pyridin-3-yl)-7H-pyrrolo[2,3-d]pyrimidine (9h)

To a stirred solution of pyrrolopyrimidine **8** (500 mg, 3.26 mmol, 1.00 equiv) in CH_2_Cl_2_ (11.0 mL) were added pyridine-3-boronic acid (441 mg, 3.59 mmol, 1.10 equiv), Cu(OAc)_2_ (1.18 g, 6.52 mmol, 2.00 equiv), and Et_3_N (4.54 mL, 32.6 mmol, 10.0 equiv). The solution was stirred 12 h at 90 °C. The solution was filtered through celite and H_2_O was added. The aqueous phase was extracted with CH_2_Cl_2_ (3 × 10.0 mL). The combined organic phase was washed with brine, dried over MgSO_4_ and concentrated *in vacuo*. The residue was purified by flash column chromatography on silica gel (EtOAc:hexanes = 1:5) to give *N*-pyridine pyrrolopyrimidine **9h** (571 mg, 76% yield). ^1^H NMR (400 MHz, CDCl_3_) δ 8.97 (s, 1H), 8.72 (s, 1H), 8.71 − 8.65 (m, 1H), 8.19 (ddd, *J* = 8.2, 2.7, 1.5 Hz, 1H), 7.59 (d, *J* = 3.7 Hz, 1H), 7.53 (dd, *J* = 8.3, 4.7 Hz, 1H), 6.86 (d, *J* = 3.7 Hz, 1H).

#### 4.1.10. 4-Chloro-7-cyclopropyl-7H-pyrrolo[2,3-d]pyrimidine (9i)

To a stirred solution of pyrrolopyrimidine **8** (90.0 mg, 0.586 mmol, 1.00 equiv) in CH_2_Cl_2_ (2.00 mL) were added cyclopropylboronic acid (55.4 mg, 0.645 mmol, 1.10 equiv), Cu(OAc)_2_ (160 mg, 0.879 mmol, 1.50 equiv), and pyridine (0.237 mL, 2.93 mmol, 5.00 equiv). The solution was stirred 12 h at 90 °C. The solution was filtered through celite and H_2_O was added. The aqueous phase was extracted with CH_2_Cl_2_ (3 × 2.50 mL). The combined organic phase was washed with brine, dried over MgSO_4_ and concentrated *in vacuo*. The residue was purified by flash column chromatography on silica gel (EtOAc:hexanes = 1:5) to give *N*-cyclopropyl pyrrolopyrimidine **9i** (113 mg, 78% yield). ^1^H NMR (400 MHz, CDCl_3_) δ 8.68 (s, 1H), 7.24 (d, *J* = 3.7 Hz, 1H), 6.55 (d, *J* = 3.6 Hz, 1H), 3.53 (tt, *J* = 7.1, 3.8 Hz, 1H), 1.22 − 1.16 (m, 2H), 1.11 − 1.06 (m, 2H).

#### 4.1.11. 5-Bromo-4-chloro-7H-pyrrolo[2,3-d]pyrimidine (S1)

*N*-Bromosuccinimide (2.70 g, 15.4 mmol, 1.18 equiv) was added portionwise to pyrrolopyrimidine **8** (1.00 g, 13.0 mmol, 1.00 equiv) in dry CH_2_Cl_2_ (50.0 mL) under nitrogen atmosphere. The resulting suspension was stirred for 1 h at 20 °C. The reaction mixture was evaporated and the resulting yellow solid was triturated with water to give a purple solid, which was collected by filtration. The solid was crystallised with MeOH to afford 5- bromo-4-chloro-7*H*-pyrrolo[2,3-*d*]pyrimidine (3.09 g, 100% yield). ^1^H NMR (400 MHz, DMSO-d_6_) δ 12.97 (s, 1H), 8.63 (s, 1H), 7.95 (d, *J* = 2.6 Hz, 1H).

#### 4.1.12. 4-Chloro-5-methyl-7H-pyrrolo[2,3-d]pyrimidine (9j)

*n*-BuLi (2.5 M in hexanes; 3.8 mL, 9.5 mmol, 2.2 equiv) was added dropwise to a solution 5-bromo-4-chloro-7*H*- pyrrolo[2,3-*d*]pyrimidine **S1** (1.00 g, 4.30 mmol, 1.00 equiv) in THF (24 mL) at −78 °C over a period of 5 min under nitrogen atmosphere. After the reaction mixture was stirred for 30 min at −78 °C, methyl iodide (0.428 mL, 6.88 mmol, 1.60 equiv) was added. The reaction mixture was stirred for 1 h at 25 °C then diluted with water (25 mL), and extracted with EtOAc (3 × 20 mL). The organic layer was washed with brine (30 mL) then dried over MgSO_4_, and concentrated *in vacuo*. The residue was purified by flash column chromatography (40% EtOAc in hexane) on silica gel to give 4-chloro-5-methyl-7*H*-pyrrolo[2,3-*d*]pyrimidine (187 mg, 23% yield). ^1^H NMR (400 MHz, DMSO-d_6_) δ 8.68 (s, 1H), 7.99 (s, 1H), 3.83 (s, 3H).

#### 4.1.13. General procedure B1 for synthesis of piperidine substituted sulfamides 18

##### 4.1.13.1. Step 1: S_N_Ar reaction

To a solution of pyrrolopyrimidine **12** (1.00 equiv) and amine **13** (1.30 equiv) in *n*-butanol (0.100 M) was added *N,N*-diisopropylethylamine (3.00 equiv) under nitrogen atmosphere. After the reaction mixture was stirred for 16 h at 100 °C, the reaction mixture was cooled down to 25 °C. The solid was filtered to obtain intermediate **14**, or water was poured into the mixture and extracted with CH_2_Cl_2_. The combined organic phases were washed with brine, dried over Na_2_SO_4_ and concentrated *in vacuo*. The residue was purified by flash column chromatography (1:20 MeOH:CH_2_Cl_2_) on silica gel. The collected fraction was then used in the next step (45 − 97% yield).

##### 4.1.13.2. Step 2: Boc deprotection

To a solution of collected fraction (1.00 equiv) in CH_2_Cl_2_ (0.041 M) was added hydrochloric acid (4 M in 1,4-dioxane, 0.200 M) at 0 °C. The temperature of the reaction mixture was gradually increased to 25 °C. The reaction mixture was stirred for 16 h and concentrated *in vacuo*. The crude mixture was then filtered and washed with Et_2_O and CH_2_Cl_2_ to afford intermediate **15** (72 − 100% yield).

##### 4.1.13.3. Step 3: Sulfamoylation reaction

To a solution of intermediate **15** (1.00 equiv) and (tert-butoxycarbonyl)((4-(dimethylamino)pyridin-1-ium-1-yl)sulfonyl)amide **16** (1.10 equiv) in CH_2_Cl_2_ (0.0873 M) was added Et_3_N (3.00 equiv) at 0 °C. The temperature of the reaction mixture was gradually increased to 25 °C and the mixture was stirred for 16 h. The solution was then quenched with water and extracted with CH_2_Cl_2_. The combined organic phases were washed with brine, dried over Na_2_SO_4_ and concentrated *in vacuo*. The residue was purified by flash column chromatography (1:20 MeOH:CH_2_Cl_2_) or Prep TLC (1:20 MeOH:CH_2_Cl_2_). The obtained compound was then used in the next step (8 − 88% yield).

##### 4.1.13.4. Step 4: Boc deprotection

To a solution of collected fraction (1.00 equiv) in CH_2_Cl_2_ (0.041 M) was added hydrochloric acid (4 M in 1,4-dioxane, 0.200 M) at 0 °C. The temperature of the reaction mixture was gradually increased to 25 °C. The reaction mixture was stirred for 16 h and concentrated *in vacuo*. The crude mixture was then filtered and washed with Et_2_O and CH_2_Cl_2_ to afford title compound **18** (21 − 98% yield).

#### 4.1.14. General procedure B2 for synthesis of sulfone amides 20

To a flame dried two-neck 10-mL round-bottom flask was added amine **15** (1.00 equiv). The flask was covered with a septum, and then placed under vacuum and refilled with N_2_ (three cycles). After the addition of anhydrous CH_2_Cl_2_ (0.0800 M) to the flask, Et_3_N (4.50 equiv) and RSO_2_Cl (1.05 equiv) were added. The reaction mixture was stirred for 16 h at 25 °C. The reaction mixture was stirred until no starting material remained by TLC. After completion of the reaction, a water (10.0 mL) was added. The aqueous phase was extracted with CH_2_Cl_2_ (3 × 10 mL). The combined organic phases were washed with brine, dried over anhydrous Na_2_SO_4_, filtered, and concentrated *in vacuo*. The residue was purified by flash column chromatography (1:20 MeOH:CH_2_Cl_2_) to afford sulfone amides **20** (22 − 55% yield).

**Note**: This procedure is the same as step1 and step 2 of **General procedure B1.**

#### 4.1.15. General Procedure C: Synthesis of aryl substituted sulfamide 25

##### 4.1.15.1. Step 1: Suzuki reaction

Procedure 1) To a solution of pyrrolopyrimidine **12** (1.00 equiv) in 1,4-dioxane (0.090 M) and water (0.90 M) was added boronic acid **21** (1.05 equiv) and potassium carbonate (3.75 equiv) under nitrogen atmosphere. Tetrakis(triphenylphosphine) palladium(0) (Pd(PPh_3_)_4_, 0.060 equiv) was added into reaction mixture and purged by nitrogen for 5 min. After the reaction mixture was stirred for 24 h at 110 °C, the reaction mixture was cooled down to 25 °C and quenched with water (5.00 mL). The reaction mixture was then filtered through a pad of Celite and extracted with EtOAc. The combined organic phases were washed with brine, dried over Na_2_SO_4_ and concentrated *in vacuo*. The residue was purified by flash column chromatography on silica gel (40 − 89% yield).

Procedure 2) To a solution of pyrrolopyrimidine **12** (1.00 equiv) and boronic acid **21** (1.05 equiv) in 1,4-dioxane (0.300 M) was added 1,1′-bis(diphenylphosphino)ferrocene-palladium(II) dichloride (PdCl_2_(dppf), 0.100 equiv), and potassium phosphate (1.50 equiv) under nitrogen atmosphere. After the reaction mixture was stirred for 24 h at 110 °C, the reaction mixture was cooled down to 25 °C and quenched with water (5.00 mL). The reaction mixture was then filtered through a pad of Celite and extracted with CH_2_Cl_2_. The combined organic phases were washed with brine, dried over Na_2_SO_4_ and concentrated *in vacuo*. The residue was purified by flash column chromatography on silica gel (74% yield).

##### 4.1.15.2. Step 2: Boc deprotection

To a collected fraction (1.00 equiv) in CH_2_Cl_2_ (0.0410 M) was added hydrochloric acid (4 M in 1,4-dioxane, 0.200 M) at 0 °C. The temperature of the reaction mixture was gradually increased to 25 °C. The reaction mixture was stirred for 16 h and concentrated *in vacuo*. The crude mixture was then filtered and washed with diethyl ether to afford intermediate **23** (76 − 100% yield).

##### 4.1.15.3. Step 3: Sulfamoylation reaction

To a solution of intermediate **23** (1.00 equiv) and (tert-butoxycarbonyl)((4-(dimethylamino)pyridin-1-ium-1-yl)sulfonyl)amide **16** (1.10 equiv) in CH_2_Cl_2_ (0.0873 M) was added Et_3_N (3.00 equiv) at 0 °C. The temperature of the reaction mixture was gradually increased to 25 °C and the mixture was stirred for 16 h. This solution was then quenched with water and extracted with CH_2_Cl_2_. The combined organic phases were washed with brine, dried over Na_2_SO_4_ and concentrated *in vacuo*. The residue was purified by flash column chromatography on silica gel or Prep TLC (1:20 MeOH:CH_2_Cl_2_). The collected fraction was then used directly in the next step (15 − 52% yield).

##### 4.1.15.4. Step 4: Boc deprotection

To a solution of collected fraction (1.00 equiv) in CH_2_Cl_2_ (0.040 M) was added hydrochloric acid (4 M in 1,4-dioxane, 0.200 M) at 0 °C. The temperature of the reaction mixture was gradually increased to 25 °C. The reaction mixture was stirred for 16 h and concentrated *in vacuo*. The crude mixture was then filtered and washed with diethyl ether to afford title compound **25** (40–100% yield).

#### 4.1.16. Synthesis of sulfamide 16

To a cold solution of *t*-butanol (6.55 mL, 68.5 mmol, 1.00 equiv) in anhydrous CH_2_Cl_2_ (50.0 mL, 1.37 M) was added Chlorosulfonyl isocyanate (5.96 mL, 68.5 mmol, 1.00 equiv) under nitrogen atmosphere. Then, DMAP (16.7 g, 137 mmol, 2.00 equiv) was added to the reaction mixture. After the reaction mixture was stirred for 17 h at 25 °C, the mixture several times washed with H_2_O, dried over anhydrous Na_2_SO_4_, and concentrated *in vacuo*. The solid was crystallised to a white solid from acetonitrile (18.3 g, 89% yield). ^1^H NMR (400 MHz, DMSO-d_6_) δ 8.46 (d, *J* = 8.0 Hz, 2H), 6.97 (d, *J* = 8.0 Hz, 2H), 3.23 (s, 6H), 1.26 (s, 9H).

#### 4.1.17. N-(2–(1-(7H-Pyrrolo[2,3-d]pyrimidin-4-yl)piperidin-4-yl)ethyl)methanesulfamide hydrochloride (18a)

According to step 1 of **General Procedure B1** starting from pyrrolopyrimidine **8 (**120 mg, 0.781 mmol, 1.00 equiv) and amine **13a** (232 mg, 1.02 mmol, 1.30 equiv), the intermediate **14a** was obtained (230 mg, 84% yield). Removal of the Boc group of **14a** (150 mg, 0.434 mmol) under acidic condition provided amine **15a** (120 mg, 98% yield). Then, **15a** (30.0 mg, 0.106 mmol) was used directly in the next step to give sulfamide **17a** (12.1 mg, 27% yield). Cleavage of the Boc group of **17a** (10.7 mg, 0.0252 mmol) generated **18a** (5.7 mg, 63% yield, Purity: 98%; HPLC). ^1^H NMR (400 MHz, D_2_O) δ 7.98 (s, 1H), 7.08 (d, *J* = 3.6 Hz, 1H), 6.54 (d, *J* = 3.6 Hz, 1H), 4.42 (d, *J* = 13.3 Hz, 2H), 3.09 − 2.96 (m, 4H), 1.81 − 1.59 (m, 3H), 1.42 (q, *J* = 7.1 Hz, 2H), 1.13 (qd, *J* = 12.5, 4.2 Hz, 2H); ^13^C NMR (101 MHz, MeOD) δ 152.89, 143.55, 141.88, 124.97, 105.31, 103.19, 49.08, 41.41, 36.64, 33.71, 32.76; HRMS (MM: ESI-APCI+) *m/z* calc’d for C_13_H_21_N_6_O_2_S [M + H]^+^: 325.1447; found: 325.1441.

#### 4.1.18. N-(2–(1-(2-(methylthio)-7H-pyrrolo[2,3-d]pyrimidin-4-yl)piperidin-4-zyl)ethyl)methanesulfamide hydrochloride (18b)

According to step 1 of **General Procedure B1** starting from pyrrolopyrimidine **12 (**120 mg, 0.601 mmol, 1.00 equiv) and amine **13b** (178 mg, 0.781 mmol, 1.30 equiv), the intermediate **14b** was obtained (195 mg, 83% yield). Removal of the Boc group of **14b** (120 mg, 0.306 mmol) under acidic condition provided amine **15b** (100 mg, 100% yield). Then, **15b** (60.0 mg, 0.183 mmol) was used directly in the next step to give sulfamide **17b** (55.9 mg, 65% yield). Cleavage of the Boc group of **17b** (20.0 mg, 0.0425 mmol) generated **18b** (12.6 mg, 73% yield, Purity: 96%; HPLC). ^1^H NMR (400 MHz, MeOD) δ 6.94 (d, *J* = 3.7 Hz, 1H), 6.50 (d, *J* = 3.7 Hz, 1H), 4.81 (d, *J* = 13.3 Hz, 2H), 3.16 − 2.98 (m, 4H), 2.52 (s, 3H), 1.91 − 1.75 (m, 3H), 1.54 (q, *J* = 7.0 Hz, 2H), 1.35 − 1.20 (m, 2H); ^13^C NMR (101 MHz, MeOD) δ 157.56, 153.99, 153.95, 153.92, 121.24, 103.55, 99.41, 40.06, 35.32, 32.62, 31.67, 12.78; HRMS (MM: ESI-APCI+) *m/z* calc’d for C_14_H_23_N_6_O_2_S_2_ [M + H]^+^: 371.1324; found: 371.1318.

#### 4.1.19. N-((1–(7H-Pyrrolo[2,3-d]pyrimidin-4-yl)piperidin-4-yl)methyl)methanesulfamide hydrochloride (18c)

According to step 1 of **General Procedure B1** starting from pyrrolopyrimidine **8** (120 mg, 0.781 mmol, 1.00 equiv) and amine **13c** (218 mg, 1.02 mmol, 1.30 equiv), the intermediate **14c** was obtained (178 mg, 69% yield). Removal of the Boc group of **14c** (122 mg, 0.369 mmol) under acidic condition provided amine **15c** (78.8 mg, 79% yield). Then, **15c** (37.1 mg, 0.139 mmol) was used directly in the next step to give sulfamide **17c** (30.9 mg, 54% yield). Cleavage of the Boc group of **17c** (20.0 mg, 0.0487 mmol) generated **18c** (12.1 mg, 72% yield, Purity: 99%; HPLC). ^1^H NMR (400 MHz, MeOD) δ 8.24 (s, 1H), 7.37 (d, *J* = 3.6 Hz, 1H), 6.93 (d, *J* = 3.6 Hz, 1H), 4.65 (d, *J* = 13.0 Hz, 2H), 3.44 (t, *J* = 13.1 Hz, 2H), 2.99 (d, *J* = 6.3 Hz, 2H), 2.12 − 1.98 (m, 3H), 1.52 − 1.32 (m, 2H); ^13^C NMR (101 MHz, DMSO-d_6_) δ 143.22, 124.01, 123.99, 103.95, 101.31, 66.34, 48.56, 47.44, 47.26, 34.63, 29.17; HRMS (MM: ESI-APCI+) *m/z* calc’d for C_12_H_19_N_6_O_2_S [M + H]^+^: 311.1290; found: 311.1284.

#### 4.1.20. N-((1–(7-Methyl-7H-pyrrolo[2,3-d]pyrimidin-4-yl)piperidin-4-yl)methyl)methanesulfamide hydrochloride (18d)

According to step 1 of **General Procedure B1** starting from pyrrolopyrimidine **9a** (86.0 mg, 0.513 mmol, 1.00 equiv) and amine **13d** (143 mg, 0.667 mmol, 1.30 equiv), the intermediate **14d** was obtained (136 mg, 77% yield). Removal of the Boc group of **14d** (125 mg, 0.362 mmol) under acidic condition provided amine **15d** (89.5 mg, 88% yield). Then, **15d** (68.1 mg, 0.242 mmol) was used directly in the next step to give sulfamide **17d** (41.3 mg, 41% yield). Cleavage of the Boc group of **17d** (14.0 mg, 0.0330 mmol) generated **18d** (19.6 mg, 78% yield, Purity: 100%; HPLC). ^1^H NMR (400 MHz, MeOD) δ 8.27 (s, 1H), 7.43 (d, *J* = 3.6 Hz, 1H), 6.97 (d, *J* = 3.7 Hz, 1H), 4.53 (d, *J* = 13.0 Hz, 2H), 3.89 (s, 3H), 3.50 (t, *J* = 12.2 Hz, 2H), 3.00 (d, *J* = 6.3 Hz, 2H), 2.20 − 1.96 (m, 3H), 1.59 − 1.38 (m, 2H); ^13^C NMR (101 MHz, DMSO-d_6_) δ 143.43, 128.10, 103.30, 101.40, 69.77, 62.79, 47.44, 39.99, 34.59, 31.88, 29.13; HRMS (MM: ESI-APCI+) *m/z* calc’d for C_13_H_21_N_6_O_2_S [M + H]^+^: 325.1447; found: 325.1441.

#### 4.1.21. N-((1–(7-Isopropyl-7H-pyrrolo[2,3-d]pyrimidin-4-yl)piperidin-4-yl)methyl)methanesulfamide hydrochloride (18e)

According to step 1 of **General Procedure B1** starting from pyrrolopyrimidine **9b** (95.0 mg, 0.486 mmol, 1.00 equiv) and amine **13e** (135 mg, 0.631 mmol, 1.30 equiv), the intermediate **14e** was obtained (149 mg, 82% yield). Removal of the Boc group of **14e** (93.0 mg, 0.249 mmol) under acidic condition provided amine **15e** (77.5 mg, 100% yield). Then, **15e** (63.2 mg, 0.204 mmol) was used directly in the next step to give sulfamide **17e** (38.8 mg, 42% yield). Cleavage of the Boc group of **17e** (18.7 mg, 0.0413 mmol) generated **18e** (7.6 mg, 47% yield, Purity: 99%; HPLC). ^1^H NMR (400 MHz, MeOD) δ 8.26 (s, 1H), 7.60 (d, *J* = 3.8 Hz, 1H), 7.01 (d, *J* = 3.8 Hz, 1H), 5.12 (p, *J* = 6.7 Hz, 1H), 4.52 (d, *J* = 13.3 Hz, 2H), 3.51 (t, *J* = 12.8 Hz, 2H), 3.00 (d, *J* = 6.3 Hz, 2H), 2.22 − 1.96 (m, 3H), 1.54 (d, *J* = 6.8 Hz, 6H), 1.52 − 1.41 (m, 2H); ^13^C NMR (101 MHz, DMSO-d_6_) δ 150.39, 146.61, 143.50, 124.06, 104.00, 101.76, 47.65, 47.60, 46.88, 34.80, 29.26, 22.43; HRMS (MM: ESI-APCI+) *m/z* calc’d for C_15_H_25_N_6_O_2_S [M + H]^+^: 353.1760; found: 353.1754.

#### 4.1.22. N-((1–(7-Cyclopropyl-7H-pyrrolo[2,3-d]pyrimidin-4-yl)piperidin-4-yl)methyl)methanesulfamide hydrochloride hydrochloride (18f)

According to step 1 of **General Procedure B1** starting from pyrrolopyrimidine **9i** (100 mg, 0.516 mmol, 1.00 equiv) and amine **13f** (144 mg, 0.671 mmol, 1.30 equiv), the intermediate **14f** was obtained (183 mg, 95% yield). Removal of the Boc group of **14f** (150 mg, 0.404 mmol) under acidic condition provided amine **15f** (105 mg, 84% yield). Then, **15f** (80.0 mg, 0.260 mmol) was used directly in the next step to give sulfamide **17f** (49.9 mg, 43% yield). Cleavage of the Boc group of **17f** (20.0 mg, 0.0443 mmol) generated **18f** (12.0 mg, 70% yield, Purity: 98%; HPLC). ^1^H NMR (400 MHz, MeOD) δ 8.32 (s, 1H), 7.38 (d, *J* = 3.4 Hz, 1H), 6.90 (d, *J* = 3.5 Hz, 1H), 4.64 (d, *J* = 12.0 Hz, 2H), 3.64 − 3.56 (m, 1H), 3.46 (t, *J* = 12.5 Hz, 2H), 2.99 (d, *J* = 6.1 Hz, 2H), 2.26 − 1.80 (m, 3H), 1.49 − 1.37 (m, 2H), 1.24 − 1.13 (m, 2H), 1.14 − 1.05 (m, 2H); ^13^C NMR (201 MHz, MeOD) δ 158.15, 152.93, 151.63, 125.07, 104.99, 101.90, 49.55, 47.21, 37.63, 31.01, 27.67, 6.86; HRMS (MM: ESI-APCI+) *m/z* calc’d for C_15_H_23_N_6_O_2_S [M + H]^+^: 351.1603; found: 351.1598.

#### 4.1.23. N-((1–(7-Phenyl-7H-pyrrolo[2,3-d]pyrimidin-4-yl)piperidin-4-yl)methyl)methanesulfamide hydrochloride hydrochloride (18g)

According to step 1 of **General Procedure B1** starting from pyrrolopyrimidine **9d** (116 mg, 0.505 mmol, 1.00 equiv) and amine **13g** (141 mg, 0.657 mmol, 1.30 equiv), the intermediate **14g** was obtained (175 mg, 85% yield). Removal of the Boc group of **14g** (160 mg, 0.393 mmol) under acidic condition provided amine **15g** (120 mg, 89% yield). Then, **15g** (100 mg, 0.291 mmol) was used directly in the next step to give sulfamide **17 g** (109 mg, 88% yield). Cleavage of the Boc group of **17 g** (25.4 mg, 0.0522 mmol) generated **18g** (9.9 mg, 45% yield, Purity: 100%; HPLC). ^1^H NMR (400 MHz, MeOD) δ 8.25 (s, 1H), 7.70 − 7.64 (m, 3H), 7.60 (t, *J* = 7.6 Hz, 2H), 7.52 (t, *J* = 7.3 Hz, 1H), 7.15 (d, *J* = 3.8 Hz, 1H), 4.60 (d, *J* = 13.3 Hz, 2H), 3.56 − 3.49 (m, 2H), 3.02 (d, *J* = 6.5 Hz, 2H), 2.14–2.01 (m, 3H), 1.57 − 1.44 (m, 2H); ^13^C NMR (101 MHz, DMSO-d_6_) δ 147.64, 136.37, 129.40, 127.91, 127.13, 127.08, 127.04, 124.85, 104.88, 102.75, 47.71, 47.44, 34.57, 29.13; HRMS (MM: ESI-APCI+) *m/z* calc’d for C_18_H_23_N_6_O_2_S [M + H]^+^: 387.1603; found: 387.1598.

#### 4.1.24. N-((1–(7-(4-Fluorophenyl)-7H-pyrrolo[2,3-d]pyrimidin-4-yl)piperidin-4-yl)methyl)methanesulfamide hydrochloride (18h)

According to step 1 of **General Procedure B1** starting from pyrrolopyrimidine **9e** (95.0 mg, 0.384 mmol, 1.00 equiv) and amine **13h** (107 mg, 0.499 mmol, 1.30 equiv), the intermediate **14h** was obtained (141 mg, 86% yield). Removal of the Boc group of **14h** (120 mg, 0.282 mmol) under acidic condition provided amine **15h** (100 mg, 98% yield). Then, **15h** (90.0 mg, 0.249 mmol) was used directly in the next step to give sulfamide **17h** (59.7 mg, 47% yield). Cleavage of the Boc group of **17h** (40.7 mg, 0.0806 mmol) generated **18h** (25.9 mg, 73% yield, Purity: 98%; HPLC). ^1^H NMR (400 MHz, MeOD) δ 8.28 (s, 1H), 7.73 − 7.62 (m, 3H), 7.34 (t, *J* = 8.6 Hz, 2H), 7.18 (d, *J* = 3.8 Hz, 1H), 4.57 (d, *J* = 13.3 Hz, 2H), 3.57 (t, *J* = 12.0 Hz, 2H), 3.02 (d, *J* = 6.3 Hz, 2H), 2.21 − 1.93 (m, 3H), 1.63 − 1.42 (m, 2H); ^13^C NMR (101 MHz, MeOD) δ 164.92, 162.47, 151.59, 143.71, 133.85 (d, *J* = 3.1 Hz), 128.94, 128.48 (d, *J* = 8.8 Hz), 117.39 (d, *J* = 23.4 Hz), 106.22, 104.43, 49.29, 48.90, 36.39, 30.37; ^19 ^F NMR (377 MHz, MeOD) δ −114.82; HRMS (MM: ESI-APCI+) *m/z* calc’d for C_18_H_22_FN_6_O_2_S [M + H]+: 405.1509; found: 405.1503.

#### 4.1.25. N-((1–(7-(4-Methoxyphenyl)-7H-pyrrolo[2,3-d]pyrimidin-4-yl)piperidin-4-yl)methyl)methanesulfamide hydrochloride (18i)

According to step 1 of **General Procedure B1** starting from pyrrolopyrimidine **9f** (36.7 mg, 0.141 mmol, 1.00 equiv) and amine **13i** (33.3 mg, 0.155 mmol, 1.10 equiv), the intermediate **14i** was obtained (60.0 mg, 97% yield). Removal of the Boc group of **14i** under acidic condition provided amine **15i** (41.0 mg, 80% yield). Then, **15i** (32.3 mg, 0.0957 mmol) was used directly in the next step to give sulfamide **17i** (19.6 mg, 40% yield). Cleavage of the Boc group of **17i** generated **18i** (11.7 mg, 68% yield, Purity: 99%; HPLC). ^1^H NMR (400 MHz, MeOD) δ 8.26 (s, 1H), 7.63 (d, *J* = 3.6 Hz, 1H), 7.54 (d, *J* = 8.9 Hz, 2H), 7.14 (d, *J* = 3.7 Hz, 1H), 7.12 (d, *J* = 8.9 Hz, 2H), 4.60 (d, *J* = 12.7 Hz, 2H), 3.88 (s, 3H), 3.55 (t, *J* = 11.9 Hz, 2H), 3.02 (d, *J* = 6.2 Hz, 2H), 2.17 − 1.99 (m, 3H), 1.59 − 1.42 (m, 2H); ^13^C NMR (101 MHz, MeOD) δ 161.32, 151.94, 147.70, 143.52, 130.35, 129.17, 127.71, 115.78, 105.85, 104.16, 56.17, 48.92, 36.46, 30.43; HRMS (MM: ESI-APCI+) *m/z* calc’d for C_19_H_25_N_6_O_3_S [M + H]^+^: 417.1709; found: 417.1703.

#### 4.1.26. N-((1–(7-Benzyl-7H-pyrrolo[2,3-d]pyrimidin-4-yl)piperidin-4-yl)methyl)methanesulfamide hydrochloride (18j)

According to step 1 of **General Procedure B1** starting from pyrrolopyrimidine **9c** (195 mg, 0.800 mmol, 1.00 equiv) and amine **13j** (223 mg, 1.04 mmol, 1.30 equiv), the intermediate **14j** was obtained (264 mg, 78% yield). Removal of the Boc group of **14j** (227 mg, 0.538 mmol) under acidic condition provided amine **15j** (191 mg, 99% yield). Then, **15j** (150 mg, 0.419 mmol) was used directly in the next step to give sulfamide **17j** (167 mg, 80% yield). Cleavage of the Boc group of **17j** generated **18j** (30.8 mg, 21% yield, Purity: 98%; HPLC). ^1^H NMR (400 MHz, MeOD) δ 8.16 (s, 1H), 7.30 − 7.20 (m, 3H), 7.15 (d, *J* = 7.0 Hz, 2H), 7.09 (d, *J* = 3.7 Hz, 1H), 6.62 (d, *J* = 3.7 Hz, 1H), 5.36 (s, 2H), 4.73 (d, *J* = 13.3 Hz, 2H), 3.11 (t, *J* = 11.9 Hz, 2H), 2.94 (d, *J* = 6.3 Hz, 2H), 1.95–1.80 (m, 3H), 1.33 − 1.20 (m, 2H); ^13^C NMR (101 MHz, MeOD) δ 143.36, 138.22, 137.83, 129.91, 129.23, 128.78, 128.67, 108.62, 108.25, 105.47, 103.48, 49.76, 49.15, 49.00, 48.91, 41.03, 40.23, 36.45, 30.34, 28.02; HRMS (MM: ESI-APCI+) *m/z* calc’d for C_19_H_25_N_6_O_2_S [M + H]^+^: 401.1760; found: 401.1755.

#### 4.1.27. N-((1–(7-(Furan-3-yl)-7H-pyrrolo[2,3-d]pyrimidin-4-yl)piperidin-4-yl)methyl)methanesulfamide hydrochloride (18k)

According to step 1 of **General Procedure B1** starting from pyrrolopyrimidine **9g** (39.4 mg, 0.179 mmol, 1.00 equiv) and amine **13k** (50.0 mg, 0.233 mmol, 1.30 equiv), the intermediate **14k** was obtained (54.7 mg, 77% yield). Removal of the Boc group of **14k** under acidic condition provided amine **15k** (42.7 mg, 93% yield). Then, **15k** was used directly in the next step to give sulfamide **17k** (31.4 mg, 52% yield). Cleavage of the Boc group of **17k** generated **18k** (18.4 mg, 74% yield, Purity: 97%; HPLC). ^1^H NMR (400 MHz, MeOD) δ 8.34 (s, 1H), 8.30 (dd, *J* = 1.6, 0.8 Hz, 1H), 7.76 (d, *J* = 3.8 Hz, 1H), 7.67 (t, *J* = 1.8 Hz, 1H), 7.16 (d, *J* = 3.9 Hz, 1H), 7.07 (dd, *J* = 2.0, 0.9 Hz, 1H), 4.52 (d, *J* = 13.5 Hz, 2H), 3.59 − 3.51 (m, 2H), 3.02 (d, *J* = 6.4 Hz, 2H), 2.15 − 2.01 (m, 3H), 1.58 − 1.44 (m, 2H); ^13^C NMR (101 MHz, MeOD) δ 151.22, 148.46, 144.81, 143.89, 136.47, 127.62, 125.91, 107.14, 106.43, 104.50, 49.28, 49.00, 48.88, 36.37, 30.30; HRMS (MM: ESI-APCI+) *m/z* calc’d for C_16_H_21_N_6_O_3_S [M + H]^+^: 377.1396; found: 377.1395.

#### 4.1.28. N-((1-(7-(Pyridin-3-yl)-7H-pyrrolo[2,3-d]pyrimidin-4-yl)piperidin-4-yl)methyl)methanesulfamide hydrochloride (18l)

According to step 1 of **General Procedure B1** starting from pyrrolopyrimidine **9h** (56.4 mg, 0.244 mmol, 1.00 equiv) and amine **13l** (68.0 mg, 0.318 mmol, 1.30 equiv), the intermediate **14l** was obtained (44.7 mg, 45% yield). Removal of the Boc group of **14l** under acidic condition provided amine **15l** (27.2. mg, 72% yield). Then, **15l** was used directly in the next step to give sulfamide **17l** (20.3 mg, 53% yield). Cleavage of the Boc group of **17l** generated **18l** (11.4 mg, 65% yield, Purity: 98%; HPLC). ^1^H NMR (400 MHz, MeOD) δ 9.49 (d, *J* = 1.7 Hz, 1H), 8.98 (d, *J* = 8.5 Hz, 1H), 8.92 (d, *J* = 5.5 Hz, 1H), 8.39 (s, 1H), 8.22 (dd, *J* = 8.5, 5.6 Hz, 1H), 8.01 (d, *J* = 3.9 Hz, 1H), 7.35 (d, *J* = 3.9 Hz, 1H), 4.54 (d, *J* = 13.2 Hz, 2H), 3.61 (t, *J* = 12.1 Hz, 2H), 3.03 (d, *J* = 6.3 Hz, 2H), 2.19 − 2.02 (m, 3H), 1.63 − 1.49 (m, 2H); ^13^C NMR (101 MHz, MeOD) δ 151.15, 149.76, 149.59, 144.78, 143.37, 141.04, 140.72, 137.22, 128.69, 127.57, 108.06, 105.59, 49.56, 36.24, 30.24; HRMS (MM: ESI-APCI+) *m/z* calc’d for C_17_H_22_N_7_O_2_S [M + H]^+z^: 388.1556; found: 388.1552.

#### 4.1.29. N-((1–(5-Methyl-7H-pyrrolo[2,3-d]pyrimidin-4-yl)piperidin-4-yl)methyl)methanesulfamide hydrochloride (18m)

According to step 1 of **General Procedure B1** starting from pyrrolopyrimidine **9j** (100 mg, 0.597 mmol, 1.00 equiv) and amine **13 m** (166 mg, 0.776 mmol, 1.30 equiv), the intermediate **14m** was obtained (151 mg, 73% yield). Removal of the Boc group of **14m** (134 mg, 0.388 mmol) under acidic condition provided amine **15m** (94.3 mg, 86% yield). Then, **15m** (80.0 mg, 0.284 mmol) was used directly in the next step to give sulfamide **17 m** (58.0 mg, 48% yield). Cleavage of the Boc group of **17 m** (50.0 mg, 0.118 mmol) generated 1**8m** (25.7 mg, 60% yield, Purity: 98%; HPLC). ^1^H NMR (400 MHz, MeOD) δ 8.26 (s, 1H), 7.33 (s, 1H), 4.29 (d, *J* = 13.2 Hz, 2H), 3.77 (s, 3H), 3.07 − 2.95 (m, 4H), 1.92 (d, *J* = 13.0 Hz, 2H), 1.89 − 1.75 (m, 1H), 1.57 − 1.41 (m, 2H); ^13^C NMR (101 MHz, MeOD) δ 143.46, 138.21, 132.03, 108.62, 104.64, 90.60, 52.56, 41.02, 36.49, 32.55, 30.69; HRMS (MM: ESI-APCI+) *m/z* calc’d for C_13_H_21_N_6_O_2_S [M + H]^+^: 325.1447; found: 325.1441.

#### 4.1.30. N-((1–(6-Methyl-7H-pyrrolo[2,3-d]pyrimidin-4-yl)piperidin-4-yl)methyl)methanesulfamide hydrochloride (18n)

According to step 1 of **General Procedure B1** starting from pyrrolopyrimidine **12** (120 mg, 0.716 mmol, 1.00 equiv) and amine **13n** (199 mg, 0.931 mmol, 1.30 equiv), the intermediate **14n** was obtained (209 mg, 85% yield). Removal of the Boc group of **14n** (150 mg, 0.434 mmol) under acidic condition provided amine **15n** (100 mg, 82% yield). Then, **15n** (80.0 mg, 0.326 mmol) was used directly in the next step to give sulfamide **17n** (56.0 mg, 40% yield). Cleavage of the Boc group of **17n** (50.0 mg, 0.118 mmol) generated **18n** (19.2 mg, 45% yield, Purity: 98%; HPLC). ^1^H NMR (400 MHz, MeOD) δ 8.19 (s, 1H), 6.64 (s, 1H), 4.57 (d, *J* = 12.2 Hz, 2H), 3.44 (t, *J* = 12.3 Hz, 2H), 2.99 (d, *J* = 6.3 Hz, 2H), 2.45 (s, 3H), 2.09–1.94 (m, 3H), 1.52–1.36 (m, 2H); ^13^C NMR (101 MHz, DMSO-d_6_) δ 142.08, 134.71, 101.71, 101.31, 66.35, 48.56, 47.45, 47.31, 34.68, 29.22, 12.91; HRMS (MM: ESI-APCI+) *m/z* calc’d for C_13_H_21_N_6_O_2_S [M + H]^+^: 325.1447; found: 325.1441.

#### 4.1.31. N-(1–(1-(7H-Pyrrolo[2,3-d]pyrimidin-4-yl)piperidin-4-yl)ethyl)methanesulfamide hydrochloride (18o)

According to step 1 of **General Procedure B1** starting from pyrrolopyrimidine **8** (114 mg, 0.741 mmol, 1.00 equiv) and amine **13o** (186 mg, 0.186 mmol, 1.10 equiv), the intermediate **14o** was obtained (240 mg, 94% yield). Removal of the Boc group of **14o** (216 mg, 0.625 mmol) under acidic condition provided amine **15o** (143 mg, 81% yield). Then, **15o** (120 mg, 0.426 mmol) was used directly in the next step to give sulfamide **17o** (78.4 mg, 43% yield). Cleavage of the Boc group of **17o** (53.7 mg, 0.126 mmol) generated **18o** (34.1 mg, 75% yield, Purity: 100%; HPLC). ^1^H NMR (400 MHz, D_2_O) δ 8.02 (s, 1H), 7.20 (t, *J* = 11.9 Hz, 1H), 6.65 (d, *J* = 3.0 Hz, 1H), 4.41 (d, *J* = 12.8 Hz, 2H), 3.25 − 3.09 (m, 2H), 1.94 (d, *J* = 12.8 Hz, 1H), 1.85 − 1.66 (m, 2H), 1.51 − 1.20 (m, 3H), 1.16 (d, *J* = 6.6 Hz, 3H); ^13^C NMR (151 MHz, DMSO-d_6_) δ 143.13, 124.09, 104.09, 101.26, 52.27, 47.63, 47.53, 40.42, 28.02, 27.58, 18.26; HRMS (MM: ESI-APCI+) *m/z* calc’d for C_13_H_21_N_6_O_2_S [M + H]^+^: 325.1447; found: 325.1442.

#### 4.1.32. N-((4-Methyl-1-(7H-pyrrolo[2,3-d]pyrimidin-4-yl)piperidin-4-yl)methyl)methanesulfamide hydrochloride (18p)

According to step 1 of **General Procedure B1** starting from pyrrolopyrimidine **8** (534 mg, 3.48 mmol, 1.00 equiv) and amine **13p** (873 mg, 3.82 mmol, 1.10 equiv), the intermediate **14p** was obtained (1.19 g, 99% yield). Removal of the Boc group of **14p** (425 mg, 1.23 mmol) under acidic condition provided amine **15p** (347 mg, 100% yield). Then, **15p** (340 mg, 1.39 mmol) was used directly in the next step to give sulfamide **17p** (301 mg, 51% yield). Cleavage of the Boc group of **17p** (290 mg, 0.683 mmol) generated **18p** (205 mg, 83% yield, Purity: 99%; HPLC). ^1^H NMR (400 MHz, MeOD) δ 8.25 (s, 1H), 7.38 (d, *J* = 3.7 Hz, 1H), 6.96 (d, *J* = 3.7 Hz, 1H), 4.23 − 4.07 (m, 2H), 3.93 (ddd, *J* = 13.2, 8.8, 3.6 Hz, 2H), 3.00 (s, 2H), 1.86 (ddd, *J* = 13.3, 8.8, 4.0 Hz, 2H), 1.63 (ddd, *J* = 14.3, 7.2, 3.8 Hz, 2H), 1.12 (s, 3H); ^13^C NMR (101 MHz, MeOD) δ 152.70, 143.22, 124.96, 105.54, 103.12, 68.12, 52.78, 45.07, 34.73, 33.46, 23.56; HRMS (MM: ESI-APCI+) *m/z* calc’d for C_13_H_21_N_6_O_2_S [M + H]^+^: 325.1447; found: 325.1443.

#### 4.1.33. N-(1-(7H-Pyrrolo[2,3-d]pyrimidin-4-yl)piperidin-4-yl)methanesulfamide hydrochloride (18q)

According to step 1 of **General Procedure B1** starting from pyrrolopyrimidine **8** (150 mg, 0.977 mmol, 1.00 equiv) and amine **13q** (254 mg, 1.27 mmol, 1.30 equiv), the intermediate **14q** was obtained (230 mg, 74% yield). Removal of the Boc group of **14q** (207 mg, 0.652 mmol) under acidic condition provided amine **15q** (166 mg, 100% yield). Then, **15q** was used directly in the next step to give **17q** (20.0 mg, 8% yield). Cleavage of the Boc group of **17q** generated **18q** (15.8 mg, 94% yield, Purity: 100%; HPLC). ^1^H NMR (400 MHz, MeOD) δ 8.28 (s, 1H), 7.39 (d, *J* = 3.7 Hz, 1H), 6.95 (d, *J* = 3.7 Hz, 1H), 4.53 (d, *J* = 14.0 Hz, 2H), 3.74 − 3.60 (m, 3H), 2.31 − 2.21 (m, 2H), 1.83 − 1.68 (m, 2H); ^13^C NMR (101 MHz, DMSO-d_6_) δ 151.63, 147.14, 144.00, 123.84, 103.55, 101.38, 48.91, 45.87, 31.97; HRMS (MM: ESI-APCI+) *m/z* calc’d for C_11_H_17_N_6_O_2_S [M + H]^+^: 297.1134; found: 297.1129.

#### 4.1.34. 2-(Methylsulfonyl)-8-(7H-pyrrolo[2,3-d]pyrimidin-4-yl)-2,8-diazaspiro[4.5]decane (18r)

According to step 1 of **General Procedure B1** starting from pyrrolopyrimidine **8** (50.0 mg, 0.326 mmol, 1.00 equiv) and amine **13r** (102 mg, 0.423 mmol, 1.30 equiv), the intermediate **14r** was obtained (75.0 mg, 64% yield). Removal of the Boc group of **14r** (67.2 mg, 0.188 mmol) under acidic condition provided amine **15r** (53.6 mg, 97% yield). Then, **15r** (40.0 mg, 0.136 mmol) was used directly in the next step to give sulfamide **17r** (31.9 mg, 54% yield). Cleavage of the Boc group of **17r** (30.0 mg, 0.0687 mmol) generated **18r** (18.8 mg, 73% yield, Purity: 98%; HPLC). ^1^H NMR (400 MHz, MeOD) δ 8.28 (s, 1H), 7.39 (d, *J* = 3.7 Hz, 1H), 6.96 (d, *J* = 3.7 Hz, 1H), 4.15 − 3.95 (m, 4H), 3.40 (t, *J* = 7.2 Hz, 2H), 3.22 (s, 2H), 2.00 − 1.92 (m, 2H), 1.95 − 1.84 (m, 4H); ^13^C NMR (101 MHz, MeOD) δ 166.99, 147.19, 143.39, 125.04, 105.35, 103.30, 58.68, 47.55, 46.44, 42.06, 36.89, 35.56; HRMS (MM: ESI-APCI+) *m/z* calc’d for C_14_H_21_N_6_O_2_S [M + H]^+^: 337.1447; found: 337.1441.

#### 4.1.35. N-((1-(7H-Pyrrolo[2,3-d]pyrimidin-4-yl)piperidin-4-yl)methyl)thiophene-2-sulfonamide hydrochloride (20a)

The title compound was synthesised according to the **General Procedure B2** from **15c** (30.0 mg, 0.112 mmol, 1.00 equiv), thiophene-2-sulfonyl chloride (21.5 mg, 0.118 mmol, 1.05 equiv) and Et_3_N (70.0 µL, 0.504 mmol, 4.50 equiv). The product was purified by flash column chromatography (1:20 MeOH:CH_2_Cl_2_) on silica gel: 10.7 mg (25% yield, Purity: 96%; HPLC). ^1^H NMR (400 MHz, MeOD) δ 8.10 (s, 1H), 7.76 (dd, *J* = 5.0, 1.3 Hz, 1H), 7.60 (dd, *J* = 3.7, 1.3 Hz, 1H), 7.14 (dd, *J* = 5.0, 3.7 Hz, 1H), 7.11 (d, *J* = 3.7 Hz, 1H), 6.59 (d, *J* = 3.7 Hz, 1H), 4.74 (d, *J* = 13.6 Hz, 2H), 3.16 − 3.04 (m, 2H), 2.86 (d, *J* = 6.3 Hz, 2H), 1.90 − 1.77 (m, 3H), 1.30 − 1.18 (m, 2H); ^13^C NMR (101 MHz, MeOD) δ 168.08, 156.85, 150.94, 150.21, 141.67, 131.45, 131.32, 127.06, 120.85, 102.79, 101.16, 45.71, 36.43, 29.48; HRMS (MM: ESI-APCI+) *m/z* calc’d for C_16_H_20_N_5_O_2_S_2_ [M + H]^+^: 378.1058; found: 378.1053.

#### 4.1.36. N-((1-(7H-Pyrrolo[2,3-d]pyrimidin-4-yl)piperidin-4-yl)methyl)-4-methylbenzenesulfonamide hydrochloride (20b)

The title compound was synthesised according to the **General Procedure B2** from **15c** (35.0 mg, 0.131 mmol, 1.00 equiv), 4-methylbenzenesulfonyl chloride (26.2 mg, 0.137 mmol, 1.05 equiv) and Et_3_N (82.0 µL, 0.588 mmol, 4.50 equiv). The product was purified by flash column chromatography (1:20 MeOH:CH_2_Cl_2_) on silica gel: 27.6 mg (55% yield, Purity: 99%; HPLC). ^1^H NMR (400 MHz, MeOD) δ 8.10 (s, 1H), 7.73 (d, *J* = 8.3 Hz, 2H), 7.37 (d, *J* = 8.0 Hz, 2H), 7.11 (d, *J* = 3.7 Hz, 1H), 6.58 (d, *J* = 3.7 Hz, 1H), 4.72 (d, *J* = 13.6 Hz, 2H), 3.15 − 2.97 (m, 2H), 2.75 (d, *J* = 6.4 Hz, 2H), 2.42 (s, 3H), 1.91–1.74 (m, 3H), 1.27 − 1.17 (m, 2H); ^13^C NMR (101 MHz, MeOD) δ 158.21, 152.32, 151.60, 144.58, 139.09, 130.71, 128.00, 122.23, 104.17, 102.56, 49.64, 47.09, 37.89, 30.86, 21.42; HRMS (MM: ESI-APCI+) *m/z* calc’d for C_19_H_24_N_5_O_2_S [M + H]^+^: 386.1651; found: 386.1645.

#### 4.1.37. N-((1-(7H-Pyrrolo[2,3-d]pyrimidin-4-yl)piperidin-4-yl)methyl)cyclopropanesulfonamide hydrochloride (20c)

The title compound was synthesised according to the **General Procedure B2** from **15c** (35.0 mg, 0.131 mmol, 1.00 equiv), cyclopropanesulfonyl chloride (13.9 µL, 0.138 mmol, 1.05 equiv) and Et_3_N (82.0 µL, 0.588 mmol, 4.50 equiv). The product was purified by flash column chromatography (1:20 MeOH:CH_2_Cl_2_) on silica gel: 10.1 mg (22% yield, Purity: 95%; HPLC). ^1^H NMR (400 MHz, MeOD) δ 8.11 (s, 1H), 7.12 (d, *J* = 3.7 Hz, 1H), 6.61 (d, *J* = 3.7 Hz, 1H), 4.78 (d, *J* = 13.4 Hz, 2H), 3.15 (td, *J* = 13.4, 2.3 Hz, 2H), 3.03 (d, *J* = 6.5 Hz, 2H), 2.53 (tt, *J* = 7.9, 4.9 Hz, 1H), 1.93 (d, *J* = 12.9 Hz, 2H), 1.90 − 1.80 (m, 1H), 1.31 (qd, *J* = 12.6, 3.8 Hz, 2H), 1.09 − 0.92 (m, 4H); ^13^C NMR (101 MHz, MeOD) δ 157.00, 151.07, 149.62, 122.93, 103.98, 103.25, 49.26, 47.53, 38.12, 30.76, 30.52, 5.51; HRMS (MM: ESI-APCI+) *m/z* calc’d for C_15_H_22_N_5_O_2_S [M + H]^+^: 336.1494; found: 336.1489.

#### 4.1.38. N-(4–(7H-Pyrrolo[2,3-d]pyrimidin-4-yl)phenyl)methanesulfamide hydrochloride (25a)

According to step 1 (procedure 1) of **General Procedure C** starting from pyrrolopyrimidine **8** (100 mg, 0.651 mmol, 1.00 equiv), bronic acid **21a** (185 mg, 0.782 mmol, 1.20 equiv), and Pd(PPh_3_)_4_ (75.2 mg, 0.0651 mmol, 0.10 equiv), the intermediate **22a** was obtained (137 mg, 68% yield). Removal of the Boc group of **22a** (106 mg, 0.342 mmol) under acidic condition provided amine **23a** (80 mg, 95% yield). Then, **23a** (75.5 mg, 0.306 mmol) was used directly in the next step to give sulfamide **24a** (33.1 mg, 27% yield). Cleavage of the Boc group of **24a** (31.0 mg, 0.0796 mmol) generated **25a** (21.8 mg, 84% yield, Purity: 98%; HPLC). ^1^H NMR (400 MHz, MeOD) δ 8.99 (s, 1H), 8.06 (d, *J* = 8.8 Hz, 2H), 7.94 (d, *J* = 3.7 Hz, 1H), 7.53 (d, *J* = 8.8 Hz, 2H), 7.21 (d, *J* = 3.7 Hz, 1H); ^13^C NMR (101 MHz, MeOD) δ 153.71, 151.97, 145.84, 144.92, 133.43, 131.83, 123.80, 119.21, 115.76, 104.94; HRMS (MM: ESI-APCI+) *m/z* calc’d for C_12_H_12_N_5_O_2_S [M + H]^+^: 290.0712; found: 290.0709.

#### 4.1.39. N-(4-(1H-Pyrrolo[2,3-b]pyridin-4-yl)phenyl)methanesulfamide hydrochloride (25b)

According to step 1 (procedure 1) of **General Procedure C** starting from pyrrolopyrimidine **8** (150 mg, 0.983 mmol, 1.00 equiv) and boronic acid **21b** (291 mg, 1.23 mmol, 1.25 equiv), the intermediate **22b** was obtained (131 mg, 43% yield). Removal of the Boc group of **22b** (100 mg, 0.323 mmol) under acidic condition provided amine **23b** (75 mg, 100% yield). Then, **23b** (50.0 mg, 0.203 mmol) was used directly in the next step to give sulfamide **24b** (26.0 mg, 52% yield). Cleavage of the Boc group of **24b** (20.0 mg, 0.0616 mmol) generated **25b** (9.8 mg, 49% yield, Purity: 97%; HPLC). ^1^H NMR (400 MHz, MeOD) δ 8.42 (d, *J* = 6.3 Hz, 1H), 7.88 (d, *J* = 8.5 Hz, 2H), 7.75 (d, *J* = 3.5 Hz, 1H), 7.68 (d, *J* = 6.3 Hz, 1H), 7.46 (d, *J* = 8.5 Hz, 2H), 7.06 (d, *J* = 3.5 Hz, 1H); ^13^C NMR (101 MHz, MeOD) δ 152.02, 143.51, 140.40, 134.04, 131.29, 130.88, 130.53, 124.27, 119.61, 115.59, 103.97; HRMS (MM: ESI-APCI+) *m/z* calc’d for C_13_H_13_N_4_O_2_S [M + H]^+^: 289.0759; found: 289.0757.

#### 4.1.40. N-(2-Fluoro-4-(7H-pyrrolo[2,3-d]pyrimidin-4-yl)benzyl)methanesulfamide hydrochloride (25c)

According to step 1 (**procedure 1**) of **General Procedure C** starting from pyrrolopyrimidine **8** (120 mg, 0.781 mmol, 1.00 equiv), boronic acid **21c** (252 mg, 0.938 mmol, 1.20 equiv) and Pd(PPh_3_)_4_ (90.0 mg, 0.0781 mmol, 0.10 equiv), the intermediate **22c** was obtained (189 mg, 71% yield). Removal of the Boc group of **22c** (160 mg, 0.467 mmol) under acidic condition provided amine **23c** (120 mg, 92% yield). Then, **23c** (103 mg, 0.370 mmol) was used directly in the next step to give sulfamide **24c** (96.8 mg, 62% yield). Cleavage of the Boc group of **24c** (50.0 mg, 0.119 mmol) generated **25c** (39.6 mg, 93% yield, Purity: 100%; HPLC). ^1^H NMR (400 MHz, MeOD) δ 9.08 (s, 1H), 7.99 (d, *J* = 3.7 Hz, 1H), 7.97 − 7.87 (m, 2H), 7.84 (d, *J* = 10.4 Hz, 1H), 7.20 (d, *J* = 3.7 Hz, 1H), 4.44 (s, 2H); ^13^C NMR (101 MHz, MeOD) δ 163.39, 160.92, 154.22, 150.81, 150.79, 145.30, 134.30, 132.85, 132.73, 132.70, 132.69, 131.84, 131.75, 126.61, 126.58, 117.26, 117.02, 116.70, 104.56, 41.14, 41.09; ^19^F NMR (377 MHz, MeOD) δ −117.65; HRMS (MM: ESI-APCI+) *m/z* calc’d for C_13_H_12_FN_5_O_2_SNa [M + Na]^+^: 344.0585; found: 344.0593.

#### 4.1.41. N-(4-(7H-Pyrrolo[2,3-d]pyrimidin-4-yl)benzyl)methanesulfamide hydrochloride (25d)

According to step 1 (**procedure 1**) of **General** Procedure C starting from pyrrolopyrimidine **8** (150 mg, 0.977 mmol, 1.00 equiv), boronic acid **21d** (307 mg, 1.22 mmol, 1.25 equiv), and Pd(PPh_3_)_4_ (79.0 mg, 0.0684 mmol, 0.0700 equiv), the intermediate **22d** was obtained (176 mg, 56% yield). Removal of the Boc group of **22d** (150 mg, 0.462 mmol) under acidic condition provided amine **23d** (120 mg, 100% yield). Then, **23d** (88.9 mg, 0.341 mmol) was used directly in the next step to give sulfamide **24d** (38.9 mg, 28% yield). Cleavage of the Boc group of **24d** (20.0 mg, 0.0496 mmol) generated **25d** (6.7 mg, 40% yield, Purity: 100%; HPLC). ^1^H NMR (400 MHz, MeOD) δ 9.03 (s, 1H), 8.05 (d, *J* = 8.2 Hz, 2H), 7.93 (d, *J* = 3.7 Hz, 1H), 7.79 (d, *J* = 8.1 Hz, 2H), 7.17 (d, *J* = 3.7 Hz, 1H), 4.39 (s, 2H); ^13^C NMR (101 MHz, MeOD) δ 153.97, 152.68, 145.79, 145.66, 133.50, 130.59, 130.46, 130.17, 116.52, 104.55, 47.40; HRMS (MM: ESI-APCI+) *m/z* calc’d for C_13_H_14_N_5_O_2_S [M + H]^+^: 304.0868; found: 304.0863.

#### 4.1.42. N-(4-(7-Methyl-7H-pyrrolo[2,3-d]pyrimidin-4-yl)benzyl)methanesulfamide hydrochloride (25e)

According to step 1 (procedure 1) of **General** Procedure C starting from pyrrolopyrimidine **9a** (200 mg, 1.19 mmol, 1.00 equiv), boronic acid **21e** (375 mg, 1.49 mmol, 1.25 equiv), and Pd(PPh_3_)_4_ (96.5 mg, 0.0835 mmol, 0.0700 equiv), the intermediate **22e** was obtained (360 mg, 89% yield). Removal of the Boc group of **22e** (289 mg, 1.07 mmol) under acidic condition provided amine **23e** (289 mg, 98% yield). Then, **23e** (200 mg, 0.839 mmol) was used directly in the next step to give sulfamide **24e** (115 mg, 33% yield). Cleavage of the Boc group of **24e** generated **25e** (65.0 mg, 67% yield, Purity: 100%; HPLC). ^1^H NMR (400 MHz, MeOD) δ 9.09 (s, 1H), 8.05 (d, *J* = 8.2 Hz, 2H), 7.97 (d, *J* = 3.7 Hz, 1H), 7.80 (d, *J* = 8.1 Hz, 2H), 7.21 (d, *J* = 3.7 Hz, 1H), 4.39 (s, 2H), 4.08 (s, 3H); ^13^C NMR (101 MHz, MeOD) δ 152.70, 152.21, 146.09, 145.19, 137.92, 130.65, 130.24, 129.79, 116.67, 104.23, 47.38, 32.41; HRMS (MM: ESI-APCI+) *m/z* calc’d for C_14_H_16_N_5_O_2_S [M + H]^+^: 318.1025; found: 318.1019.

#### 4.1.43. N-(4-(1H-Pyrrolo[2,3-b]pyridin-4-yl)benzyl)methanesulfamide hydrochloride (25f)

According to step 1 (**procedure 2**) of **General** Procedure C starting from pyrrolopyrimidine **8** (50.0 mg, 0.328 mmol, 1.00 equiv), boronic acid **21f** (86.4 mg, 0.344 mmol, 1.05 equiv) and PdCl_2_(dppf) (24.0 mg, 0.0328 mmol, 0.100 equiv), the intermediate **22f** was obtained (78.0 mg, 74% yield). Removal of the Boc group of **22f** (47.6 mg, 0.147 mmol) under acidic condition provided amine **23f** (36.6 mg, 96% yield). Then, **23f** (22.7 mg, 0.0874 mmol) was used directly in the next step to give sulfamide **24f** (8.6 mg, 25% yield). Cleavage of the Boc group of **24f** generated **25f** (6.5 mg, 86% yield, Purity: 98%; HPLC). ^1^H NMR (400 MHz, MeOD) δ 8.44 (d, *J* = 6.2 Hz, 1H), 7.88 (d, *J* = 8.2 Hz, 2H), 7.75 (d, *J* = 3.6 Hz, 1H), 7.72 − 7.65 (m, 3H), 7.02 (d, *J* = 3.6 Hz, 1H), 4.35 (s, 2H); ^13^C NMR (101 MHz, MeOD) δ 151.39, 142.86, 141.38, 136.45, 135.08, 130.54, 130.24, 129.88, 124.39, 116.03, 103.60, 47.52; HRMS (MM: ESI-APCI+) *m/z* calc’d for C_14_H_15_N_4_O_2_S [M + H]^+^: 303.0916; found: 303.0910.

#### 4.1.44. N-(3-(7H-Pyrrolo[2,3-d]pyrimidin-4-yl)benzyl)methanesulfamide hydrochloride (25g)

According to step 1 (procedure 1) of **General** Procedure C starting from pyrrolopyrimidine **8** (100 mg, 0.651 mmol, 1.00 equiv) boronic acid **21g** (204 mg, 0.814 mmol, 1.25 equiv), and Pd(PPh_3_)_4_ (53.0 mg, 0.0456 mmol, 0.0700 equiv), the intermediate **22g** was obtained (114 mg, 54% yield). Removal of the Boc group of **22g** (100 mg, 0.308 mmol) under acidic condition provided amine **23g** (73.0 mg, 90% yield). Then, **23g** (49.0 mg, 0.188 mmol) was used directly in the next step to give sulfamide **24g** (20.0 mg, 26% yield). Cleavage of the Boc group of **24g** generated **25g** (13.4 mg, 80% yield, Purity: 99%; HPLC). ^1^H NMR (400 MHz, MeOD) δ 9.07 (s, 1H), 8.14 (s, 1H), 8.00 − 7.94 (m, 2H), 7.81 (d, *J* = 7.7 Hz, 1H), 7.74 (t, *J* = 7.7 Hz, 1H), 7.25 (d, *J* = 3.7 Hz, 1H), 4.41 (s, 2H); ^13^C NMR (101 MHz, MeOD) δ 154.05, 152.32, 145.14, 142.41, 134.00, 133.66, 131.16, 131.13, 129.85, 129.24, 116.66, 104.96, 47.30; HRMS (MM: ESI-APCI+) *m/z* calc’d for C_13_H_14_N_5_O_2_S [M + H]^+^: 304.0868; found: 304.0863.

#### 4.1.45. N-(2–(4-(7H-Pyrrolo[2,3-d]pyrimidin-4-yl)phenyl)propan-2-yl)methanesulfamide hydrochloride (25h)

Following step 1 (procedure 1) of **General** Procedure C starting from pyrrolopyrimidine **8** (80.0 mg, 0.521 mmol, 1.00 equiv), boronic acid **21h** (175 mg, 0.625 mmol, 1.20 equiv) and Pd(PPh_3_)_4_ (60.0 mg, 0.052 mmol, 0.100 equiv), the residue of step 1 was purified by flash column chromatography (1:20 MeOH:CH_2_Cl_2_) on silica gel. The collected fraction was then used directly in the next step. Removal of the Boc group of **22h** (183 mg, 0.521 mmol) under acidic condition provided amine **23h** (138 mg, 77% yield). Then, **23h** (60.0 mg, 0.208 mmol) was used directly in the next step to give sulfamide **24h** (27.8 mg, 31% yield). Cleavage of the Boc group of **24h** (19.7 mg, 0.0457 mmol) generated **25h** (10.4 mg, 62% yield, Purity: 96%; HPLC). ^1^H NMR (400 MHz, MeOD) δ 9.05 (s, 1H), 8.04 (d, *J* = 8.4 Hz, 2H), 7.98 − 7.92 (m, 3H), 7.21 (d, *J* = 3.6 Hz, 1H), 1.78 (s, 6H); ^13^C NMR (101 MHz, MeOD) δ 155.37, 153.94, 152.36, 145.21, 133.73, 130.31, 129.15, 128.45, 116.41, 104.82, 58.80, 30.05; HRMS (MM: ESI-APCI+) *m/z* calc’d for C_15_H_18_N_5_O_2_S [M + H]^+^: 332.1181; found: 332.1176.

#### 4.1.46. N-(2–(4-(7-(Pyridin-3-yl)-7H-pyrrolo[2,3-d]pyrimidin-4-yl)phenyl)propan-2-yl)methanesulfamide hydrochloride (25i)

According to step 1 (procedure 1) of **General Procedure C** starting from pyrrolopyrimidine **9h** (60.0 mg, 0.261 mmol, 1.00 equiv), boronic acid **21i** (87.0 mg, 0.313 mmol, 1.20 equiv) and Pd(PPh_3_)_4_ (30.1 mg, 0.0261 mmol, 0.10 equiv), the intermediate **22i** was obtained (85.7 mg, 77% yield). Removal of the Boc group of **22i** (74.5 mg, 0.173 mmol) under acidic condition provided amine **23i** (63.0 mg, 100% yield). Then, **23i** (50.0 mg, 0.137 mmol) was used directly in the next step to give sulfamide **24i** (30.7 mg, 44% yield). Cleavage of the Boc group of **24i** (19.5 mg, 0.0383 mmol) generated **25i** (17.0 mg, 100% yield, Purity: 98%; HPLC). ^1^H NMR (600 MHz, MeOD) δ 9.14 (s, 1H), 8.91 (s, 1H), 8.61 (d, *J* = 4.2 Hz, 1H), 8.45 − 8.33 (m, 1H), 8.09 (d, *J* = 8.4 Hz, 2H), 7.98 (d, *J* = 3.8 Hz, 1H), 7.82 (d, *J* = 8.5 Hz, 2H), 7.68 (dd, *J* = 8.2, 4.9 Hz, 1H), 7.17 (d, *J* = 3.8 Hz, 1H), 1.79 (s, 6H); ^13^C NMR (201 MHz, MeOD) δ 159.48, 153.05, 152.93, 151.94, 148.54, 145.66, 136.79, 136.07, 133.40, 130.35, 129.82, 127.45, 125.76, 118.12, 104.21, 58.78, 30.07, 24.79, 13.92; HRMS (MM: ESI-APCI+) *m/z* calc’d for C_20_H_21_N_6_O_2_S [M + H]^+^: 409.1447; found: 409.1443.

#### 4.1.47. N-(1–(4-(7H-Pyrrolo[2,3-d]pyrimidin-4-yl)phenyl)ethyl)methanesulfamide hydrochloride (25j)

According to step 1 (procedure 1) of **General** Procedure C starting from pyrrolopyrimidine **8** (95.0 mg, 0.619 mmol, 1.00 equiv), boronic acid **21j** (197 mg, 0.742 mmol, 1.20 equiv) and Pd(PPh_3_)_4_ (71.5 mg, 0.0619 mmol, 0.10 equiv), the intermediate **22j** was obtained (84.0 mg, 40% yield). Removal of the Boc group of **22j** (58.0 mg, 0.171 mmol) under acidic condition provided amine **23j** (37.0 mg, 76% yield). Then, **23j** (27.0 mg, 0.0983 mmol) was used directly in the next step to give sulfamide **24j** (16.0 mg, 39% yield). Cleavage of the Boc group of **24j** (14.6 mg, 0.0350 mmol) generated **25j** (9.3 mg, 75% yield, Purity: 98%; HPLC). ^1^H NMR (400 MHz, MeOD) δ 9.04 (s, 1H), 8.04 (d, *J* = 8.2 Hz, 2H), 7.95 (d, *J* = 3.5 Hz, 1H), 7.80 (d, *J* = 8.2 Hz, 2H), 7.19 (d, *J* = 3.6 Hz, 1H), 4.72 (q, *J* = 6.9 Hz, 1H), 1.57 (d, *J* = 6.9 Hz, 3H); ^13^C NMR (101 MHz, MeOD) δ 153.95, 152.22, 151.97, 145.12, 133.88, 130.69, 129.52, 129.04, 116.42, 104.82, 54.21, 23.83; HRMS (MM: ESI-APCI+) *m/z* calc’d for C_14_H_16_N_5_O_2_S [M + H]^+^: 318.1025; found: 318.1021.

### 4.2. Docking

The examined **18p** was drawn using ChemDraw and converted to 3D-structure via openbabel 3.1.0^15^. Molecular docking was conducted through ICM version 3.9-2d based on Monte Carlo simulations to search the globally minimum binding pose between our compound and a target protein^16^. The co-crystal complex of ENPP1 and IJE (PDB-ID: 6 × kd)^6^a was downloaded from the Protein Data Bank^17^. The centre of IJE with the coordinates of −4.04(x), 27.29(y), 40.16(z) was defined as the centre of grid box with its width of 20 Å in each x, y, z direction respectively. Then, IJE, cofactor, and water molecules were extracted from the complex. Grid maps for five different energy terms were generated with a spacing of 0.5 Å between the grid points. Docking simulation was performed with an effort parameter of 10 during 10 iterative runs, and all the conformations with different binding affinity were collected and ranked from the dock score of ICM-Pro. The pose with the best binding energy was analysed about its intermolecular interactions by virtue of LigPlot + and PLIP^18^ and visualised using PYMOL^19^.

### 4.3. Biology

#### 4.3.1. *In vitro* enzyme assay protocol

ENPP1 hydrolyses nucleotides or nucleotide derivatives and produces nucleoside-5′-monophosphates and pyrophosphates. ENPP1 also hydrolyses 2′3′-cGAMP and produces 5′-adenosine monophosphate (AMP) and 5′ guanosine monophosphate (GMP). The produced AMP from the reaction is detected using the AMP-Glo^®^ kit (Promega). The assay kit consists of two reagents; one to terminate the AMP-generating enzymatic reaction, remove ATP and convert AMP produced into ADP, and a second reagent to convert ADP to ATP, which is used to generate a luminescence in a luciferase reaction. The amount of light measured is proportional to the amount of AMP produced by ENPP1.

The final assay reaction mixture contains a buffer of 50 mM Tris (pH 8.5), 250 mM NaCl, 0.5 mM CaCl_2_, 1 μM ZnCl_2_, 5% glycerol and 1% DMSO. Serially diluted ENPP1 inhibitors (usually range from 10 μM to 0.5 nM) are pre-incubated with human recombinant ENPP1 enzyme (R&D systems) at 3 ng/reaction for 5–10 min at room temperature (RT). The reaction is initiated by adding cGAMP (at final concentration of 5 μM) and incubating for 90 min at 37 °C. At the end of incubation, the reaction is stopped by adding 10 μL of AMP-Glo reagent I and followed by incubation at RT for one hour. After incubation, 20 μL of AMP detection solution (mixture of AMP-Glo II reagent and Kinase-Glo one at 1:100 ratio) is added and incubated at RT for one hour. The luminescence signal generated is measured using the ClarioStar plate reader (BMG Labtech). Maximal activity control (containing enzyme and substrate in presence of 1% DMSO only; MAX) and background control (containing substrate and 1% DMSO; MIN) are evaluated simultaneously. In each experiment, serially diluted reference ENPP1 inhibitor is tested together. The IC_50_ values for % remaining activity versus compound concentration are determined by fitting the inhibition curves using a three-parameter variable method in GraphPad Prism^®^ software. Triplicate wells are run at each inhibitor concentration and averaged IC_50_ value for each compound is calculated.

#### 4.3.2. Cell based assay for IRF activation in THP-1 dual cells

THP-1 dual cells were purchased from Invivogen and IRF-Lucia luciferase reporter assay was performed by manufacturer’s instruction. Briefly, cells were plated in medium in 384-well plate. Individual compounds were pre-treated with an indicated concentration before cGAMP activation, then cGAMP was added at an indicated concentration. After 24 h of incubation, supernatant was analysed using QUANTI-Luc^TM^ assay kit by measuring the relative light units of luciferase signal (RU) in a microplate reader (TECAN).

#### 4.3.3. Cell viability assay

Cell viability assay was performed using residual cells after luciferase assay. Cells were analysed using CellTiter 96 Aqueous One Solution Proliferation Assay (MTS, Promega) according to the manufactural protocols. Absorbance signal were measured using microplate reader (TECAN). Results were normalized using DMSO as a control.

#### 4.3.4. Gene expression analysis by rtPCR

Cells were harvest and RNA were isolated using NucleoSpin RNA plus (MN) according to the manufacturer’s instructions. Genomic DNA was removed and cDNA was synthesised using an iScript gDNA clear cDNA synthesis kit (Bio-Rad). Expression levels were assessed by real-time PCR (CFX96 Real-Time PCR detection system, Bio-rad) using IQ SYBR green Supermix (Bio-Rad). The mRNA levels were normalized by GAPDH and calculated by comparative Ct method.

#### 4.3.5. Enzyme-linked immunosorbent assay (ELISA)

IFN-*β* cytokine secretion were quantified by using ELISA kits from R&D Systems. THP-1 cells were seeded in 96 well plate and treated with the compounds for 8 h incubation. 100 μL of cell culture supernatant was harvested and analysed using manufactural protocols.

#### 4.3.6. Western Blot analysis

Cells were washed by PBS and harvested with RIPA buffer (Bioseasang). After centrifugation with 20,000 × g for 20 min at 4 °C. Cell lysates were separated by SDS-polyacrylamide gel electrophoresis and transferred on PVDF membranes through the Trans-Blot Turbo Transfer System (Bio-Rad). The membranes were blocked in 5% BSA in TBST for 1 h at room temperature and followed by incubation with primary antibody solution for overnight. All the antibodies were used in TBST and dilution for each antibody as follows: anti-pIRF3 (Cell Signalling/no. 4947, 1:1000), anti-pSTAT (Cell Signalling/no. 9167, 1:1000), and anti-*β*-tubulin (Cell signalling/no. 2146, 1:1000). After washing with TBST, all antibodies were detected by anti-rabbit-HRP-linked secondary antibody (Cell signalling/no. 7074, 1:3000). *β*-Tubulin was used as a loading control for the amount of total protein. Luminescence signal was detected using ECL reagent and immunoreactive bands were captured with the ChemiDoc imaging systems (Bio-Rad).

#### 4.3.7. *In vivo* efficacy

The study for murine 4T1 colon carcinoma was approved by the Institutional Animal Care and Use Committee (IACUC) of the Korea Institute of Science and Technology (KIST-2021–04-048). Female BALB/c mice were 8 weeks obtained from DBL (Korea). Mice were subcutaneously inoculated with 4T1 cells (4 × 10^5^) suspended in PBS into the right flank. The individual group was classified based on the tumour size when each tumour of each mouse reached 30–100 mm^3^. Compound or vehicle were administrated by QD per oral injection in 0.5% MC/Saline (v/v) solution. Tumour volume was calculated by following formula: Tumour volume (mm^3^) = 0.5 × [length, (mm) × width, (mm)2].

## Supplementary Material

Supplemental Material
