# Supplementary material for: Identification of novel pyrrolopyrimidine and pyrrolopyridine derivatives as potent ENPP1 inhibitors
Source: J Enzyme Inhib Med Chem. 2022 Sep 7;37(1):2434–51. doi: 10.1080/14756366.2022.2119566 (PMC9467556; doi:10.1080/14756366.2022.2119566)
Supplement: Supplemental Material [file IENZ_A_2119566_SM9843.pdf]

Supporting Information for

Identification of novel pyrrolopyrimidine and pyrrolopyridine derivatives as potent ENPP1 inhibitors

Hee Jin Jeong[a,b,†], Hye Lim Lee[c,d,†], Sung Joon Kim[e,†], Jeong Hyun Jeong[c], Su Hyun Ji[a,f], Han Byeol Kim[a,f], Miso Kang[c,d], Hwan Won Chung[g], Chan Sun Park[e], Hyunah Choo[c], Hyo Jae Yoon[b], Nam-Jung Kim[d,h], Duck-Hyung Lee[f], Sanghee Lee*[c,i], Seo-Jung Han*[a,j]

[a]*Chemical & Biological Integrative Research Center, Korea Institute of Science and Technology, 5 Hwarang-ro 14-gil, Seongbuk-gu, Seoul 02792, Republic of Korea*

[b]*Department of Chemistry, Korea University, 145 Anam-ro, Seongbuk-gu, Seoul 02841, Republic of Korea*

[c]*Brain Science Institute, Korea Institute of Science and Technology, 5 Hwarang-ro 14-gil, Seoungbuk-gu, Seoul 02792, Republic of Korea*

[d]*Department of Basic Pharmaceutical Science, College of Pharmacy, Kyung Hee University, Seoul 02447, Republic of Korea*

[e]*TXINNO Bioscience INC, 338 Gwanggyojungang-ro, Suji-gu, Yongin-si, Gyeonggi-do 16942, Republic of Korea*

[f]*Department of Chemistry, Sogang University, 35 Baekbeom Ro, Seoul 04107, Republic of Korea*

[g]*Computational Science Research Center, Korea Institute of Science and Technology, 5 Hwarang-ro 14-gil, Seongbuk-gu, Seoul 02792, Republic of Korea*

[h]*Department of Life and Nanopharmaceutical Sciences, Graduate School, Kyung Hee University, Seoul 02447, Republic of Korea*

[i]*Department for HY-KIST Bio-convergence, Hanyang University, Seoul 04763, Republic of Korea*

[j]*Division of Bio-Medical Science & Technology, KIST School, University of Science and Technology, Seoul, 02792 Republic of Korea*

[†]Hee Jin Jeong, Hye Lim Lee, and Sung Joon Kim contributed equally to this work.

Table of Contents:

^{1}H NMR, ^{13}C NMR, and ^{19}F NMR spectra ·· **S-2**
HPLC traces of **18**, **20** and **25** ·· **S-68**

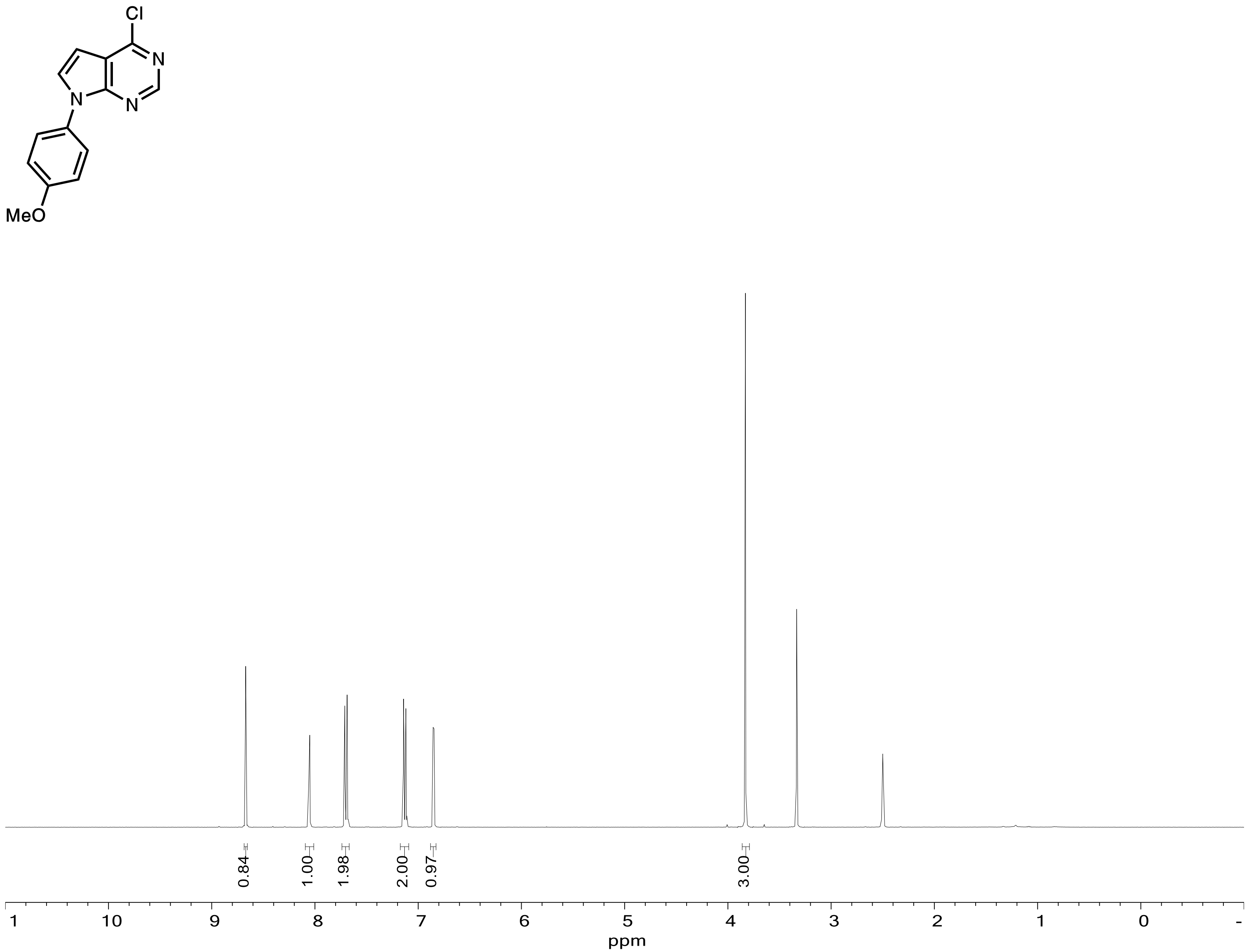

^{1}H NMR (400 MHz, DMSO-d_6) of compound **9f**.

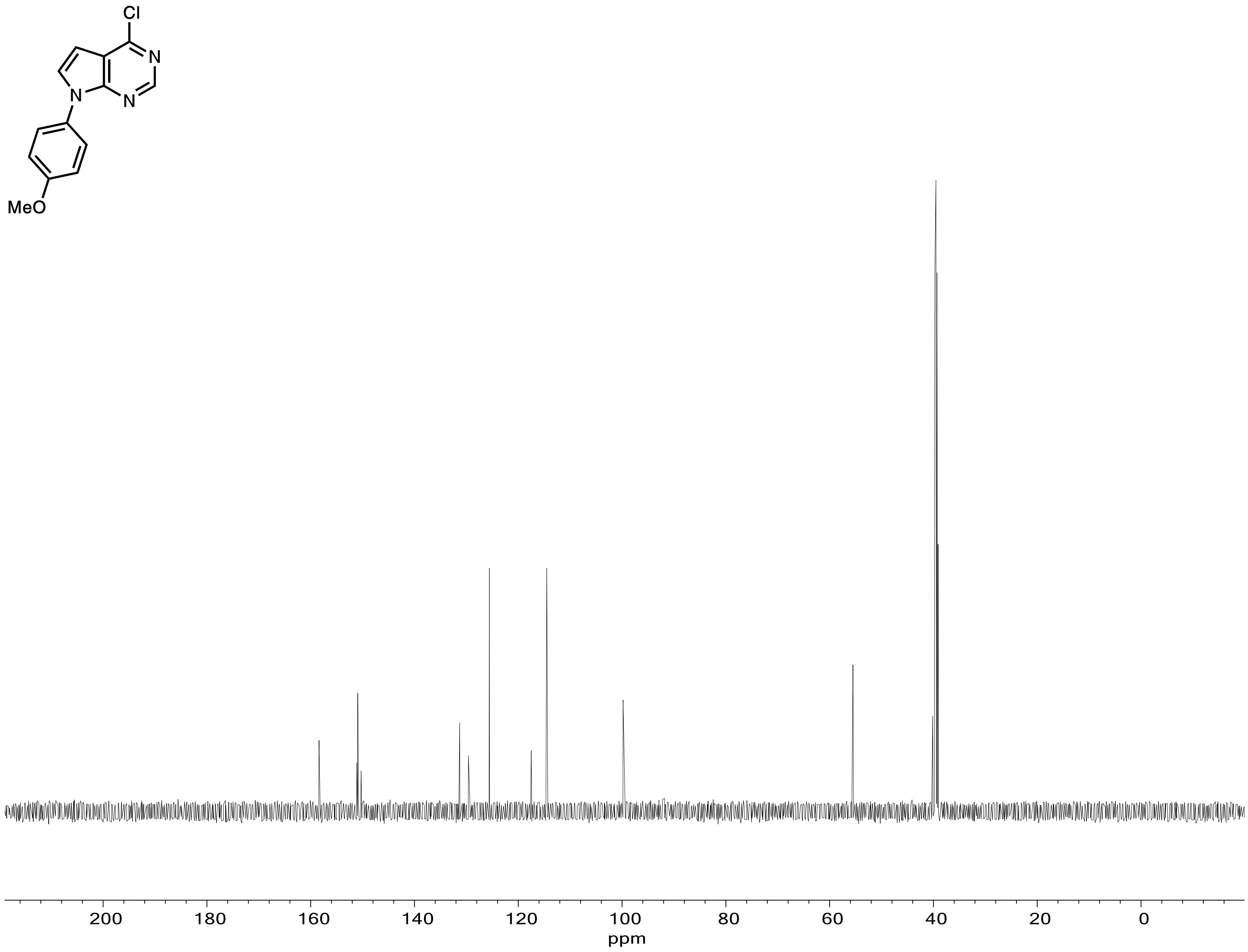

^{13}C NMR (101 MHz, DMSO-d_6) of compound **9f**.

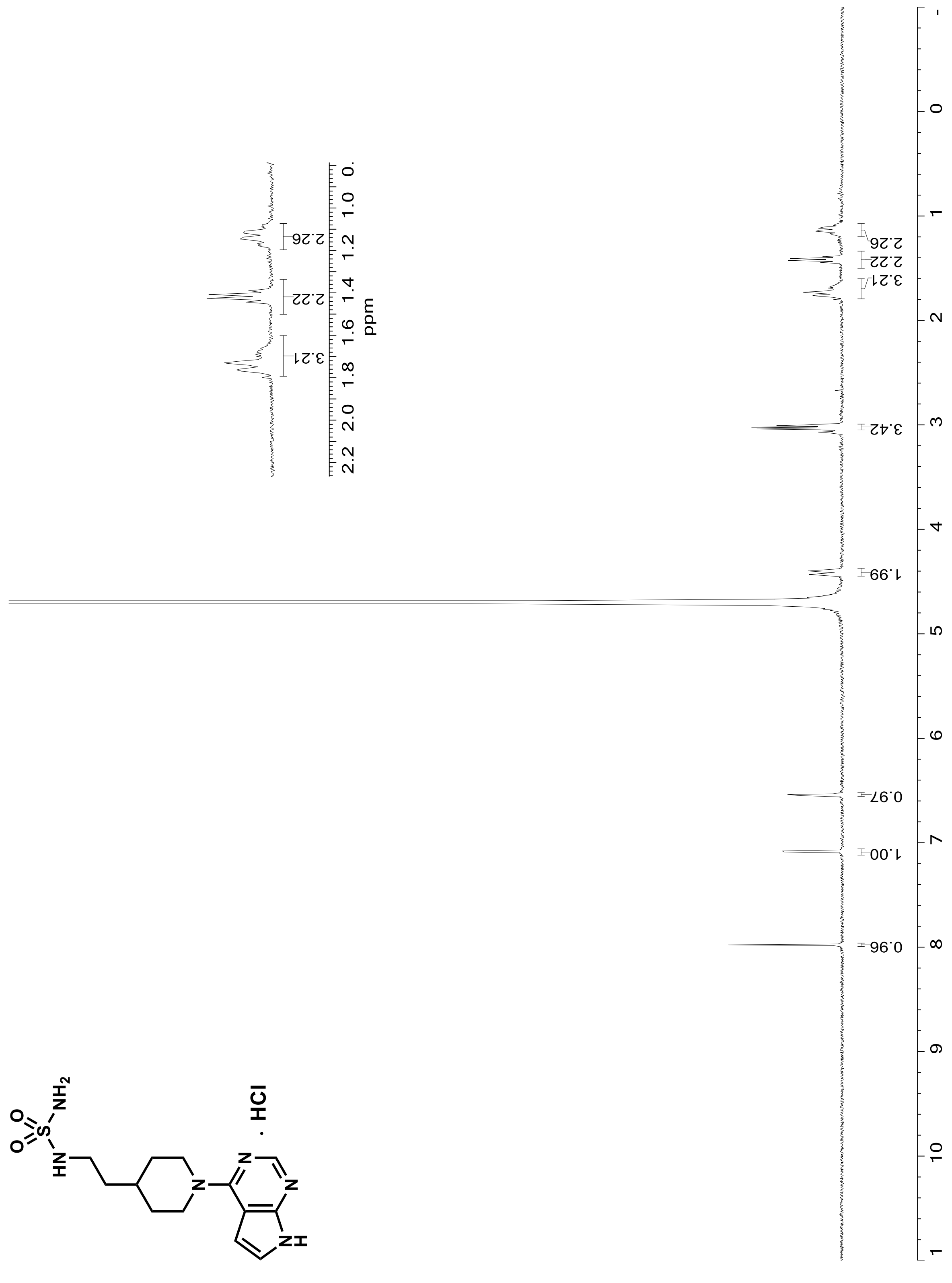

^{1}H NMR (400 MHz, D_2O) of compound **18a**.

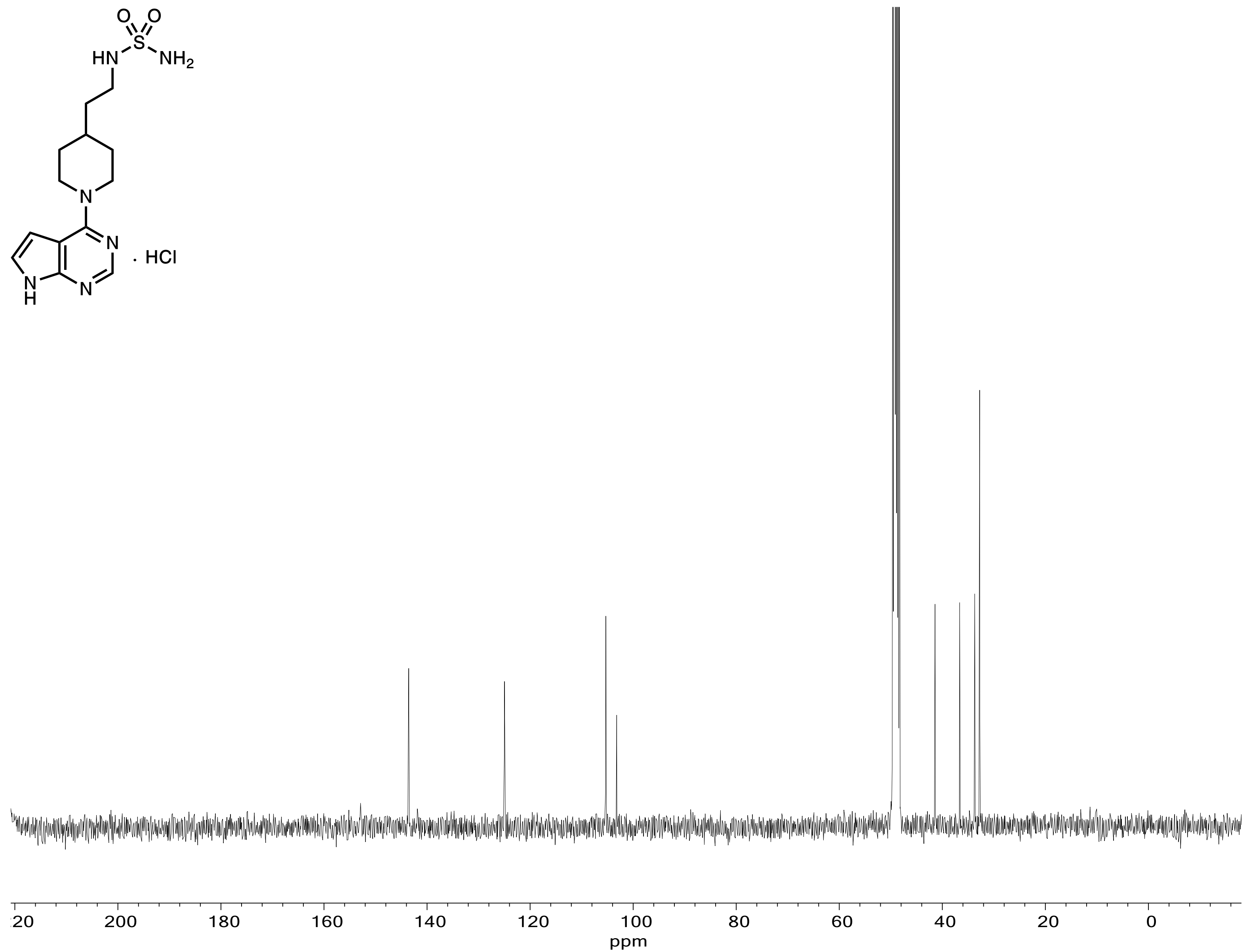

^{13}C NMR (101 MHz, MeOD) of compound **18a**.

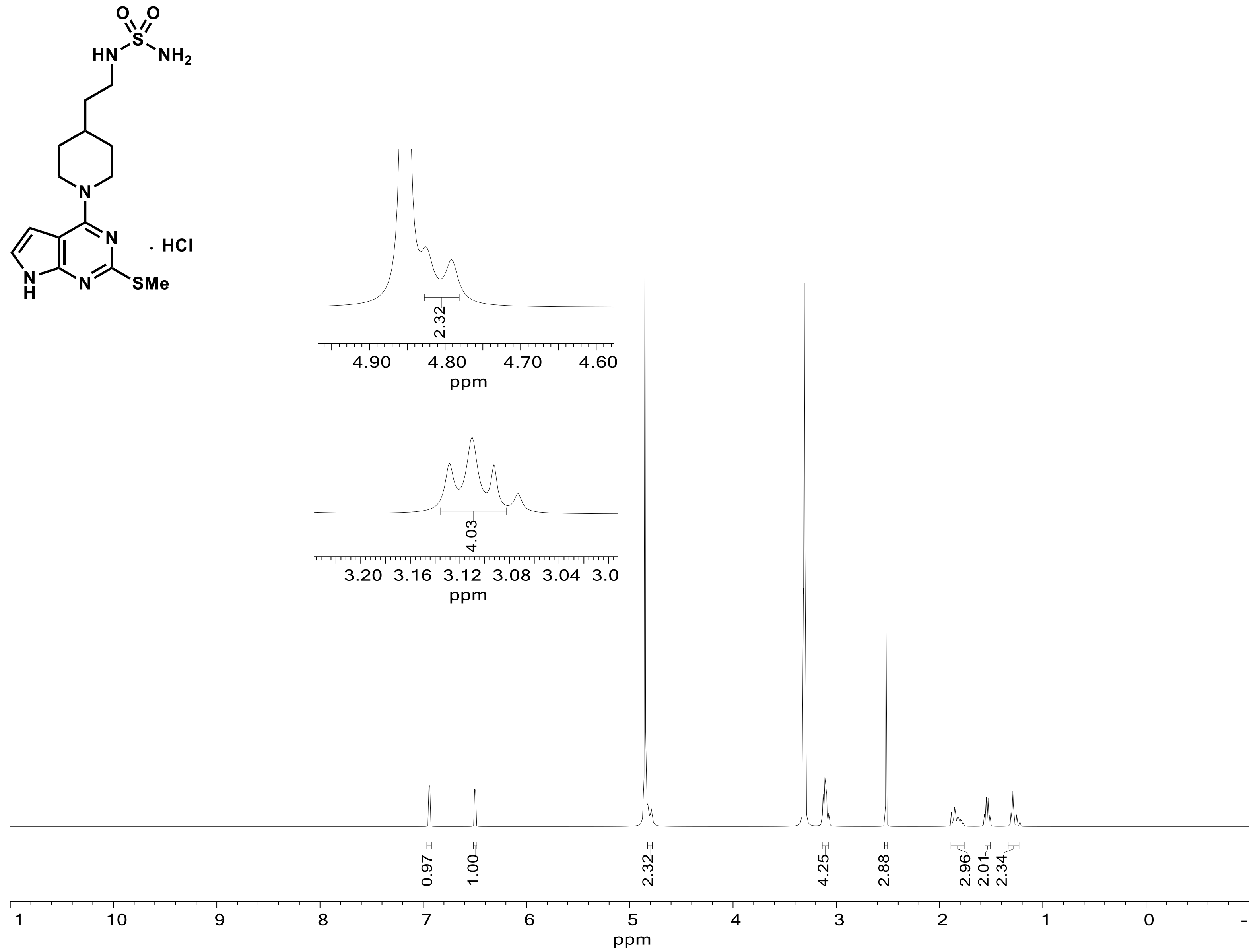

^{1}H NMR (400 MHz, MeOD) of compound **18b**.

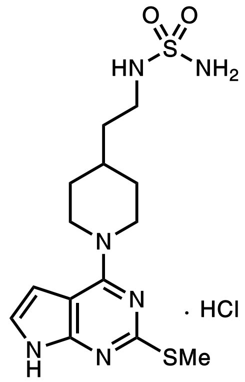

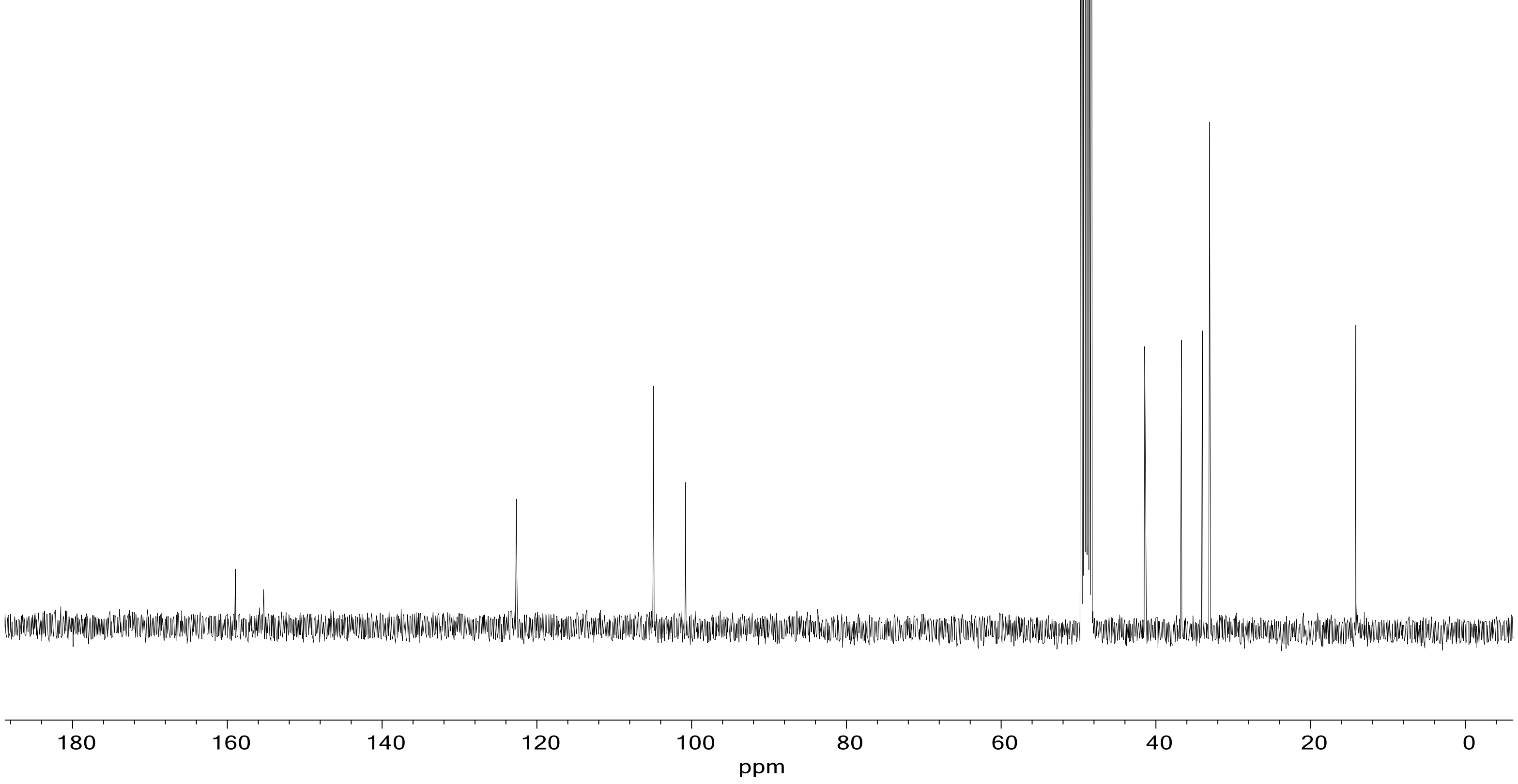

^{13}C NMR (101 MHz, MeOD) of compound **18b**.

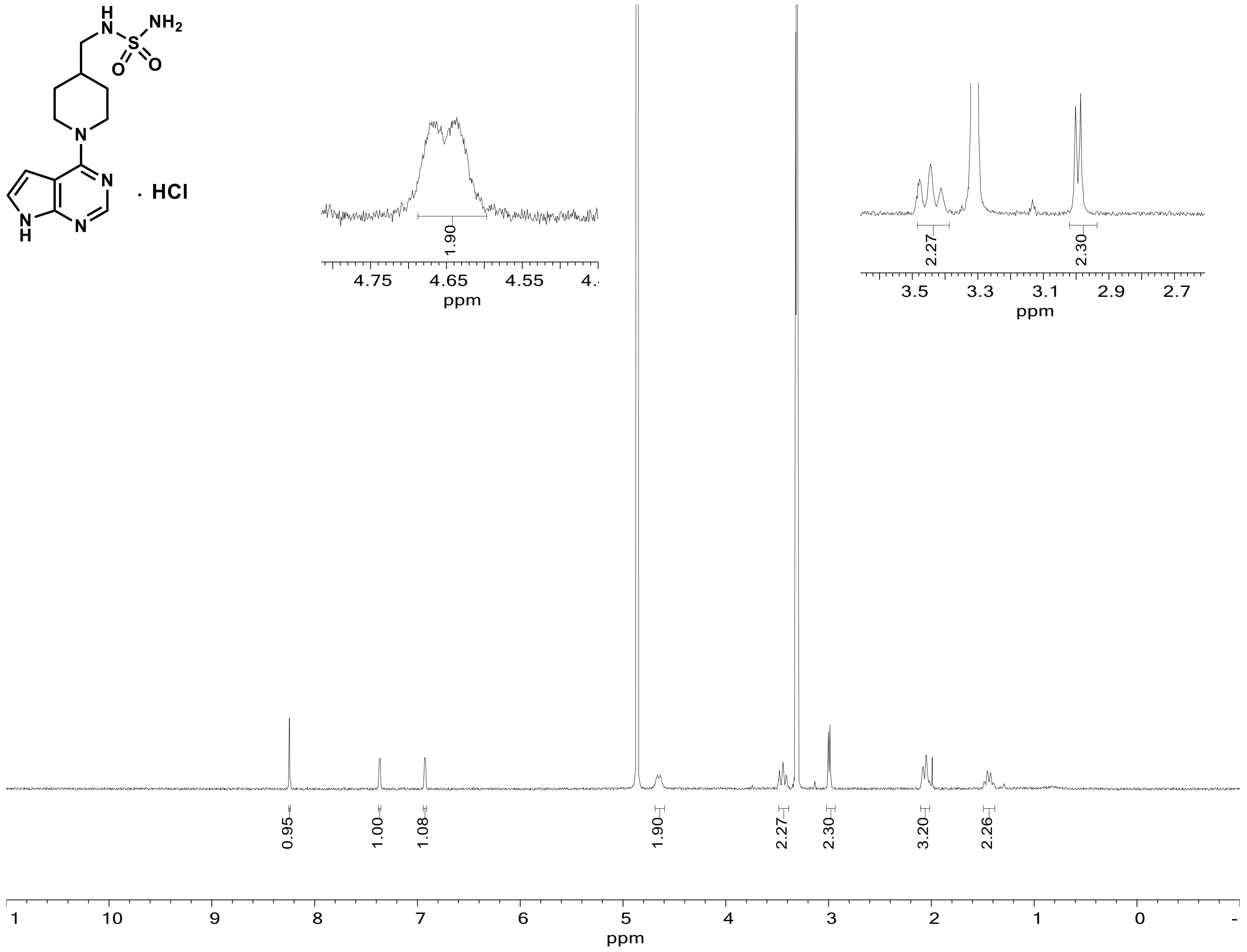

^{1}H NMR (400 MHz, MeOD) of compound **18c**.

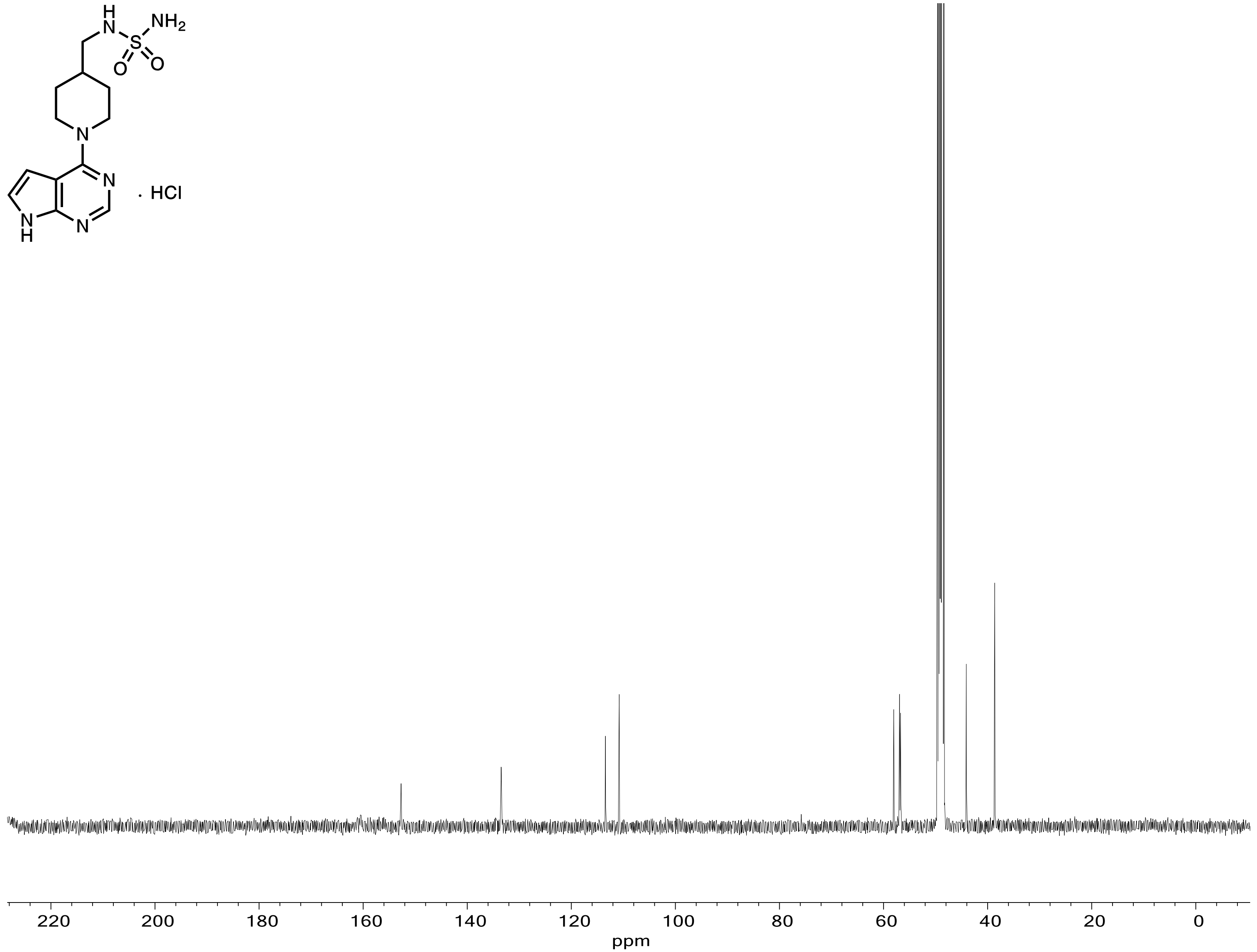

^{13}C NMR (101 MHz, DMSO-d_6) of compound **18c**.

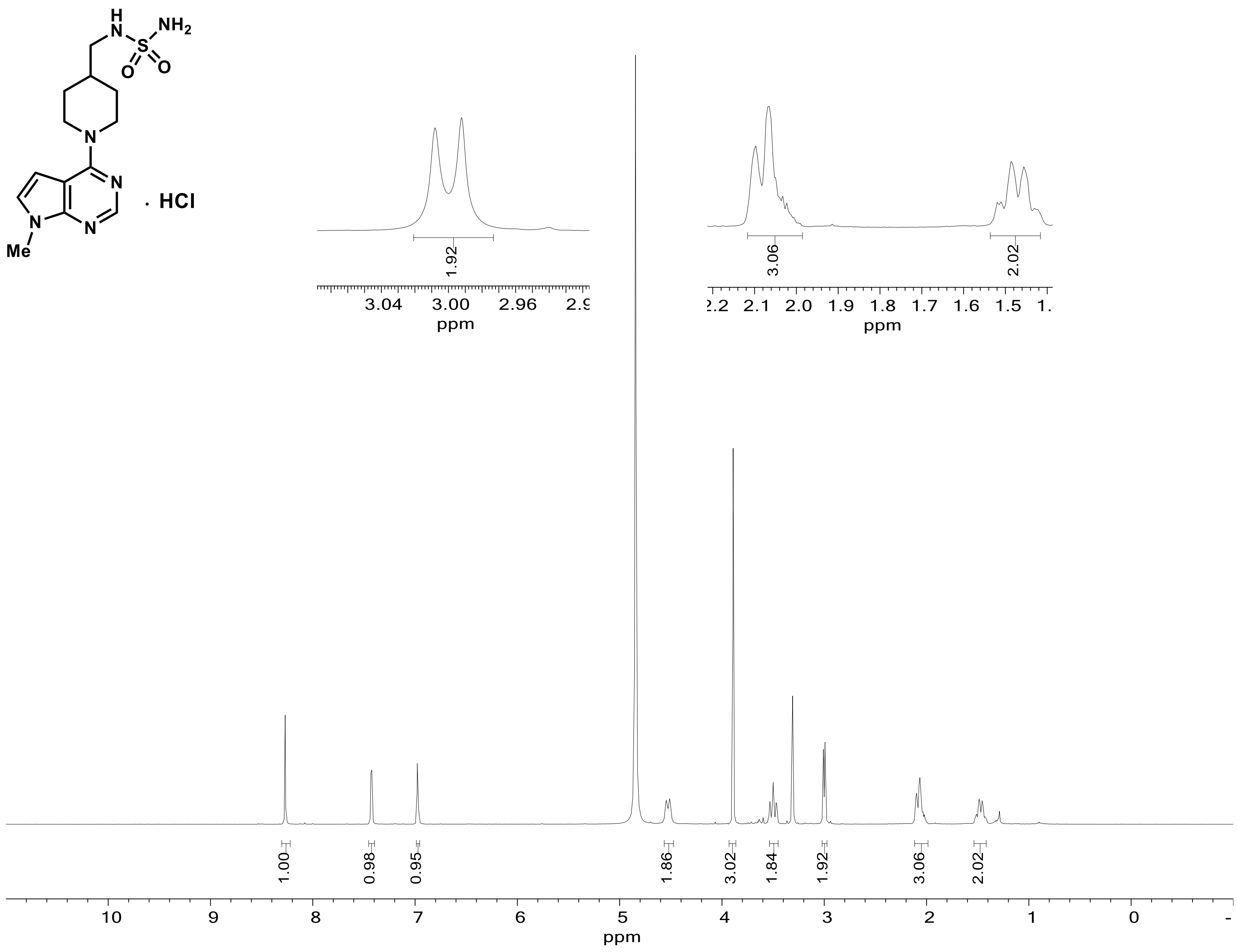

^{1}H NMR (400 MHz, MeOD) of compound **18d**.

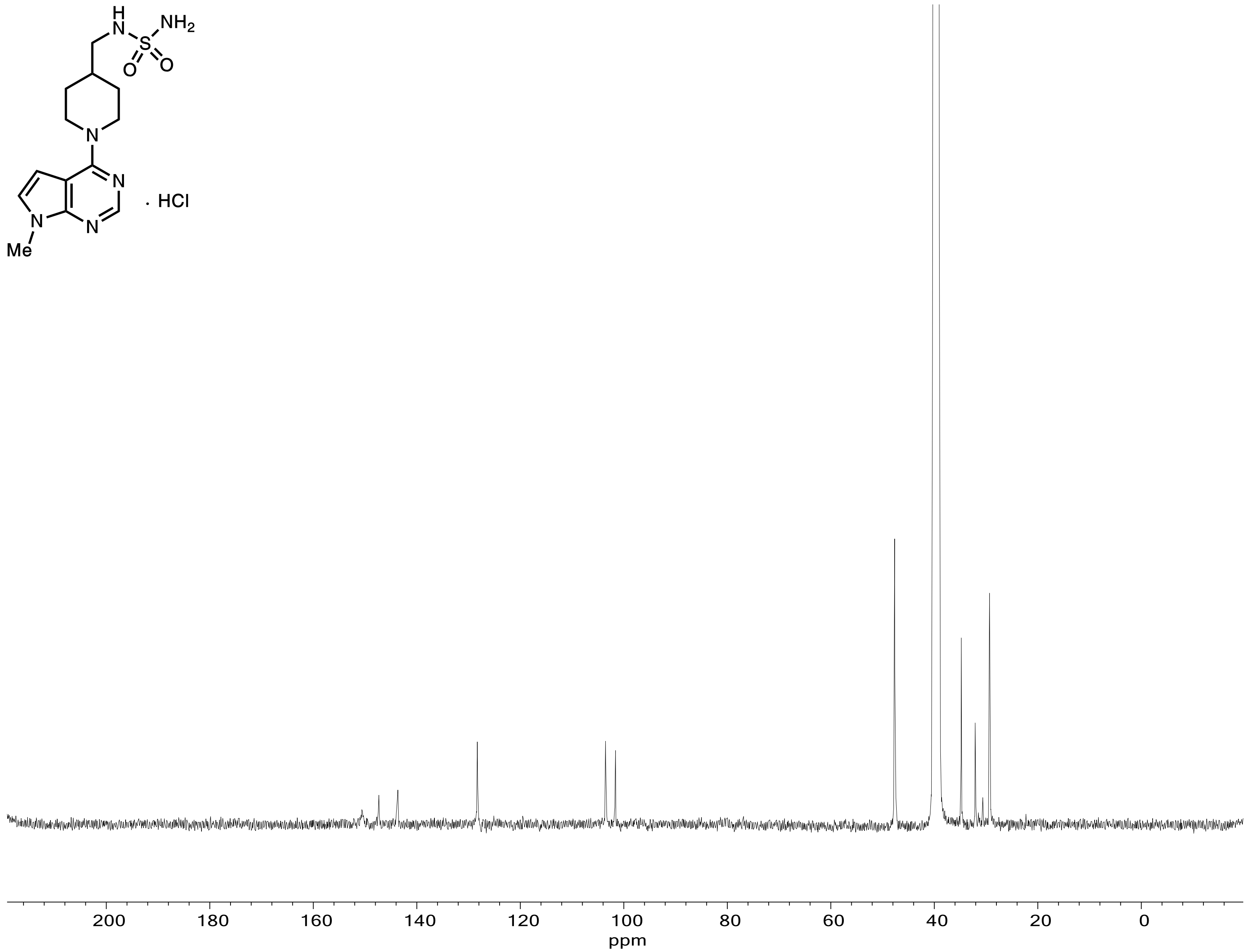

^{13}C NMR (101 MHz, DMSO-d_6) of compound **18d**.

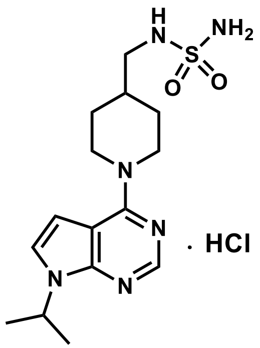

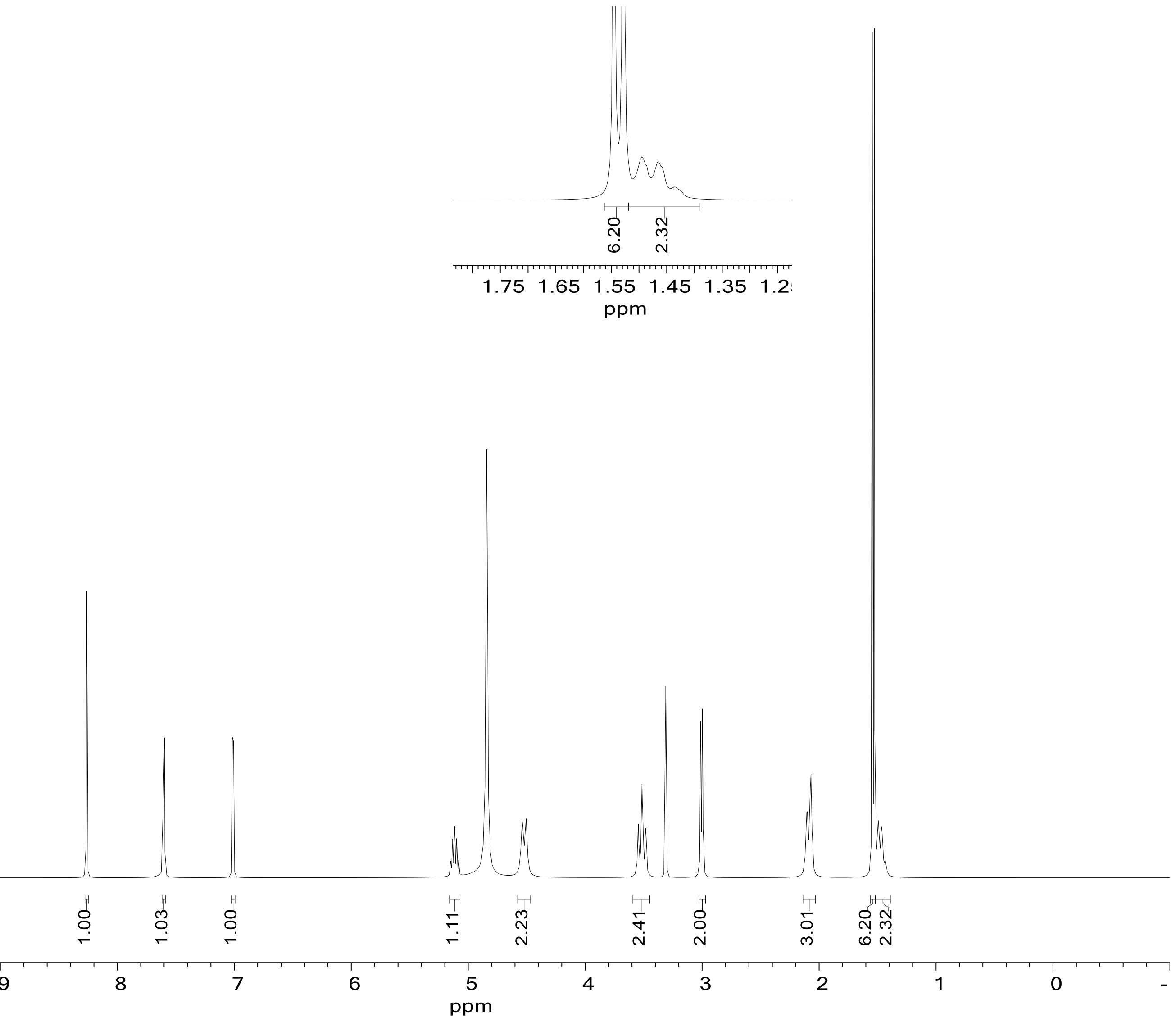

^1H NMR (400 MHz, MeOD) of compound **18e**.

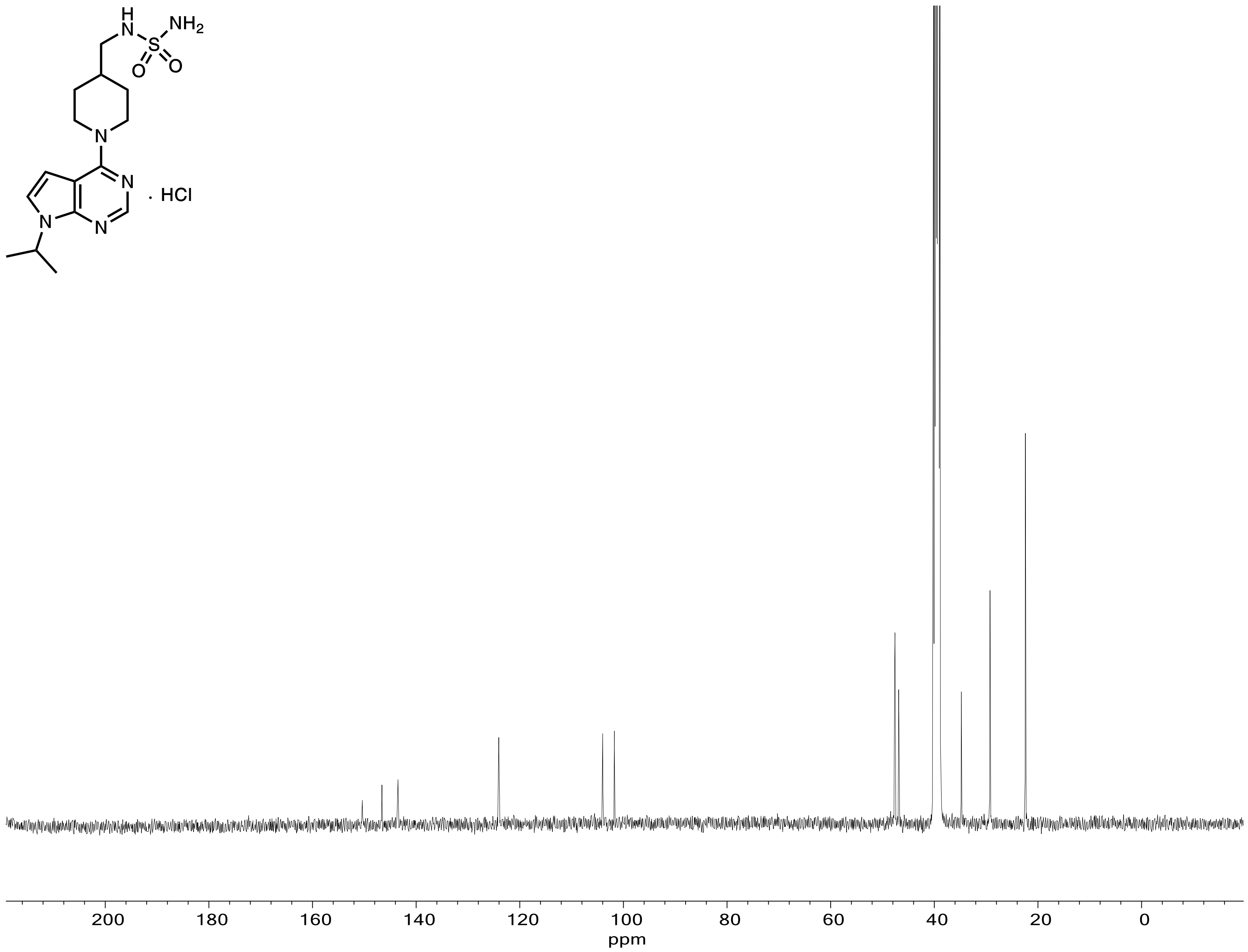

^{13}C NMR (101 MHz, DMSO-d_6) of compound **18e**.

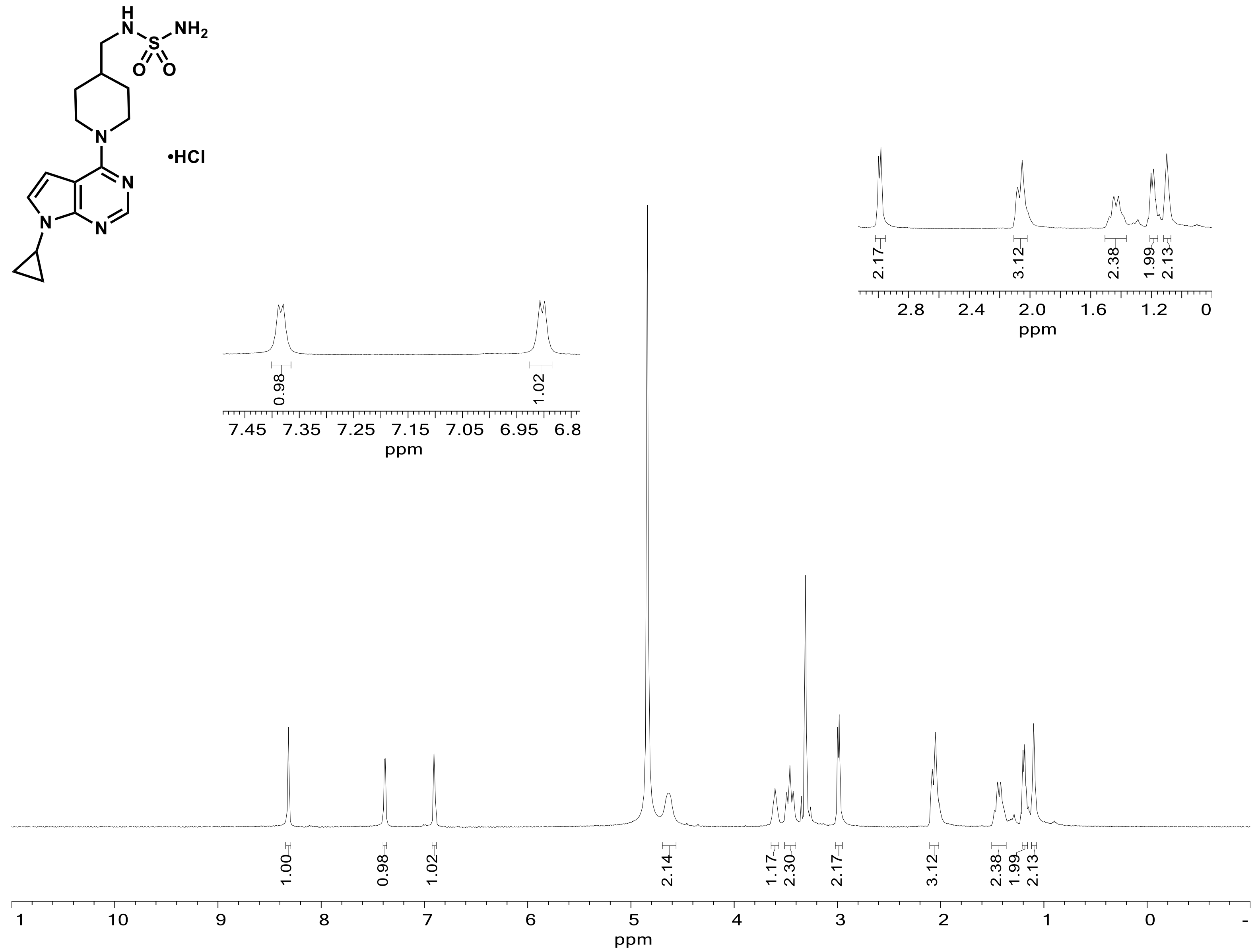

^1H NMR (400 MHz, MeOD) of compound **18f**.

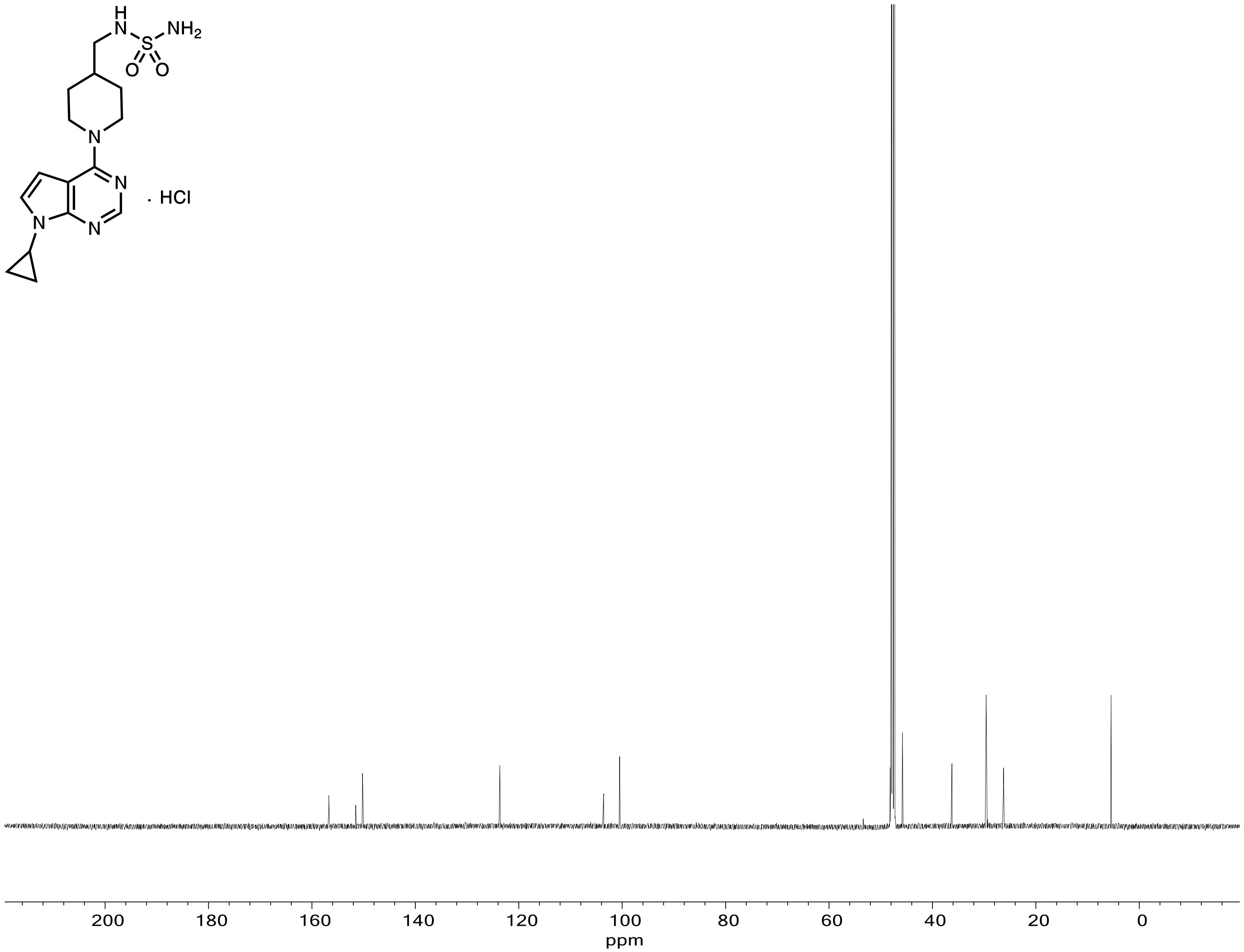

^{13}C NMR (101 MHz, MeOD) of compound **18f**.

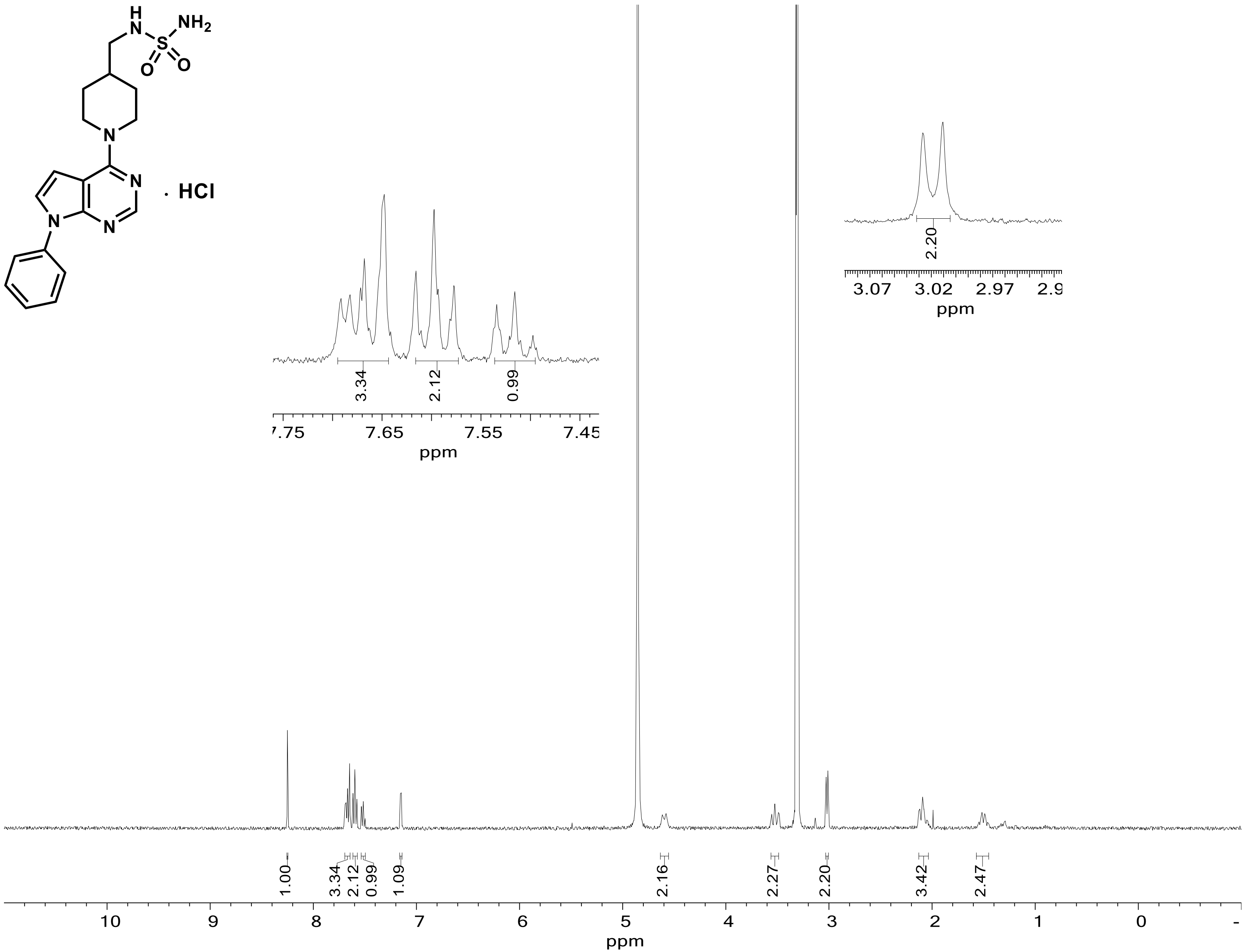

^{1}H NMR (400 MHz, MeOD) of compound **18g**.

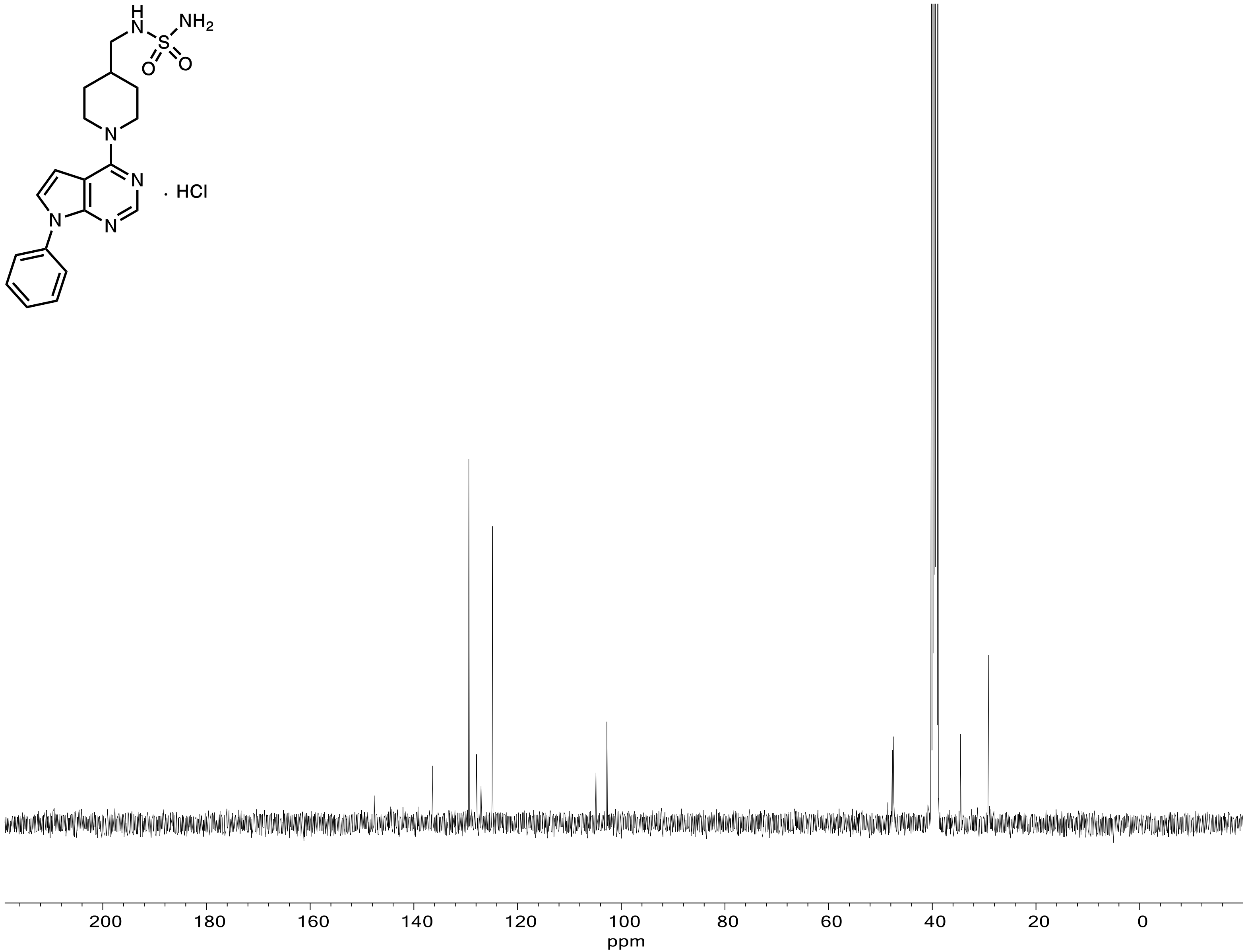

^{13}C NMR (101 MHz, DMSO-d_6) of compound **18g**.

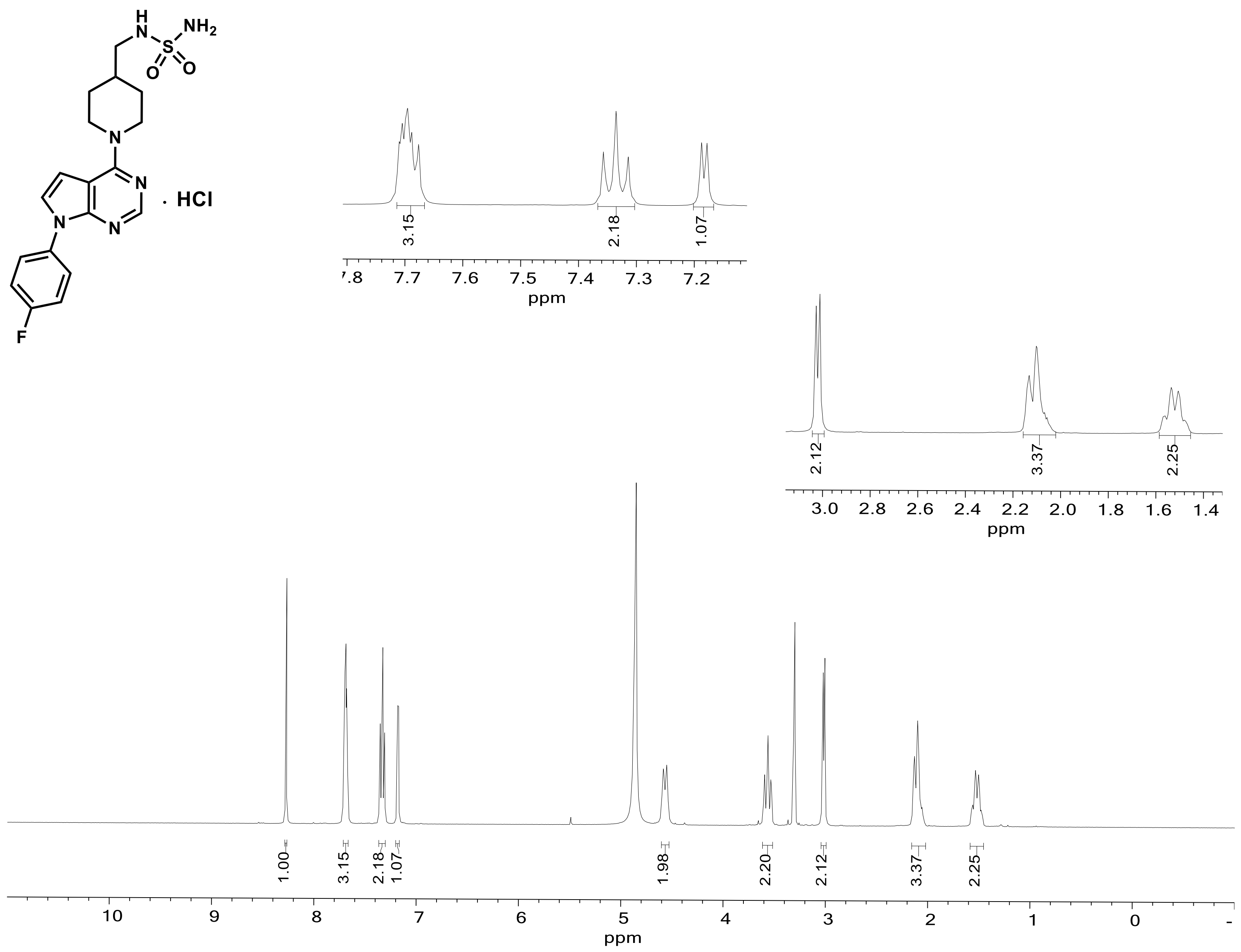

^{1}H NMR (400 MHz, MeOD) of compound **18h**.

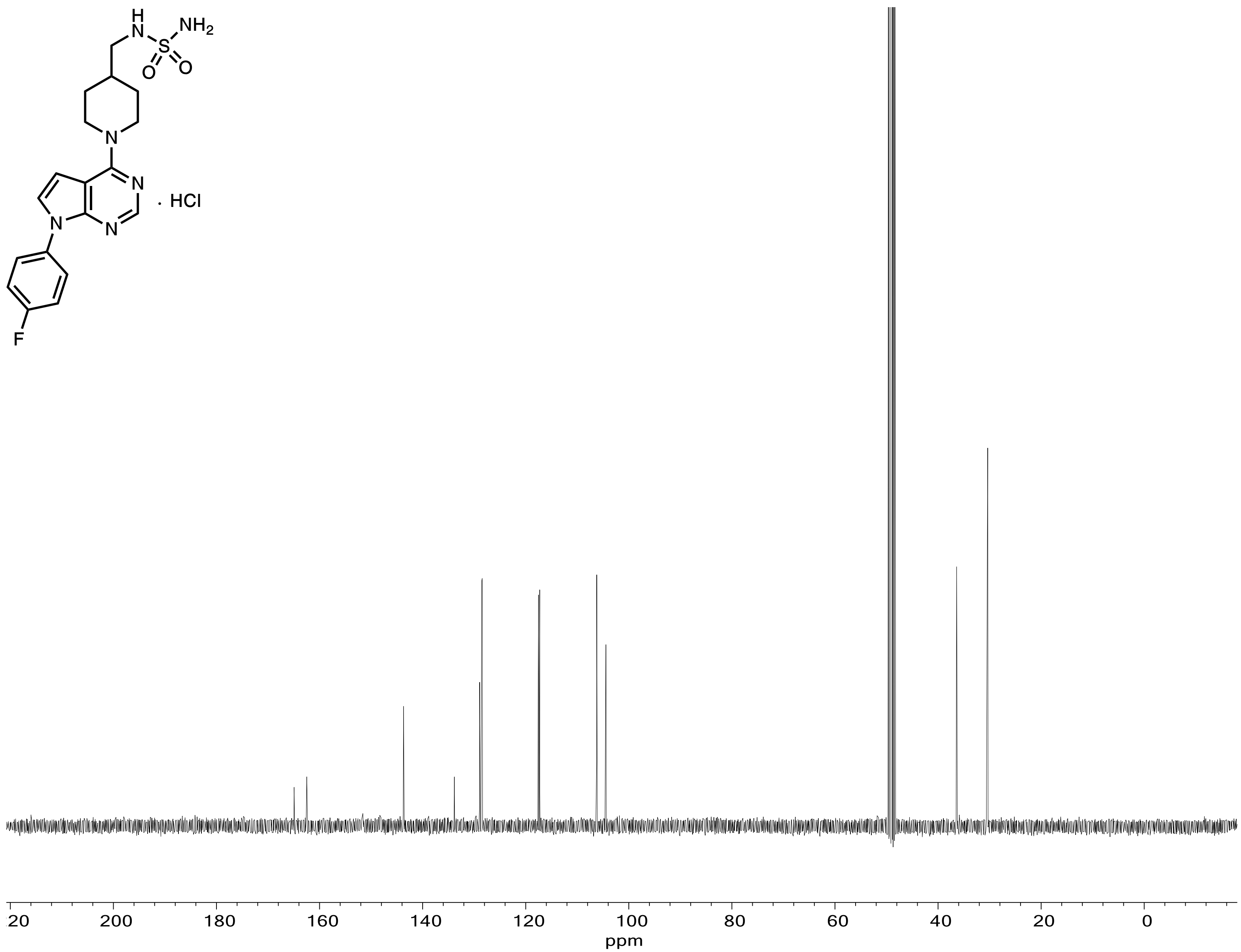

^{13}C NMR (101 MHz, MeOD) of compound **18h**.

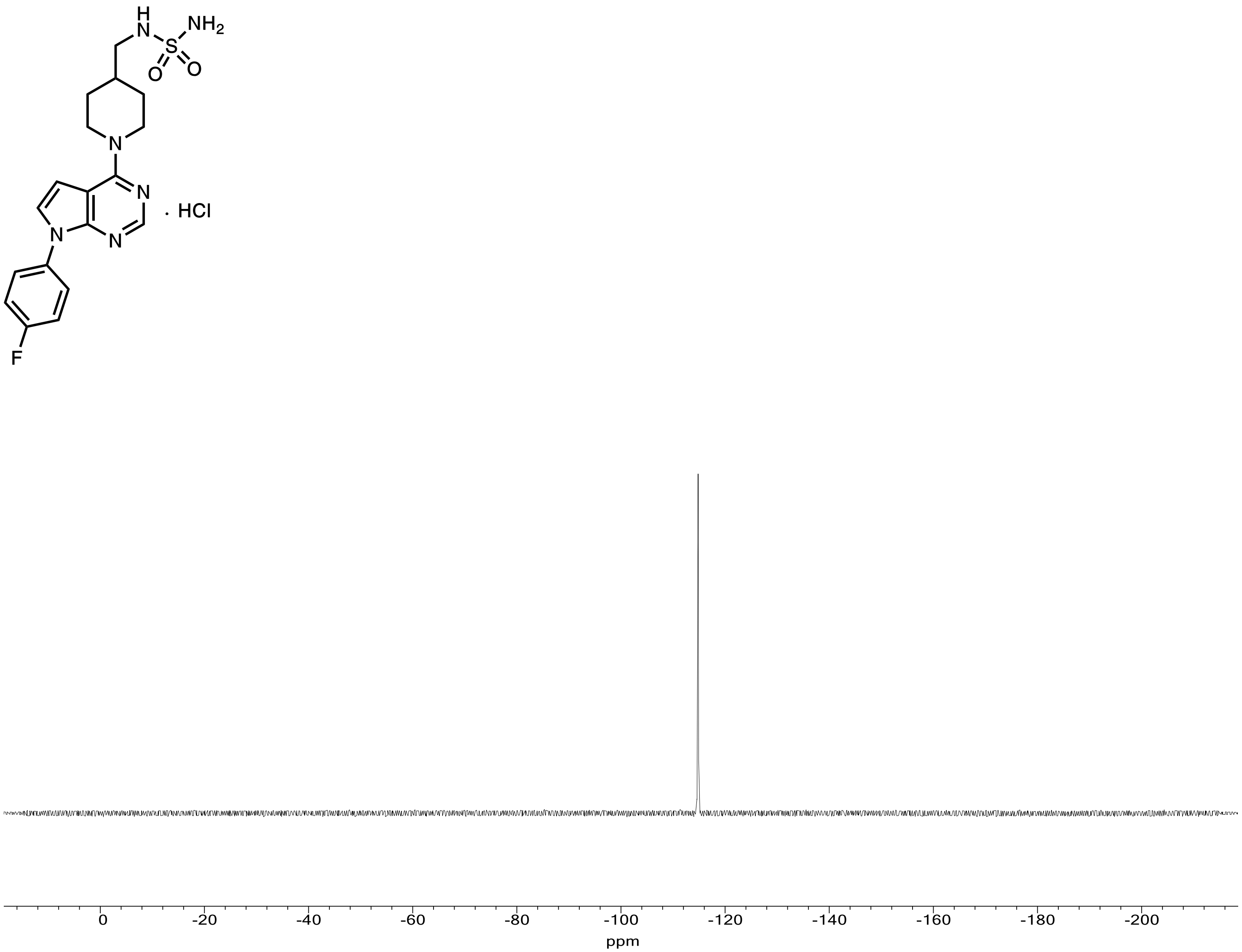

^{19}F NMR (377 MHz, MeOD) of compound **18h**.

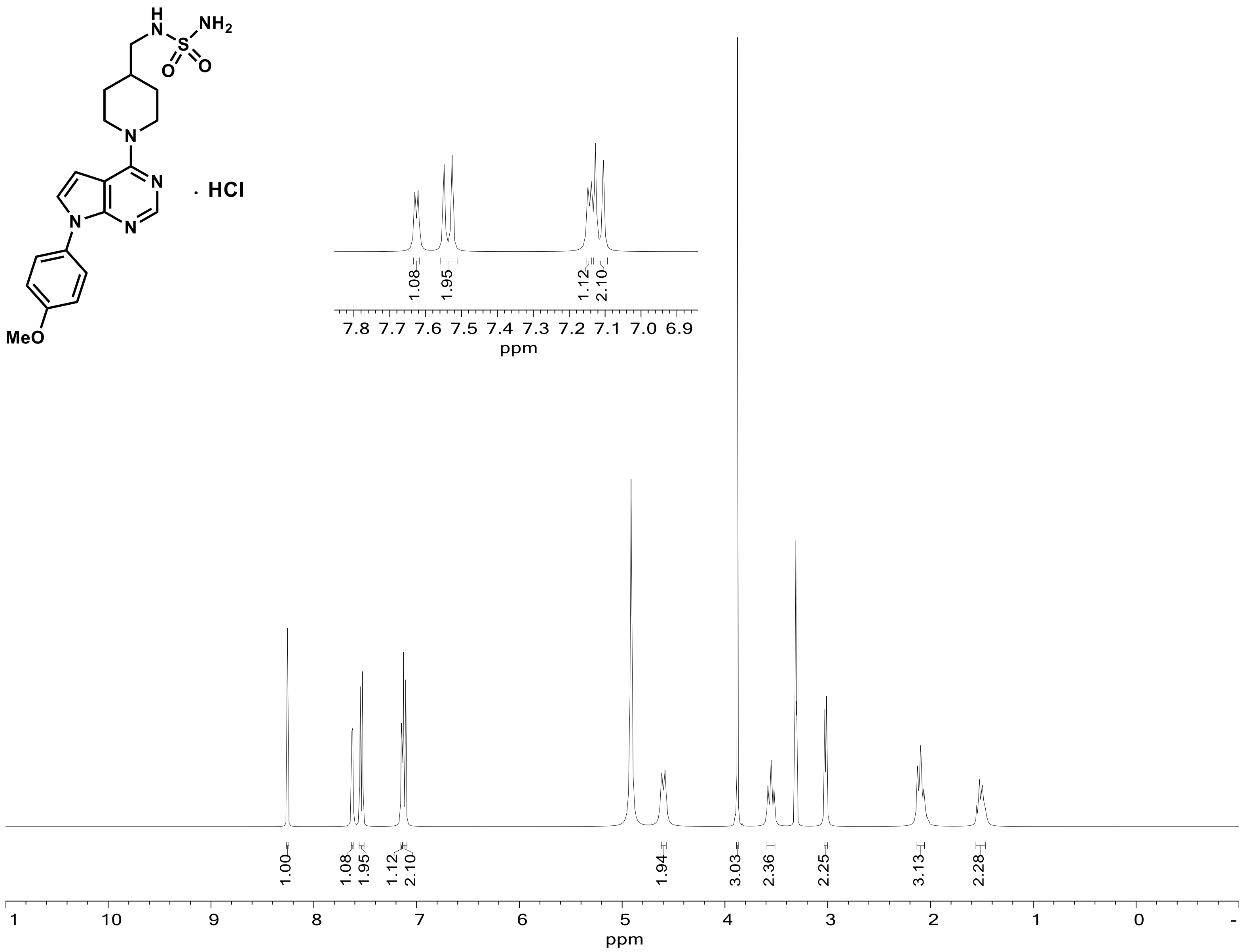

^1H NMR (400 MHz, MeOD) of compound **18i**.

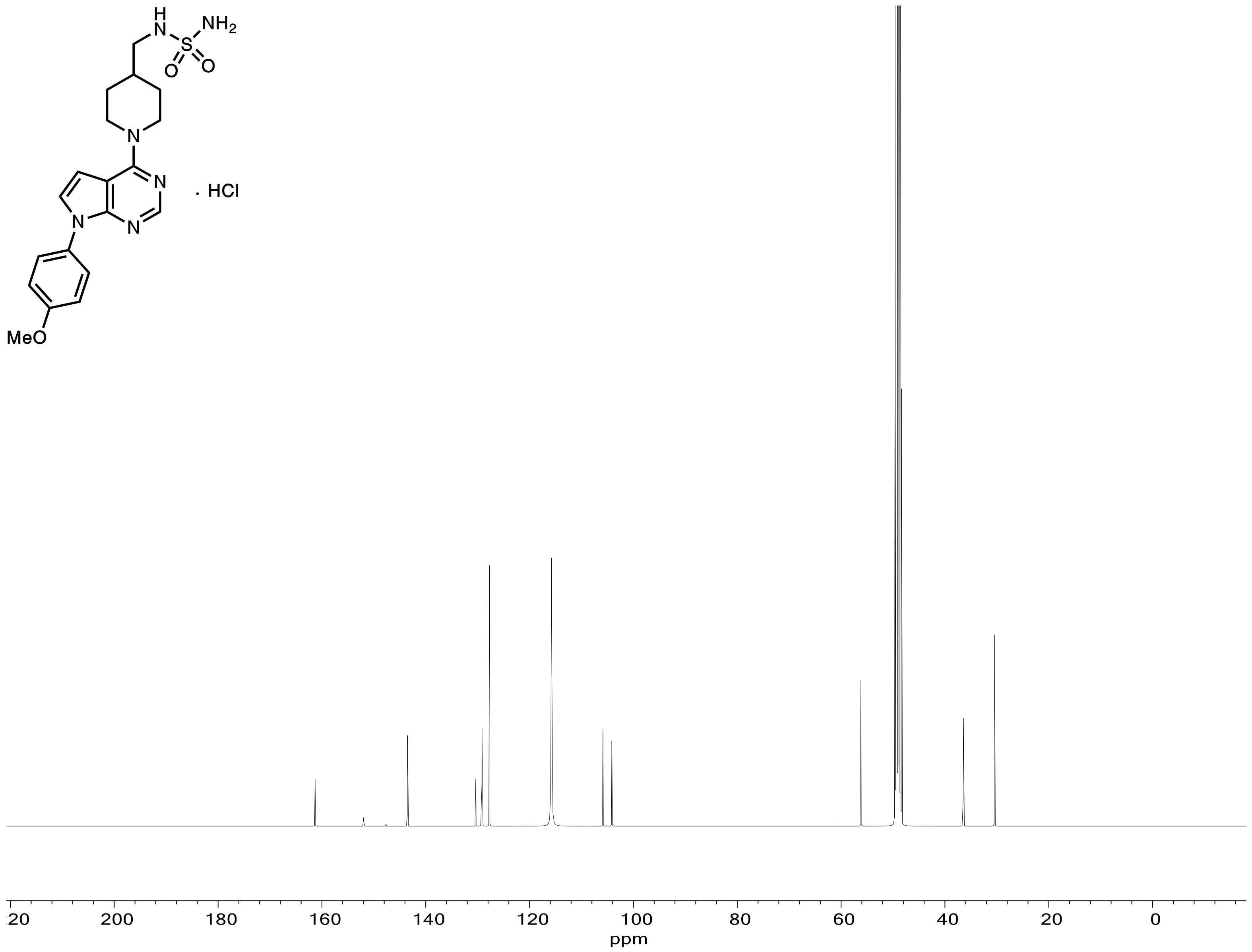

^{13}C NMR (101 MHz, MeOD) of compound **18i**.

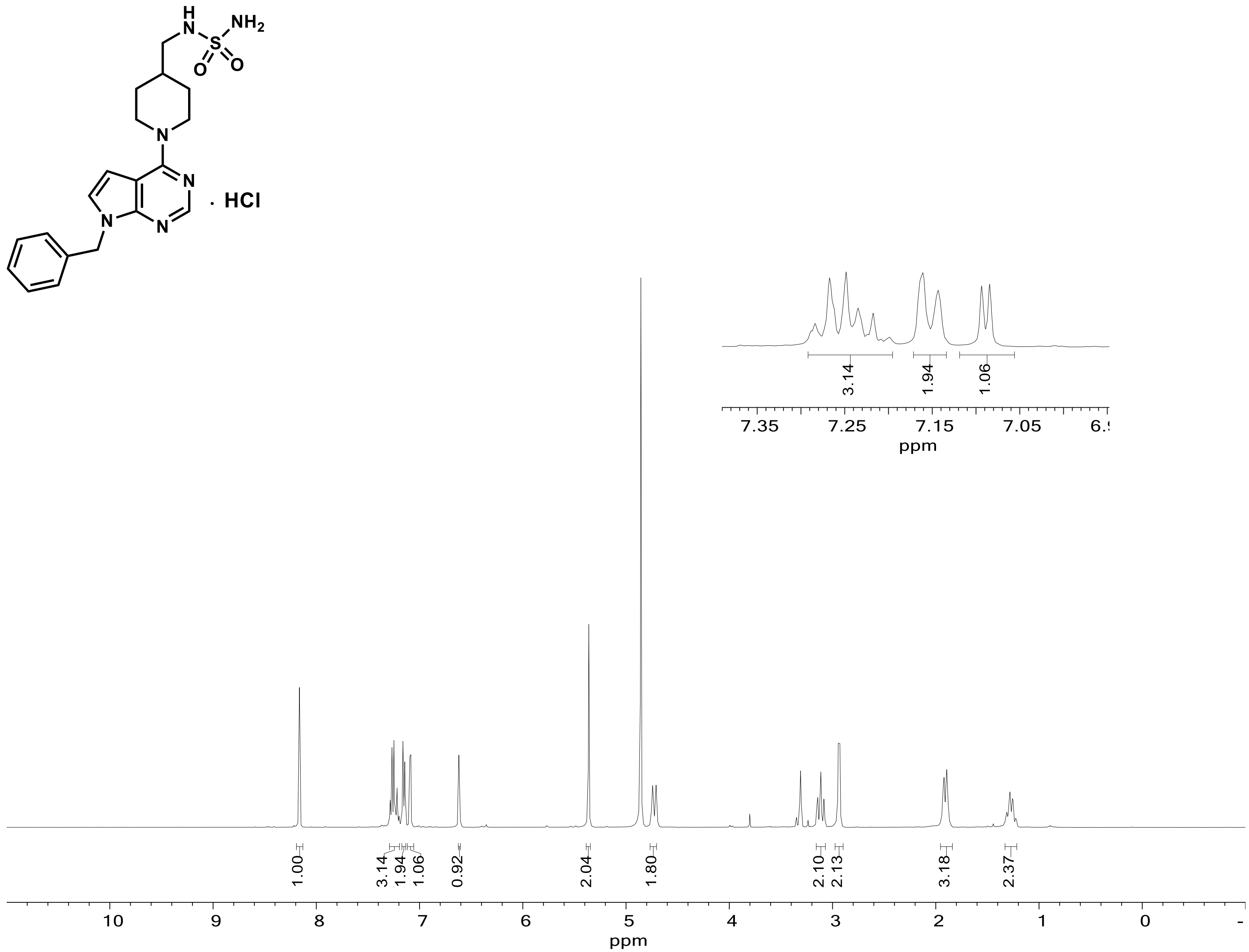

^{1}H NMR (400 MHz, MeOD) of compound **18j**.

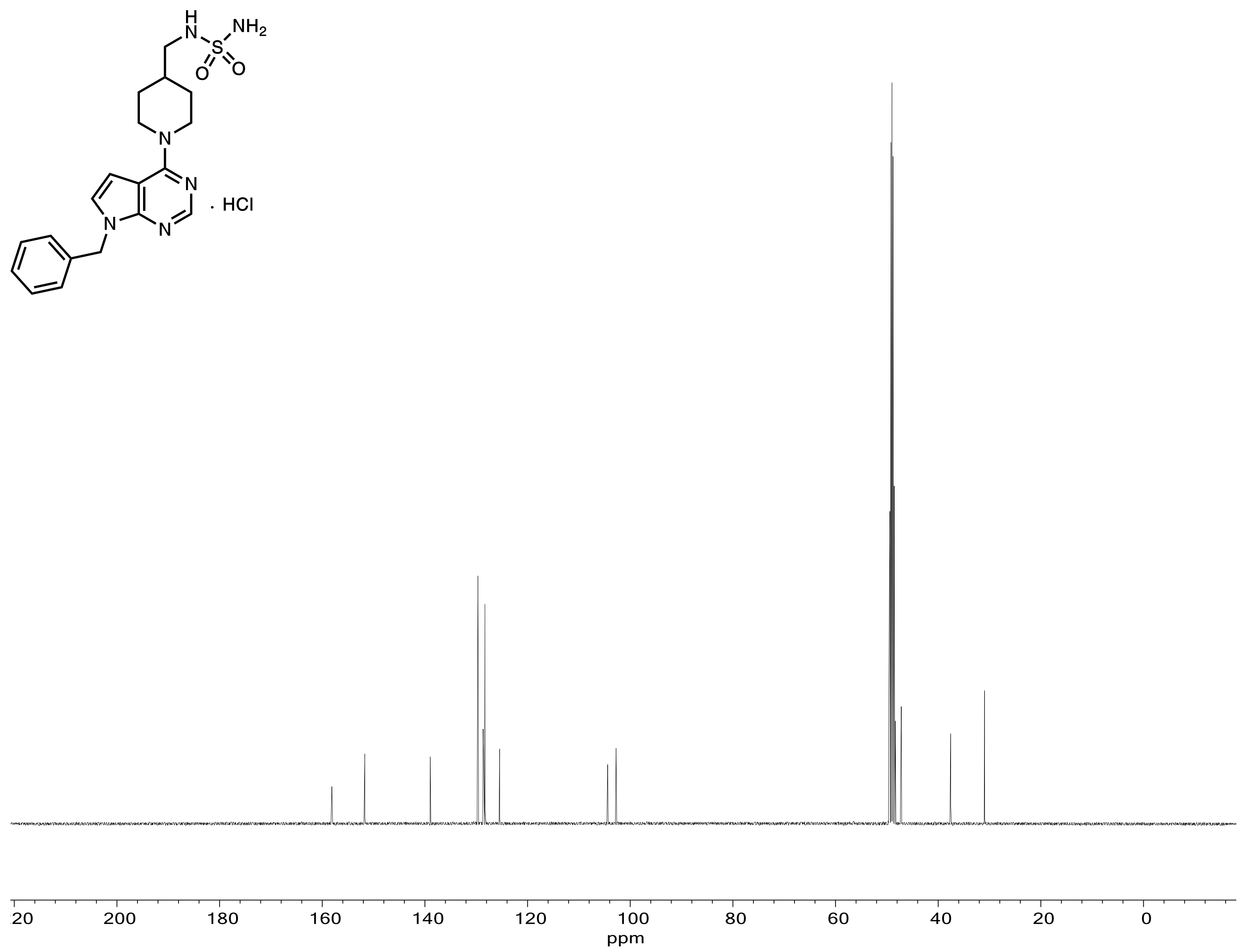

^{13}C NMR (101 MHz, MeOD) of compound **18j**.

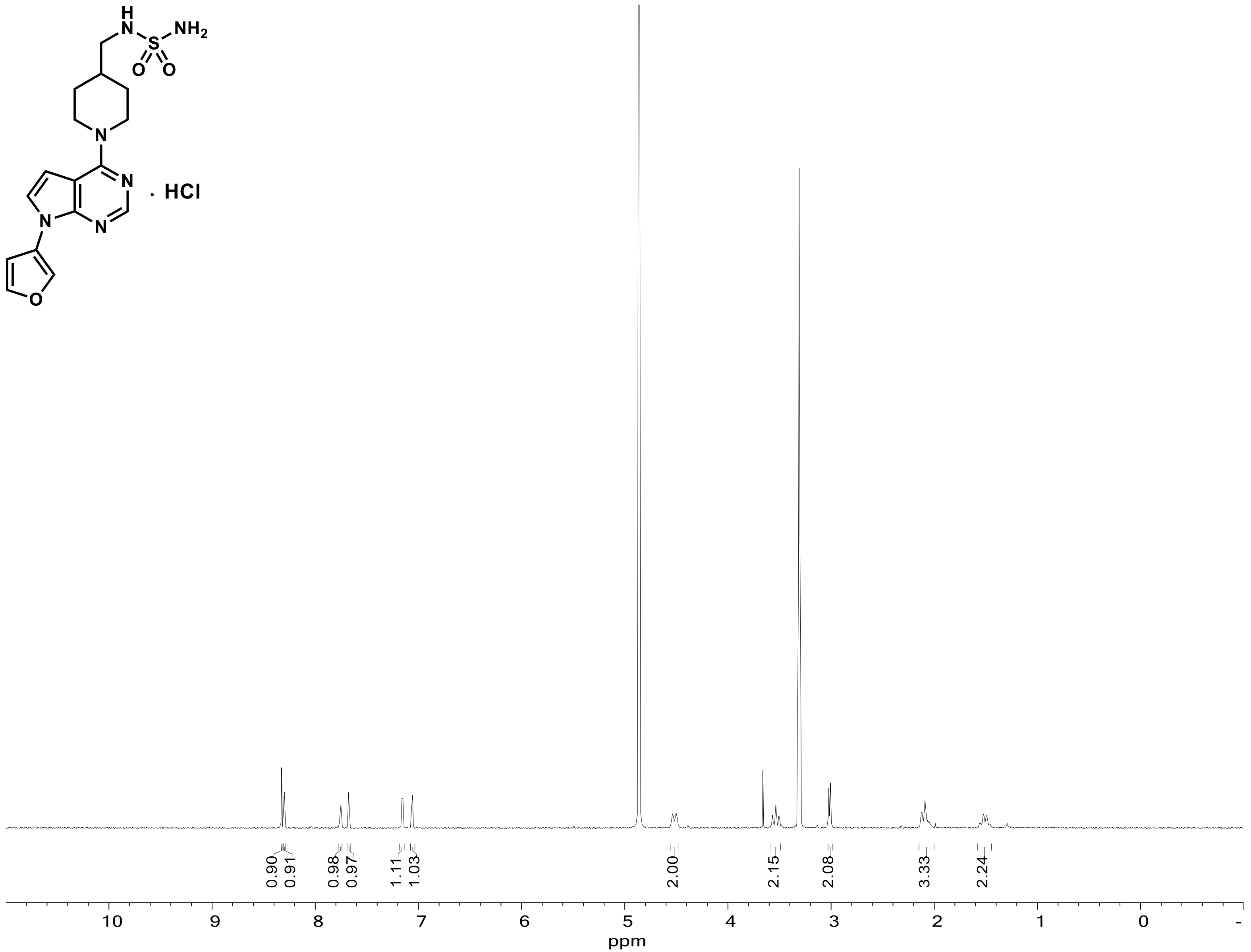

^{1}H NMR (400 MHz, MeOD) of compound **18k**.

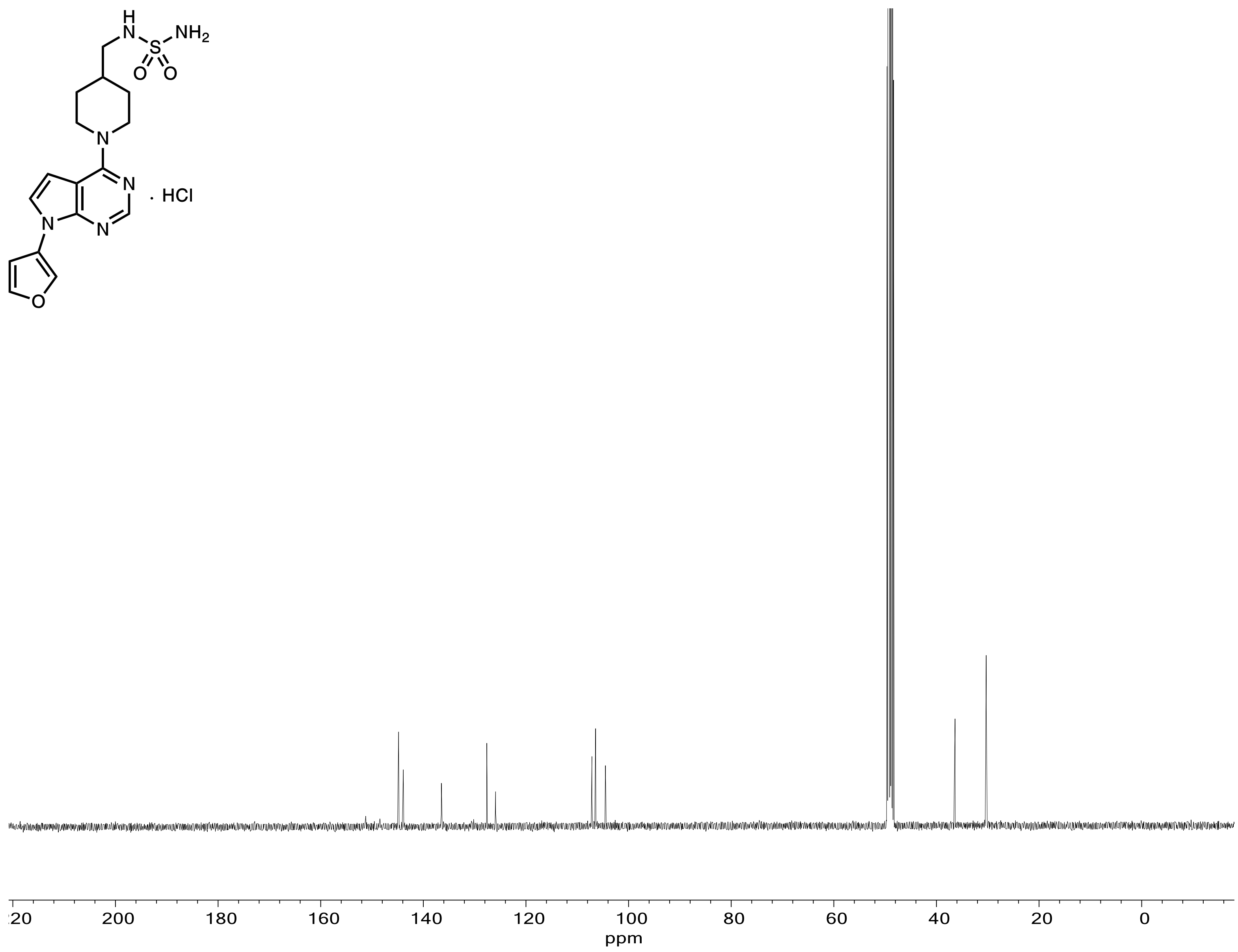

^{13}C NMR (101 MHz, MeOD) of compound **18k**.

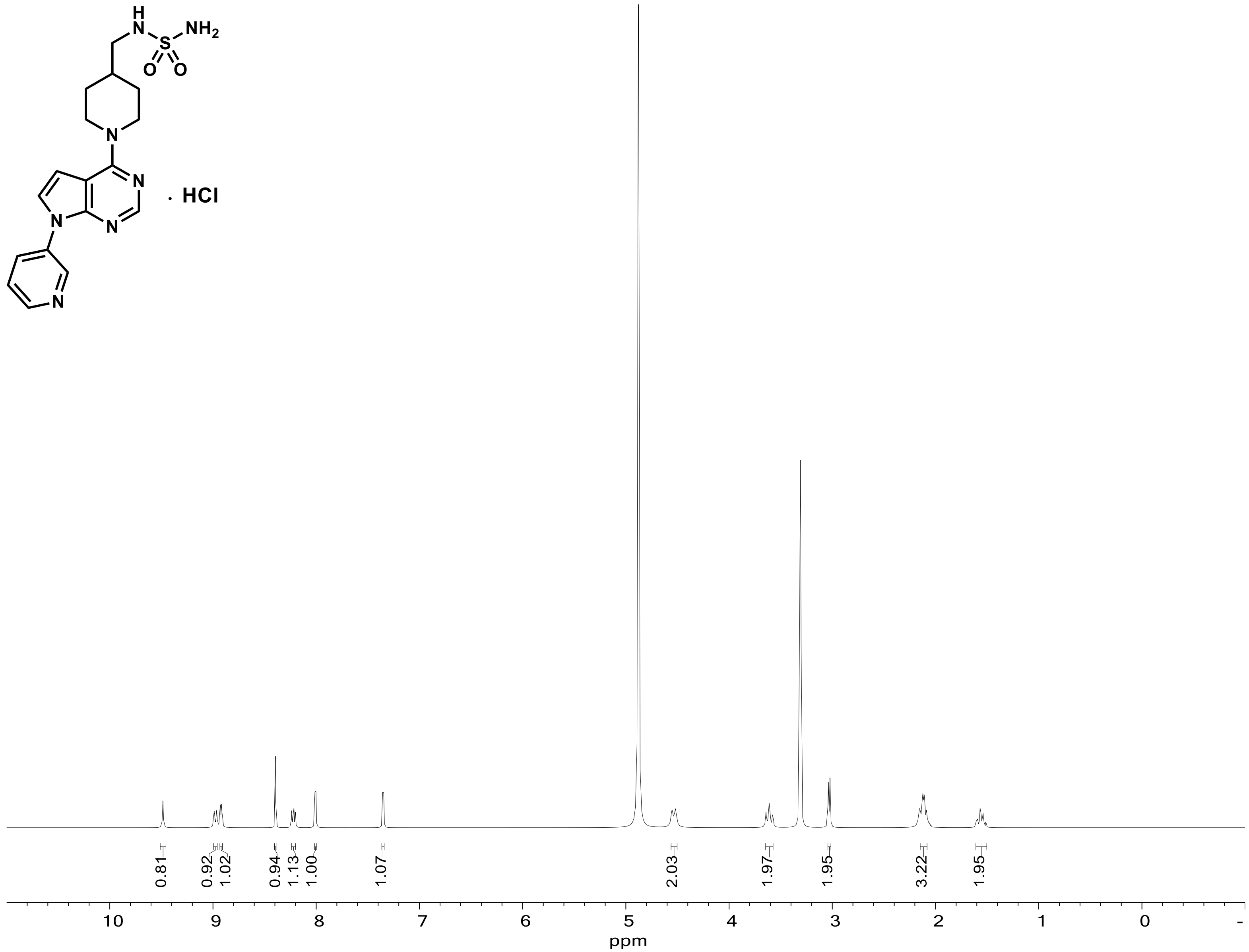

^{1}H NMR (400 MHz, MeOD) of compound **18l**.

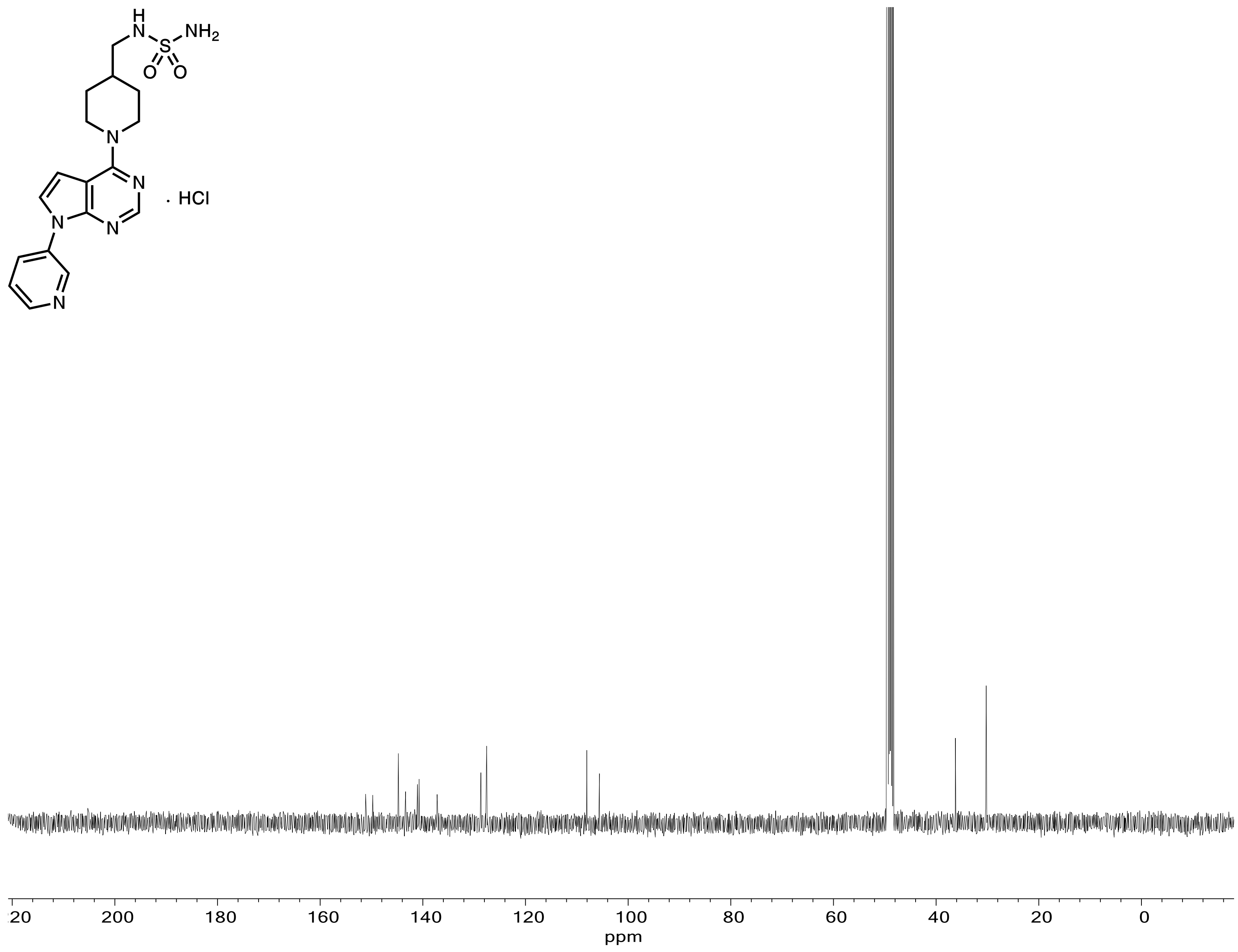

^{13}C NMR (101 MHz, MeOD) of compound **18l**.

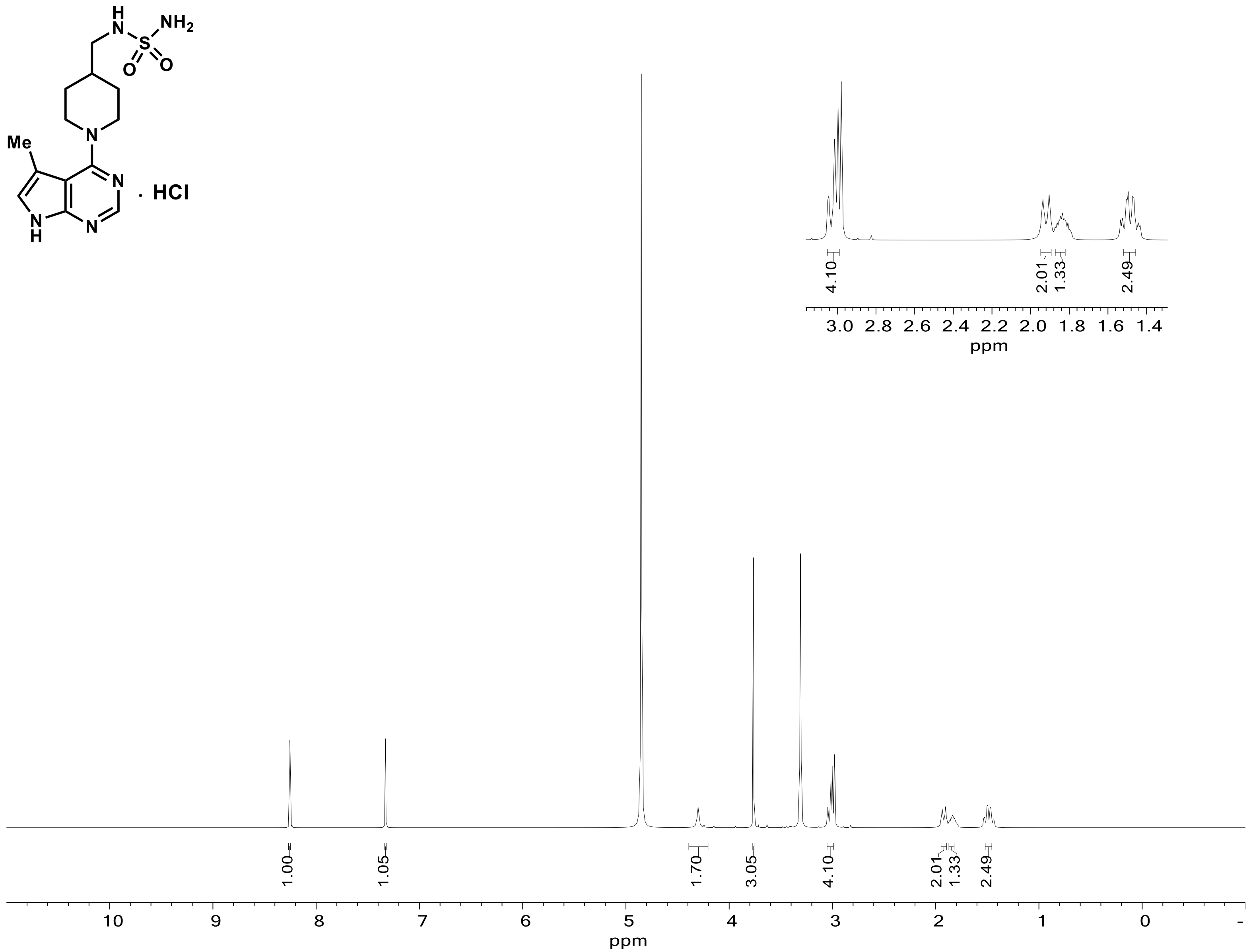

^{1}H NMR (400 MHz, MeOD) of compound **18m**.

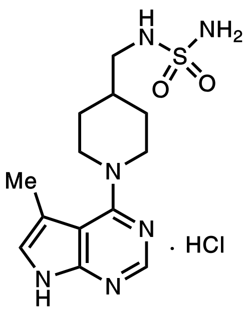

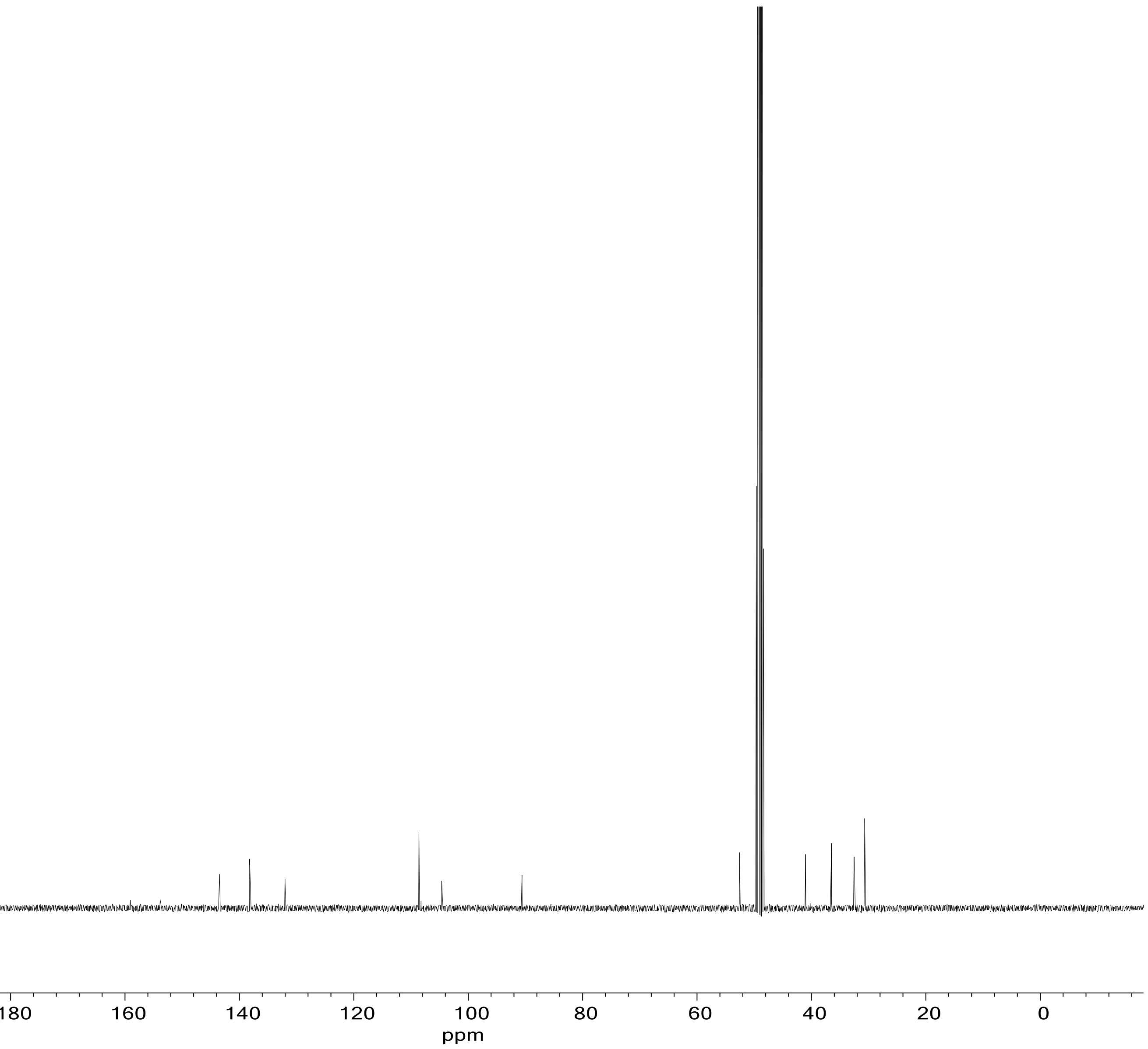

^{13}C NMR (101 MHz, MeOD) of compound **18m**.

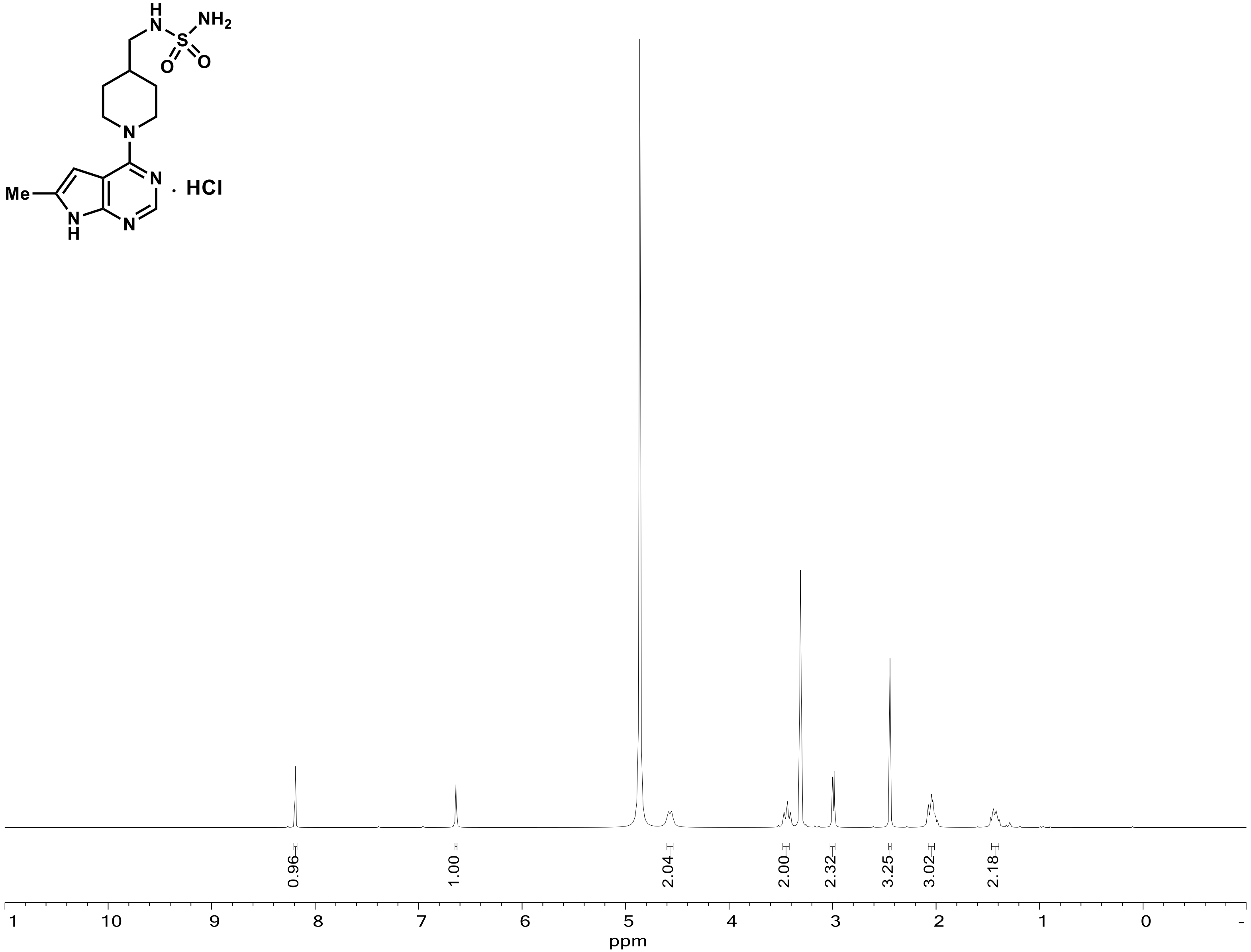

^{1}H NMR (400 MHz, MeOD) of compound **18n**.

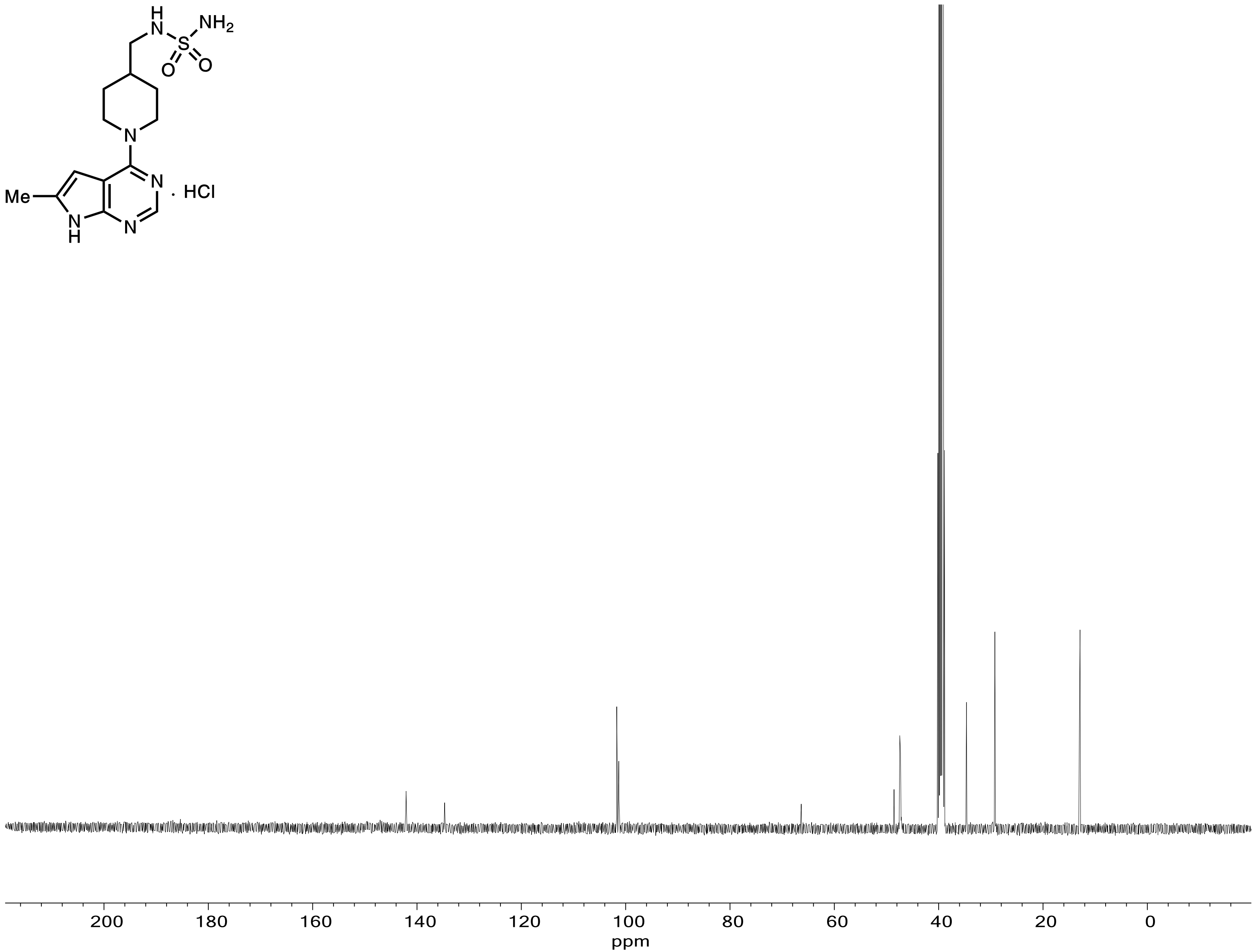

^{13}C NMR (101 MHz, DMSO-d_6) of compound **18n**.

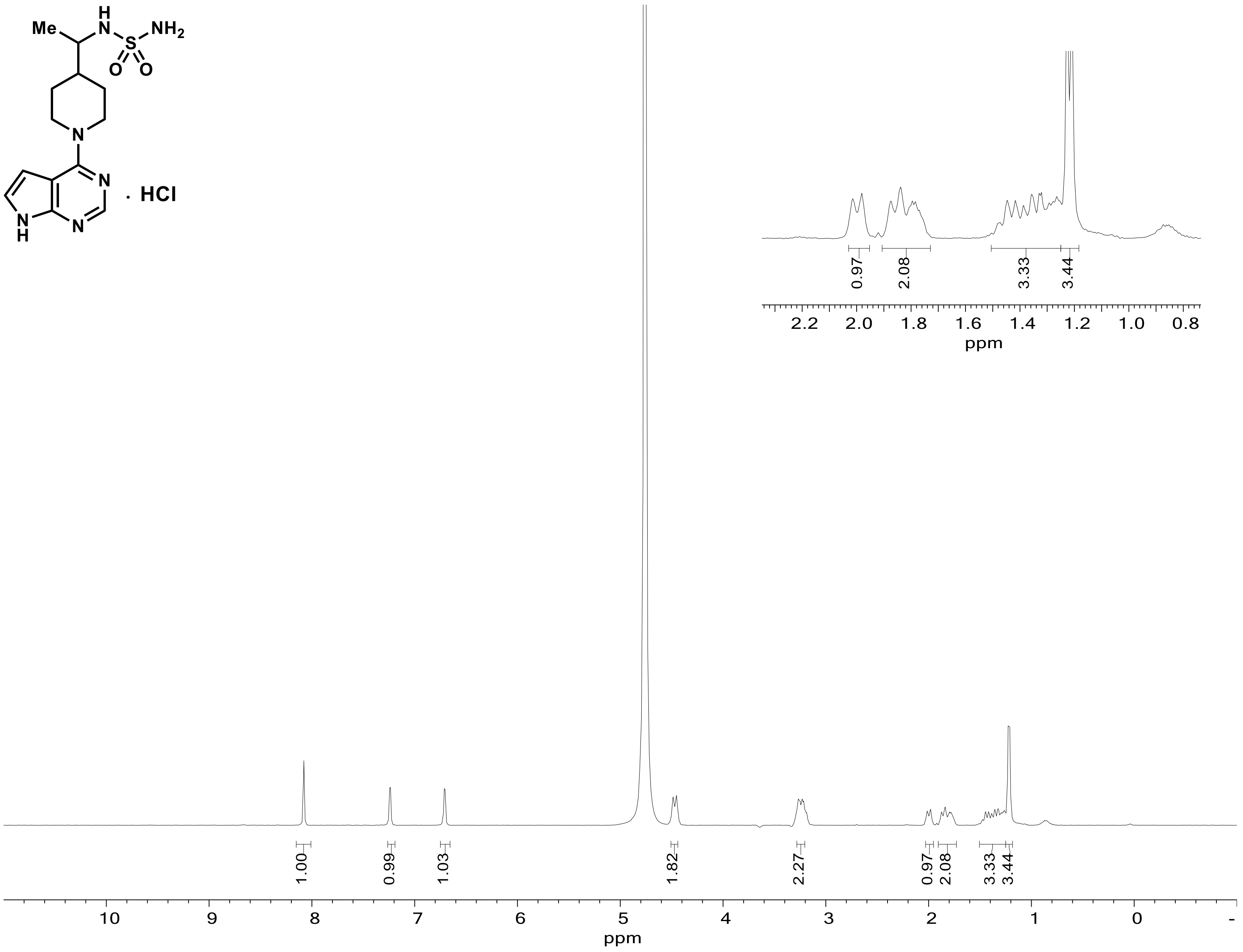

^{1}H NMR (400 MHz, MeOD) of compound **18o**.

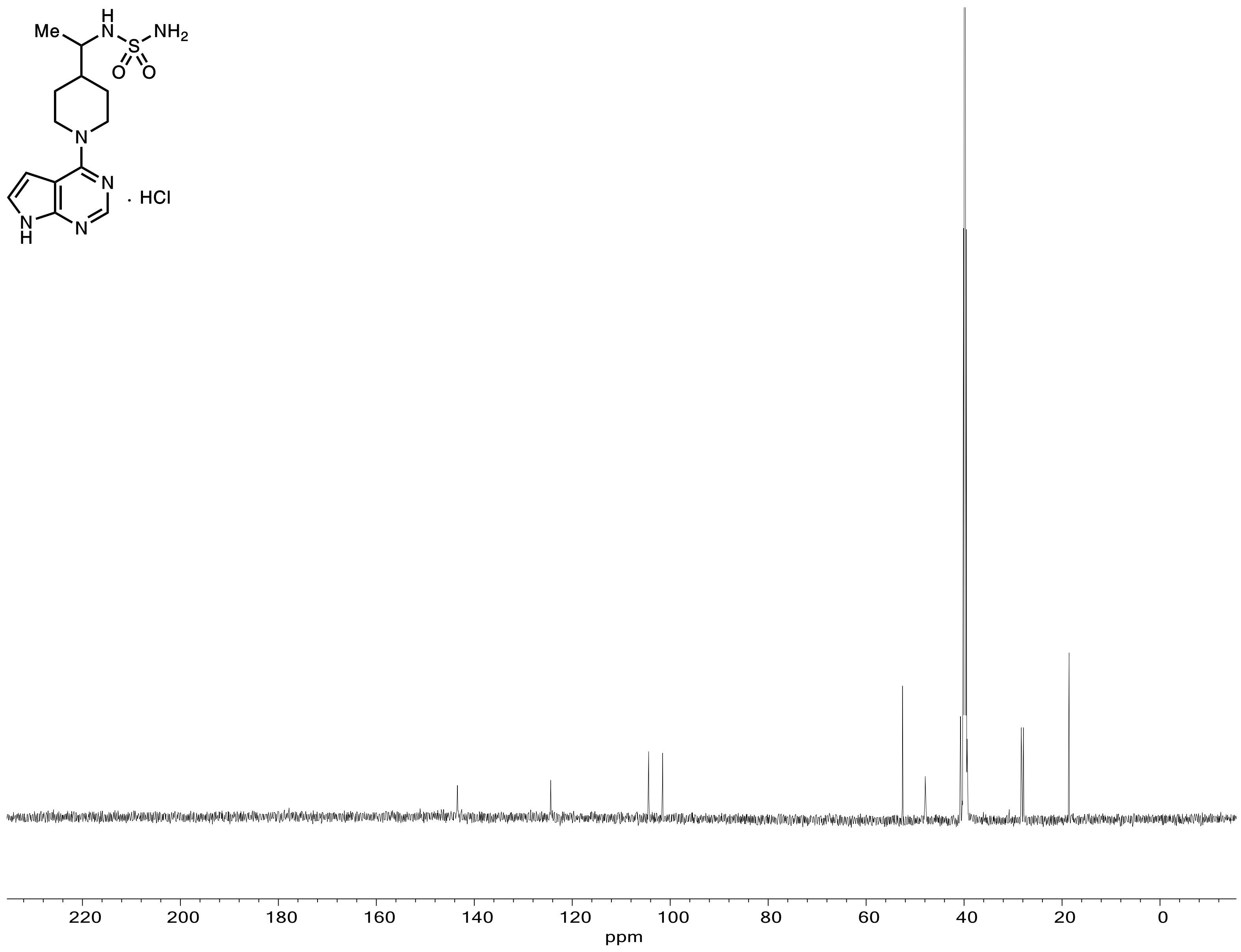

^{13}C NMR (101 MHz, DMSO-d_6) of compound **18o**.

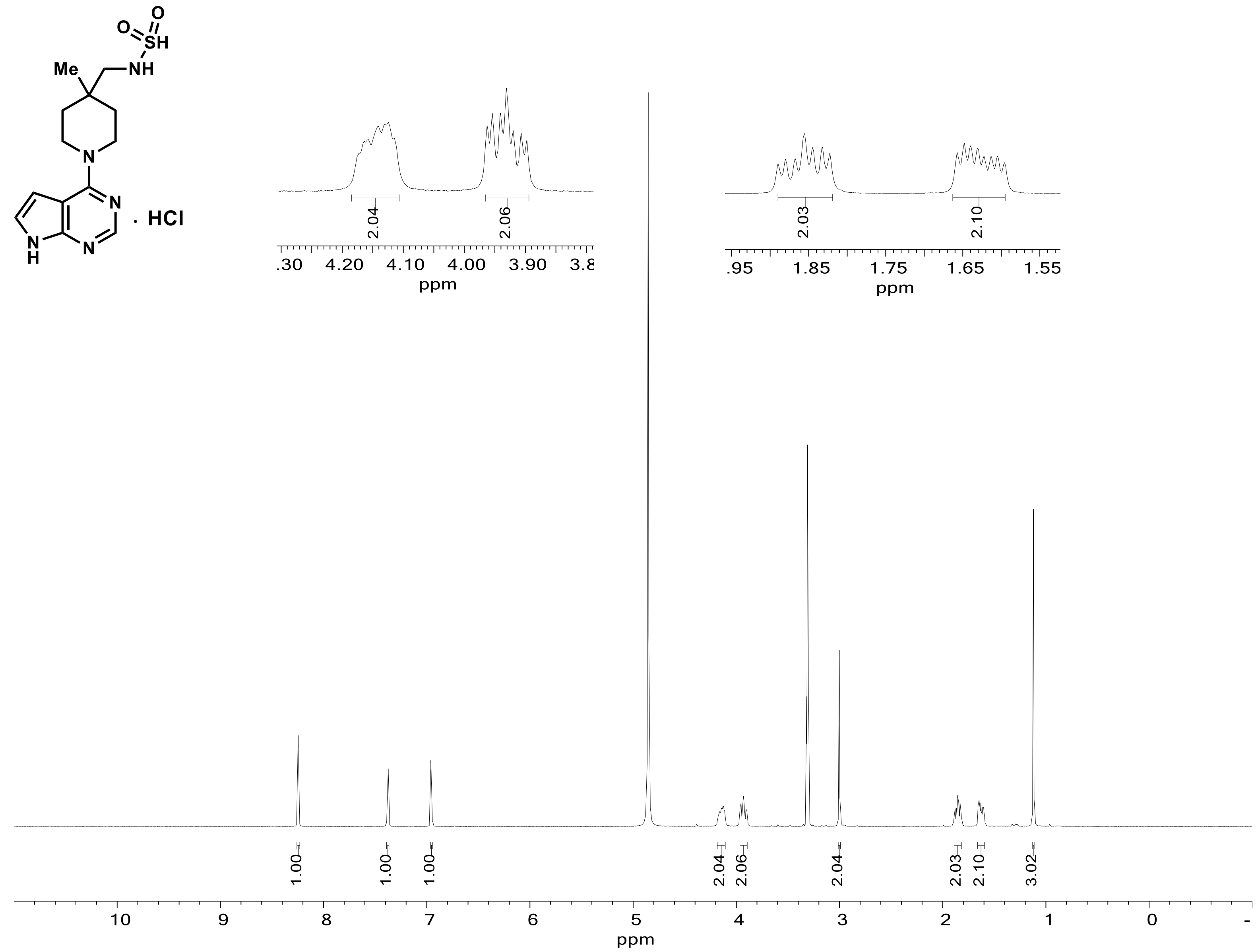

^{1}H NMR (400 MHz, MeOD) of compound **18p**.

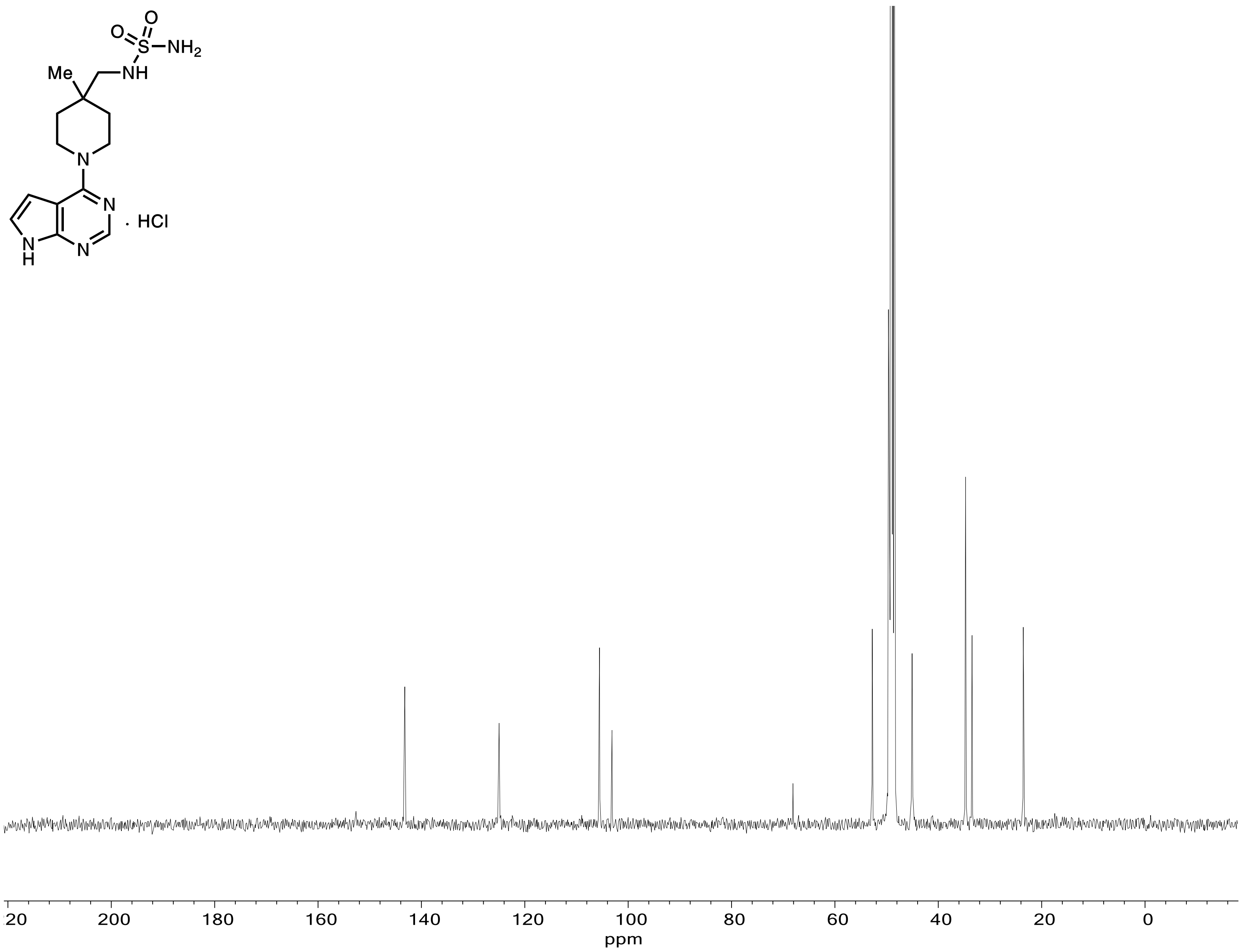

^{13}C NMR (101 MHz, MeOD) of compound **18p**.

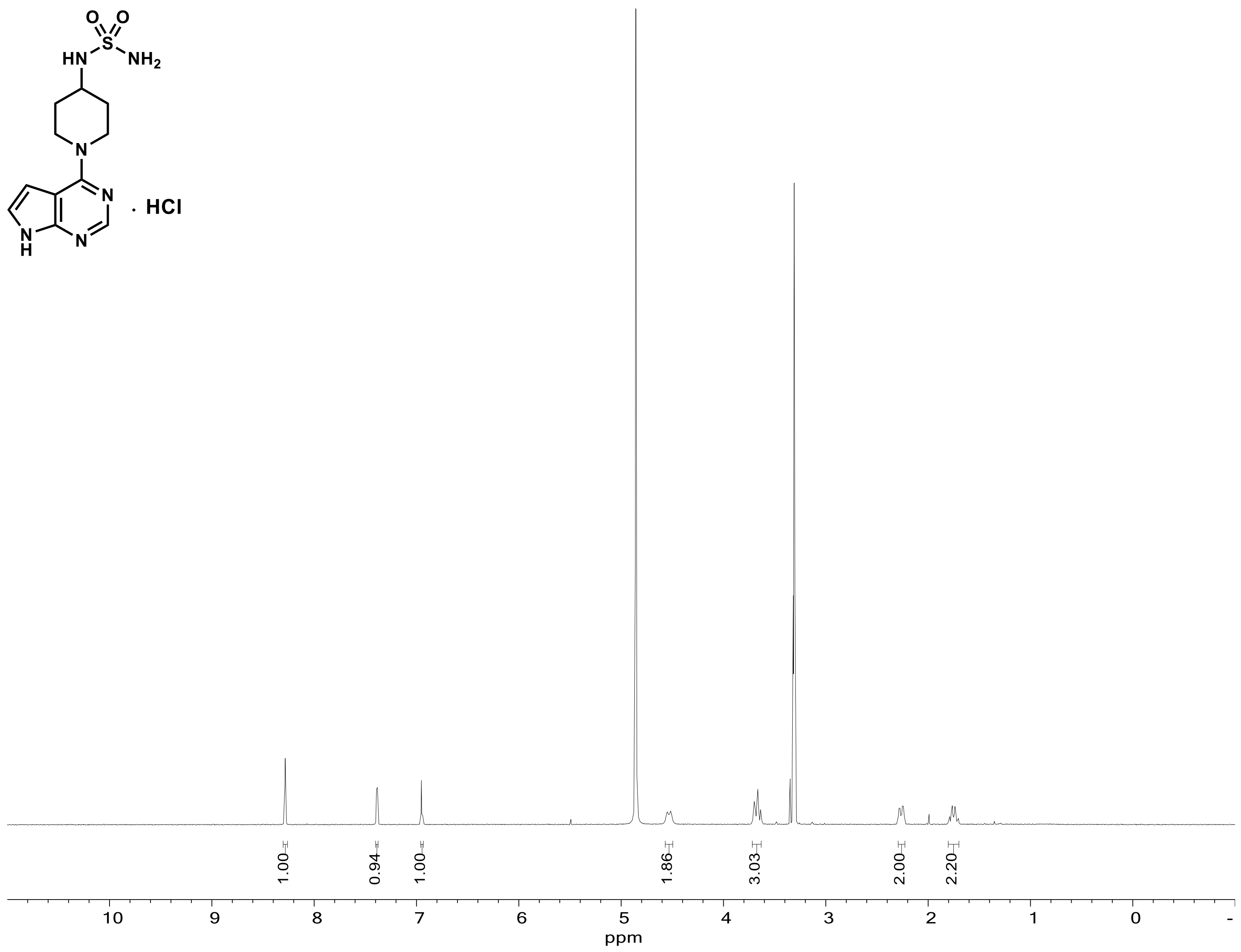

^{1}H NMR (400 MHz, MeOD) of compound **18q**.

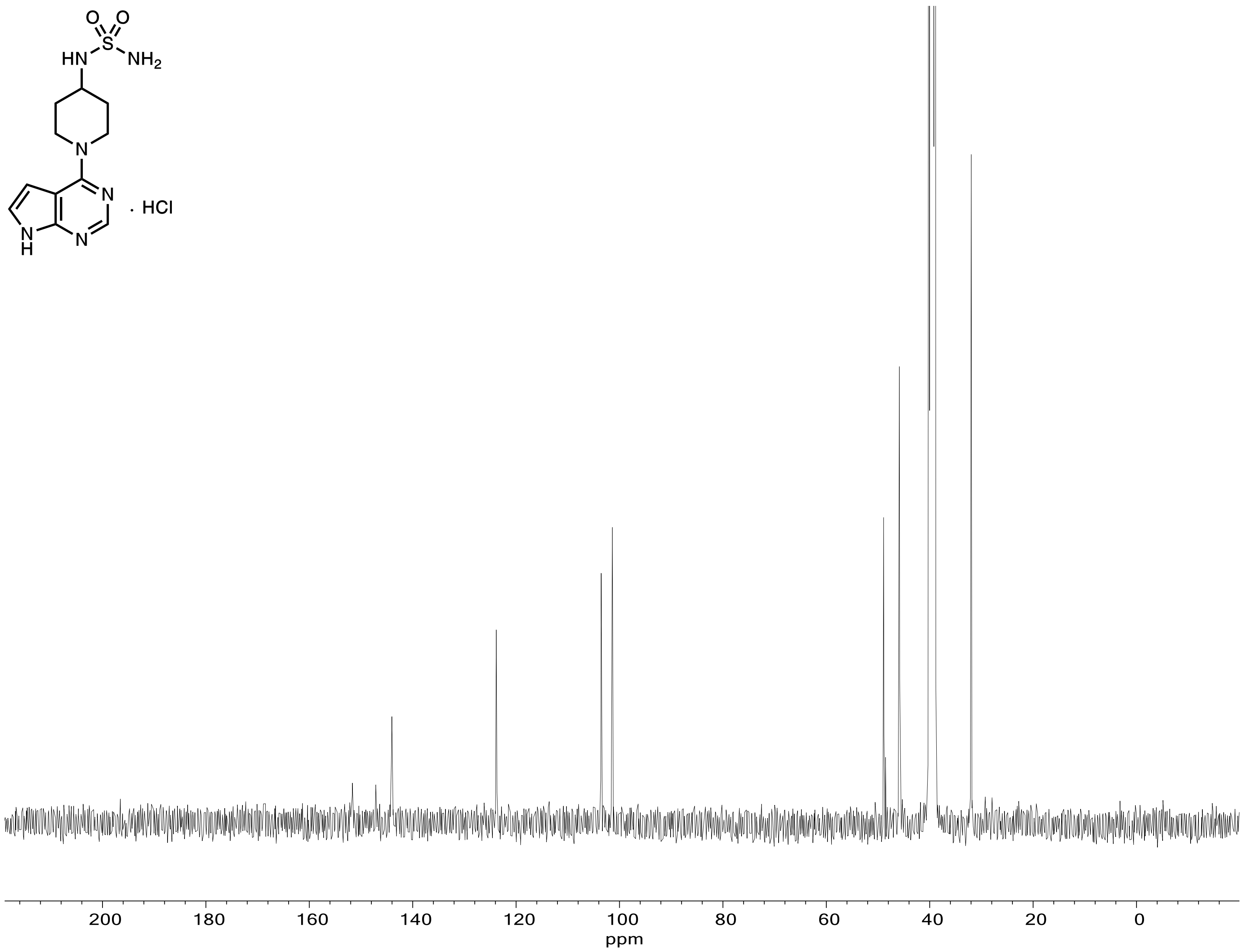

^{13}C NMR (101 MHz, DMSO-d_6) of compound **18q**.

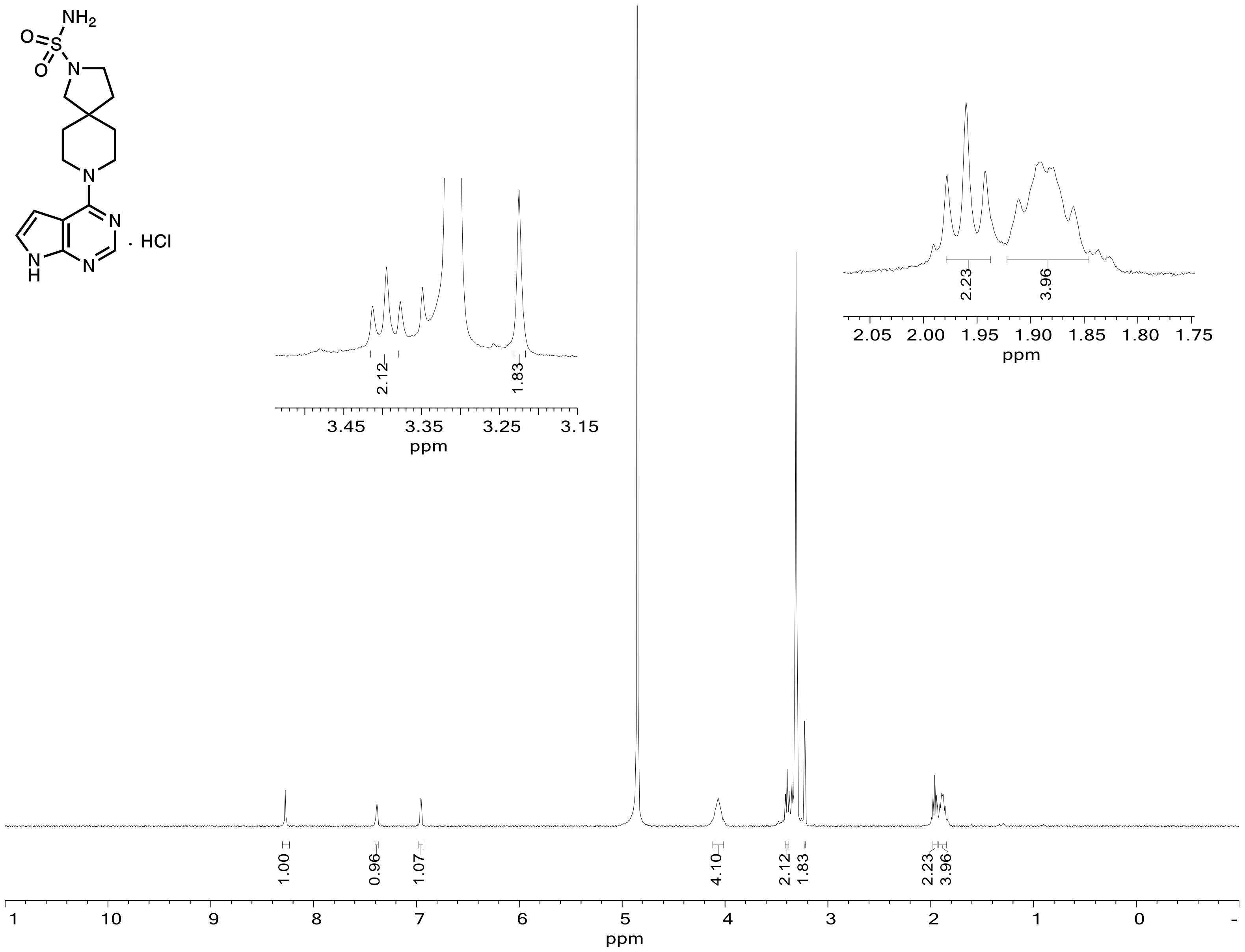

^{1}H NMR (400 MHz, MeOD) of compound **18r**.

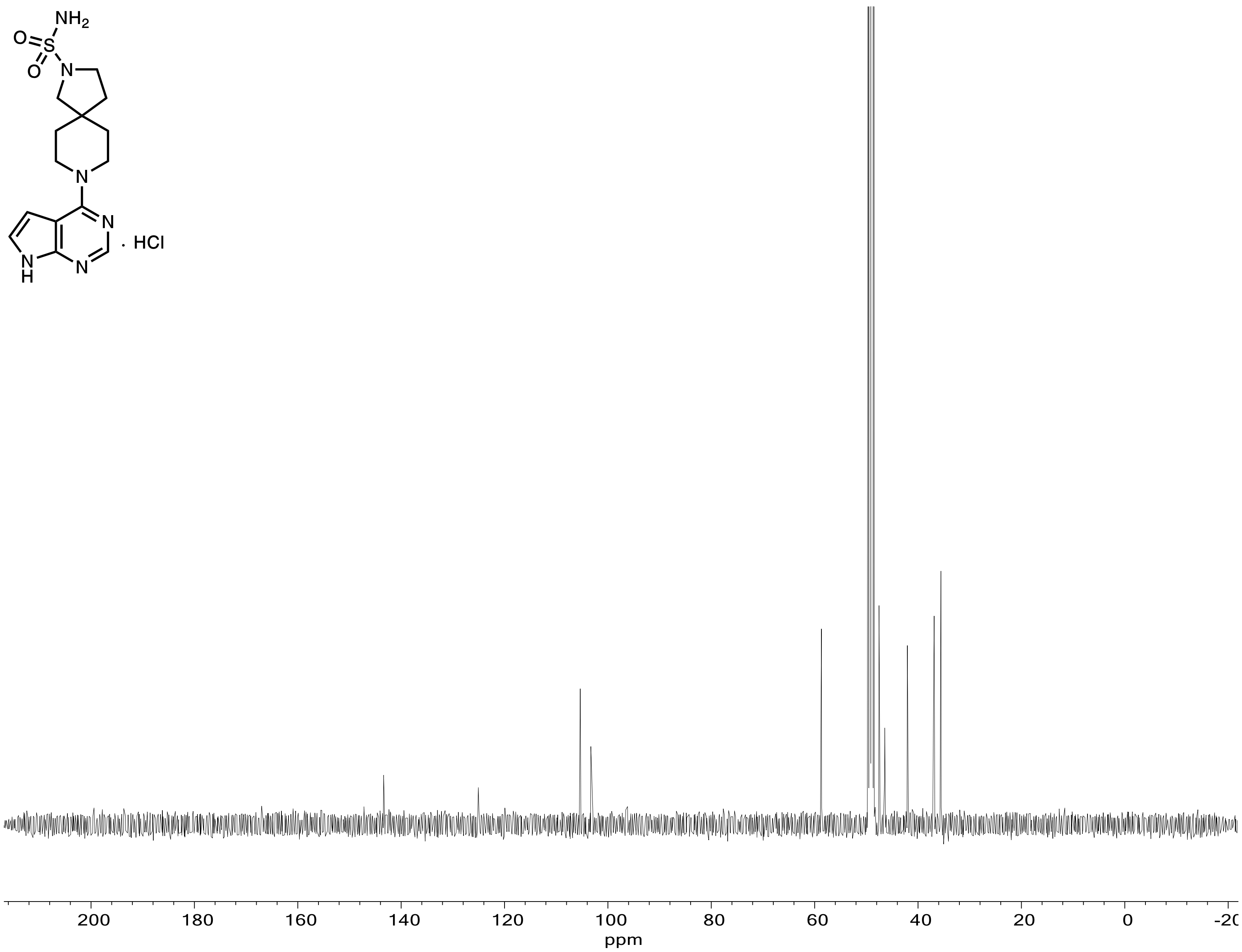

^{13}C NMR (101 MHz, DMSO-d_6) of compound **18r**.

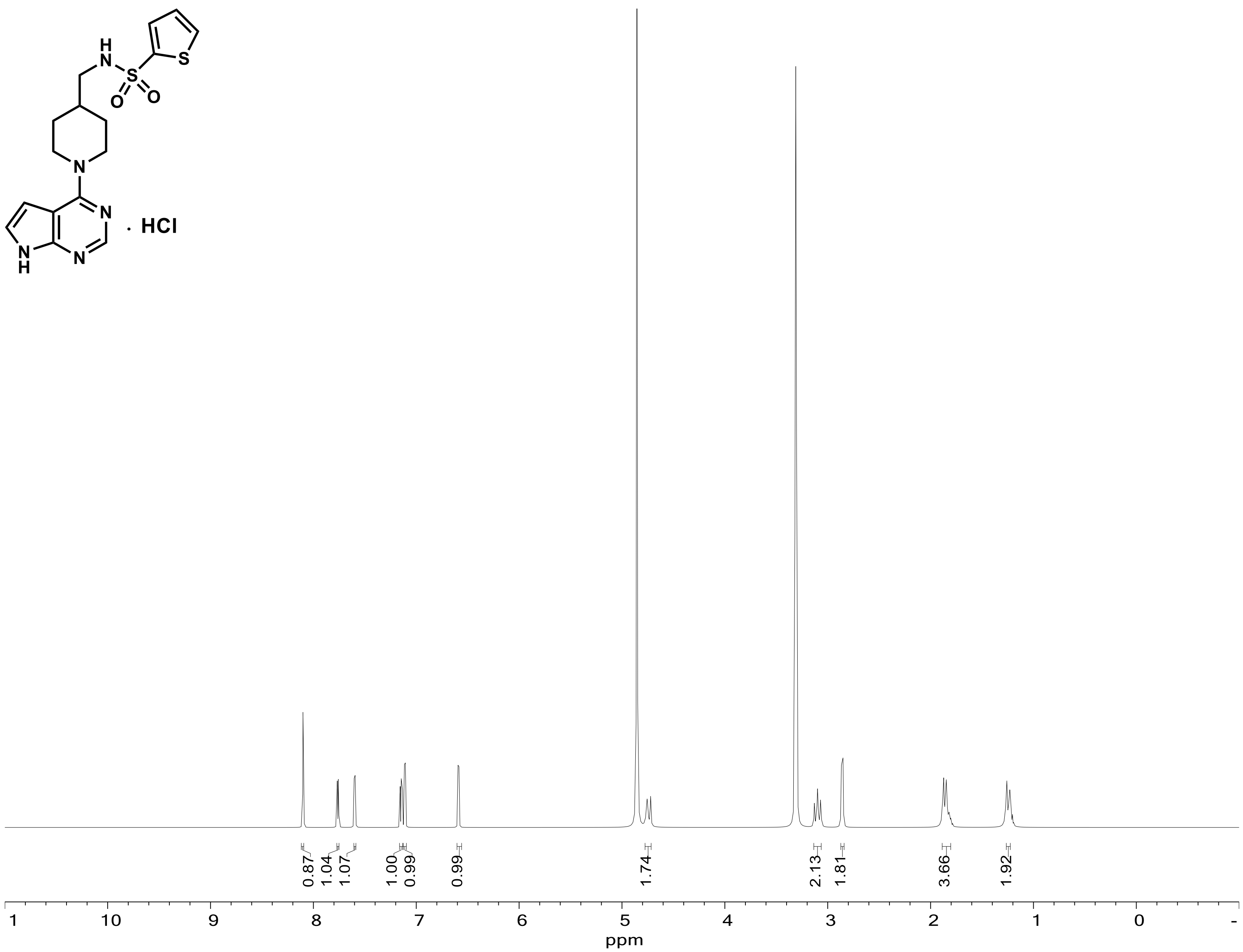

^{1}H NMR (400 MHz, MeOD) of compound **20a**.

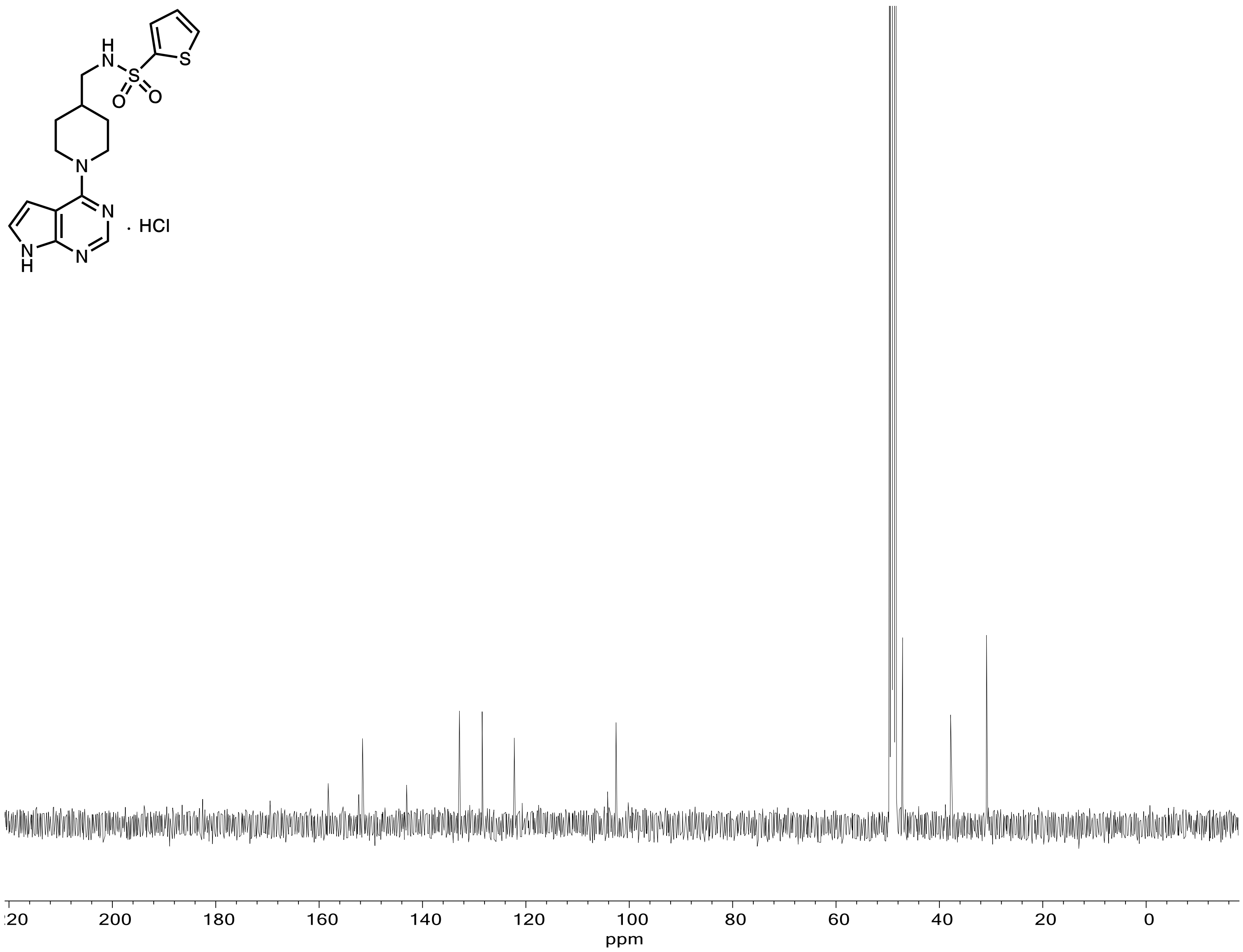

^{13}C NMR (101 MHz, MeOD) of compound **20a**.

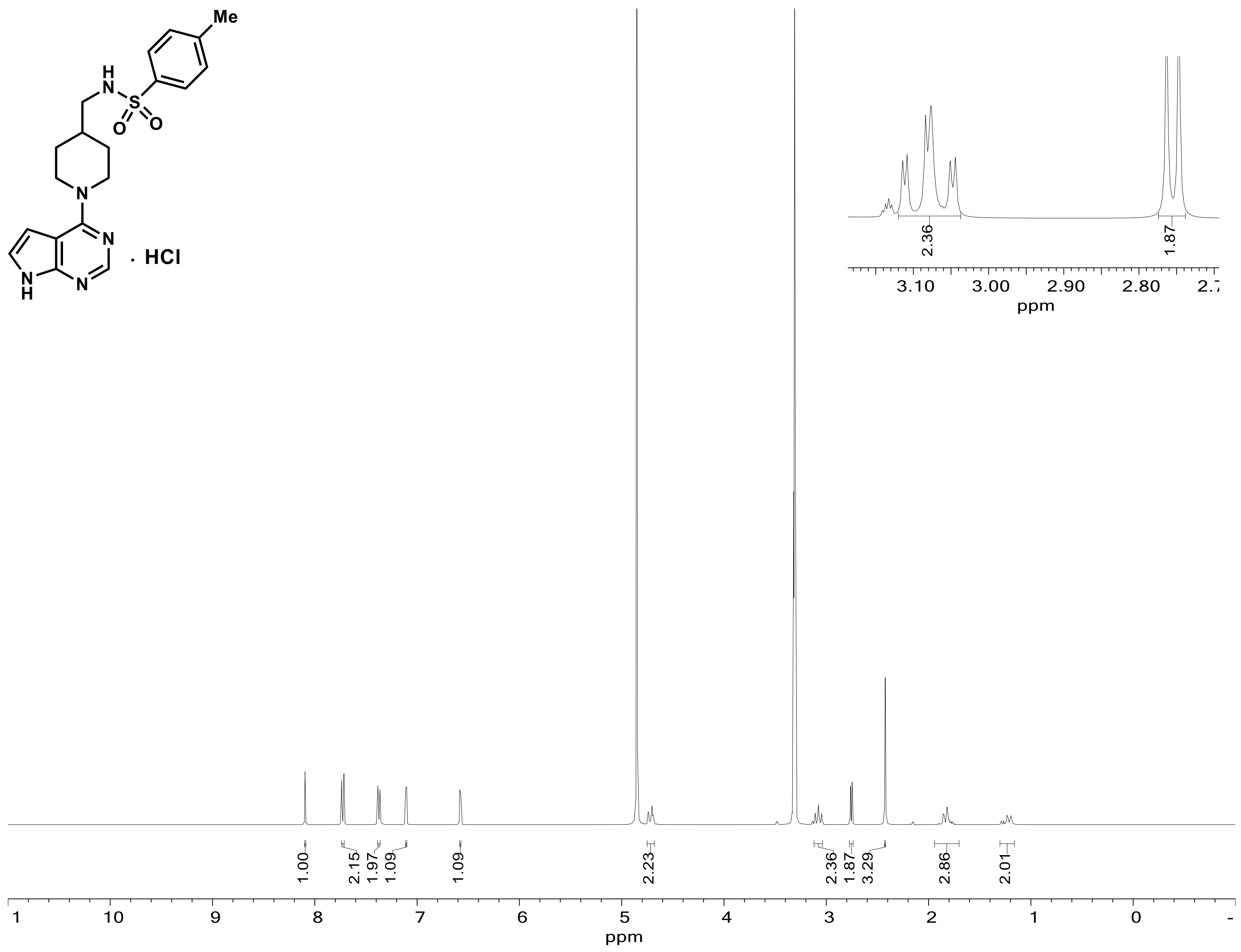

^{1}H NMR (400 MHz, MeOD) of compound **20b**.

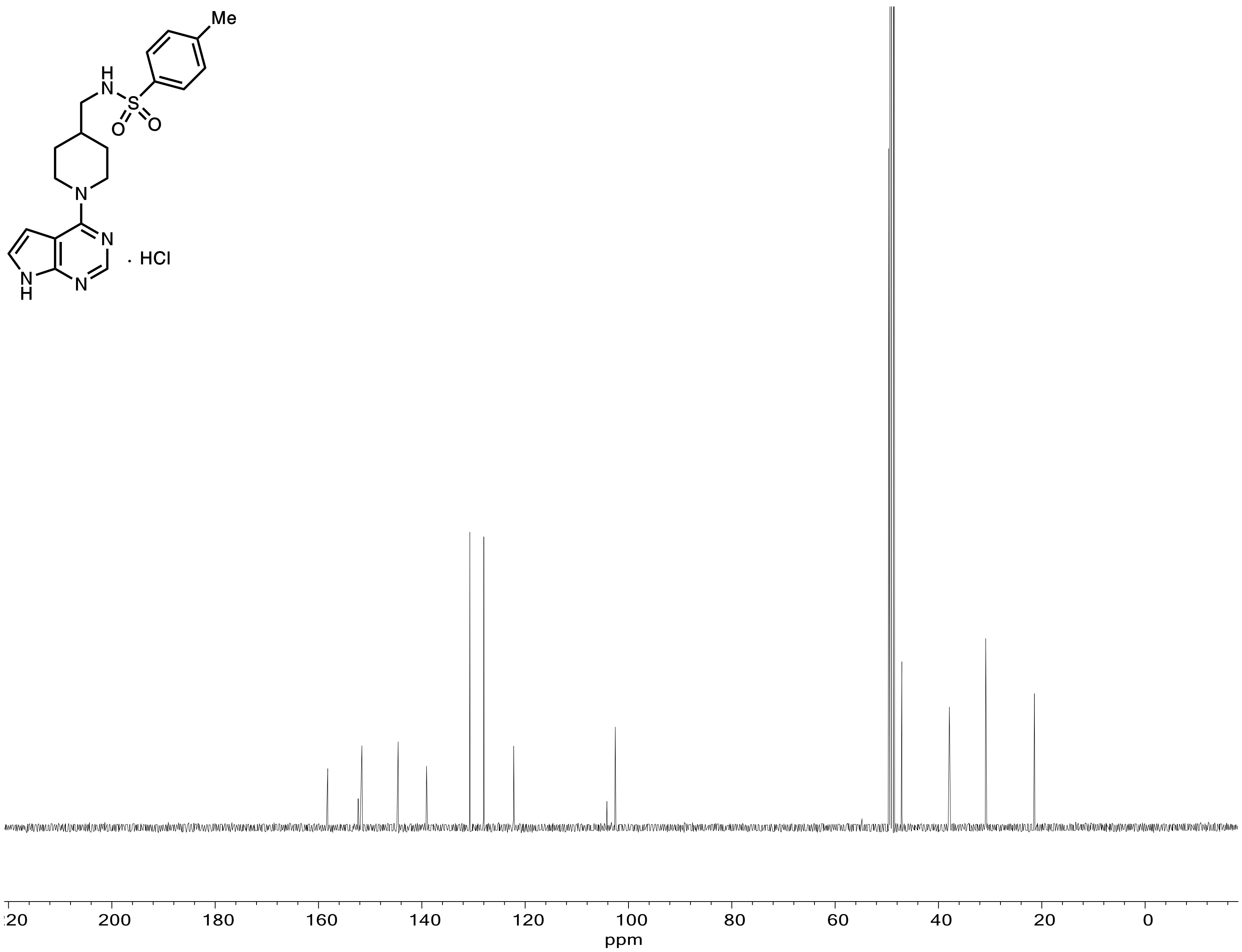

^{13}C NMR (101 MHz, MeOD) of compound **20b**.

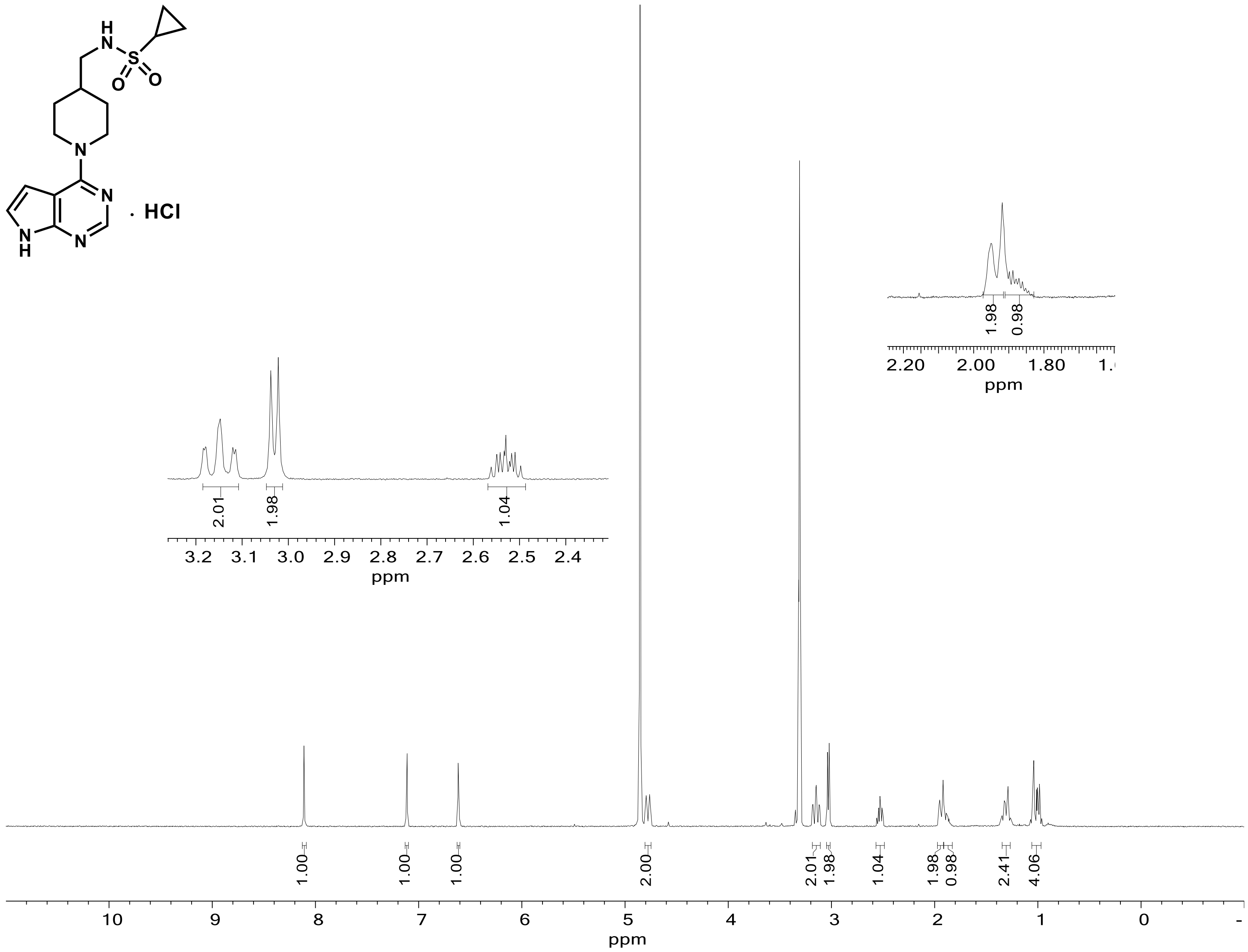

^{1}H NMR (400 MHz, MeOD) of compound **20c**.

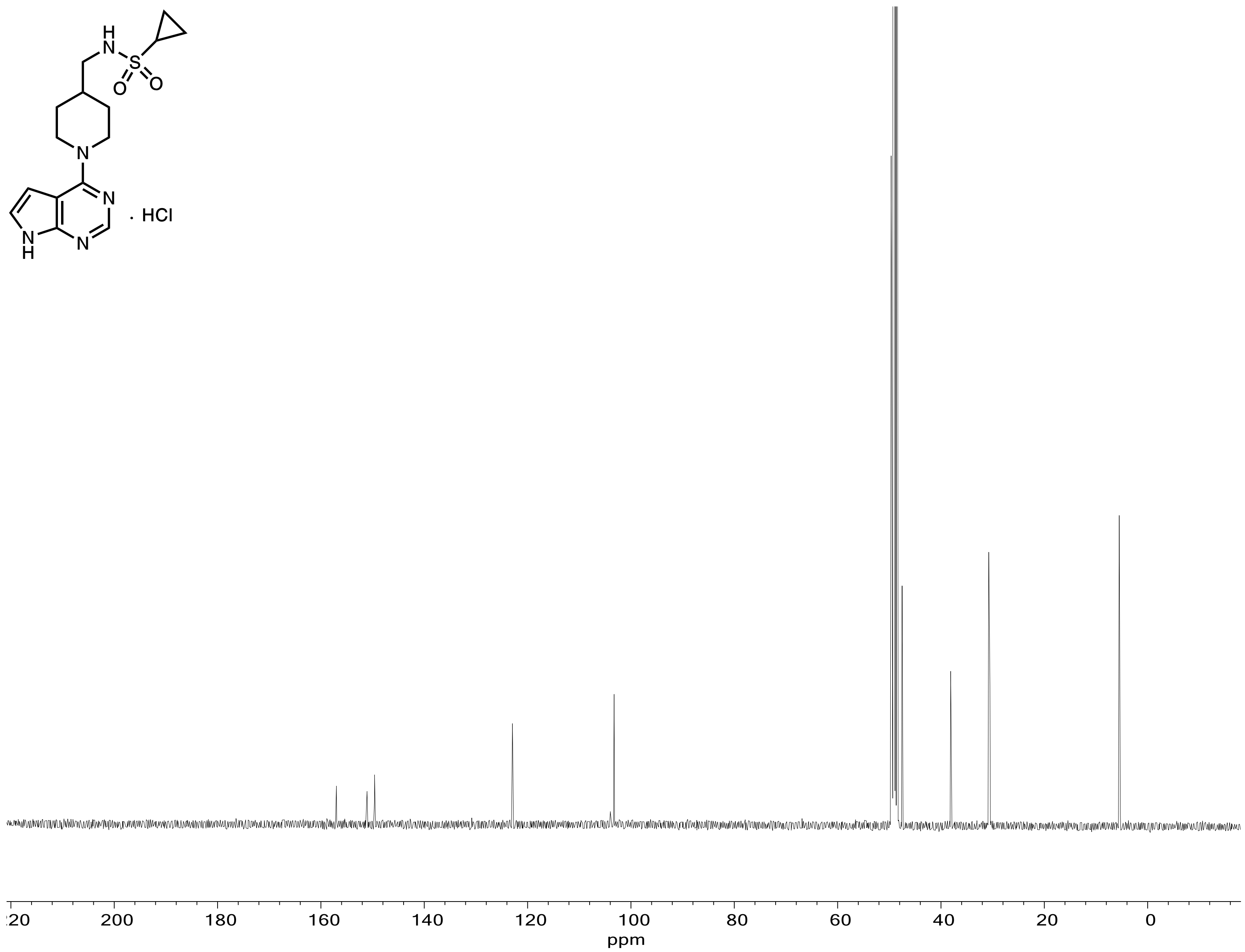

^{13}C NMR (101 MHz, MeOD) of compound **20c**.

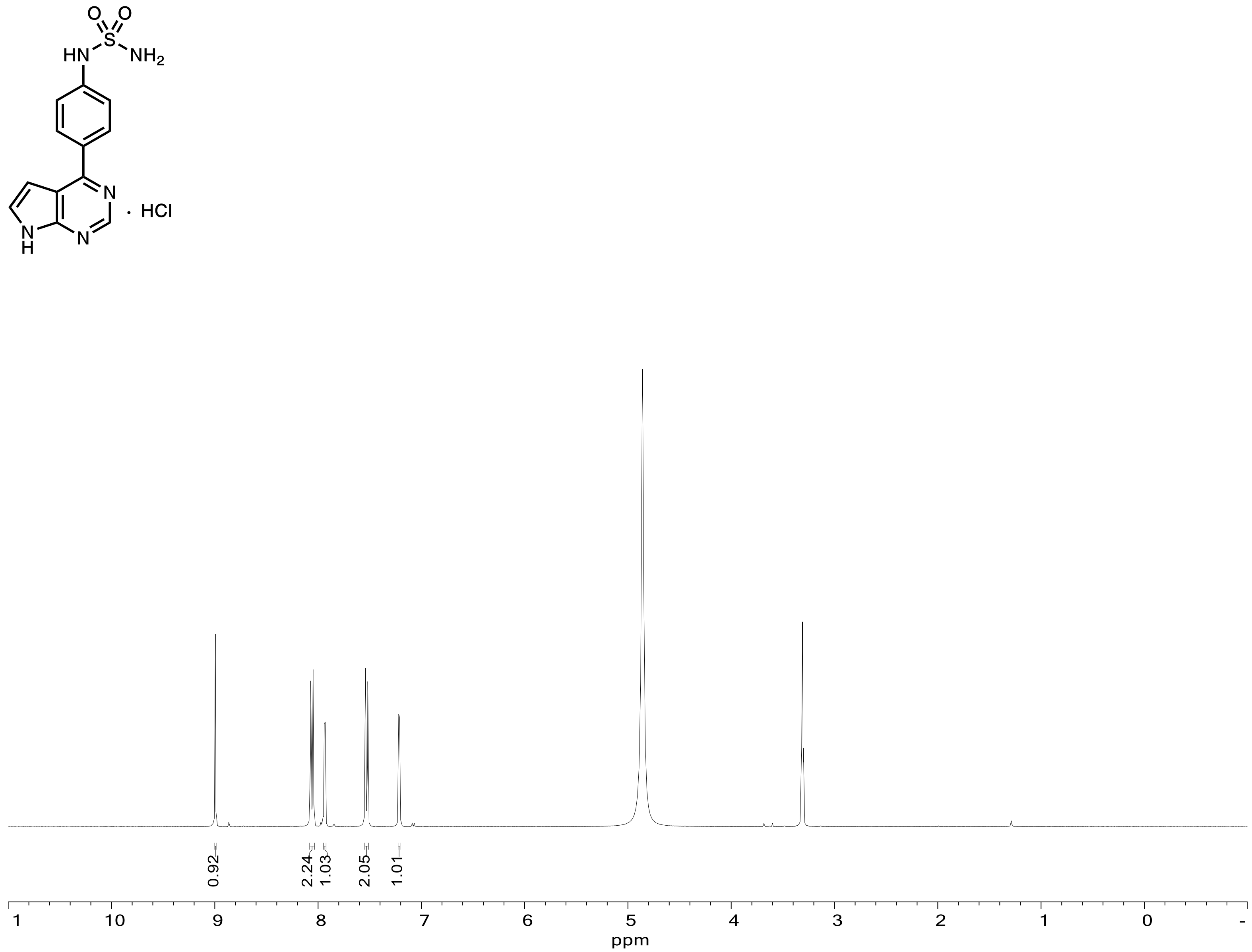

^{1}H NMR (400 MHz, MeOD) of compound **25a**.

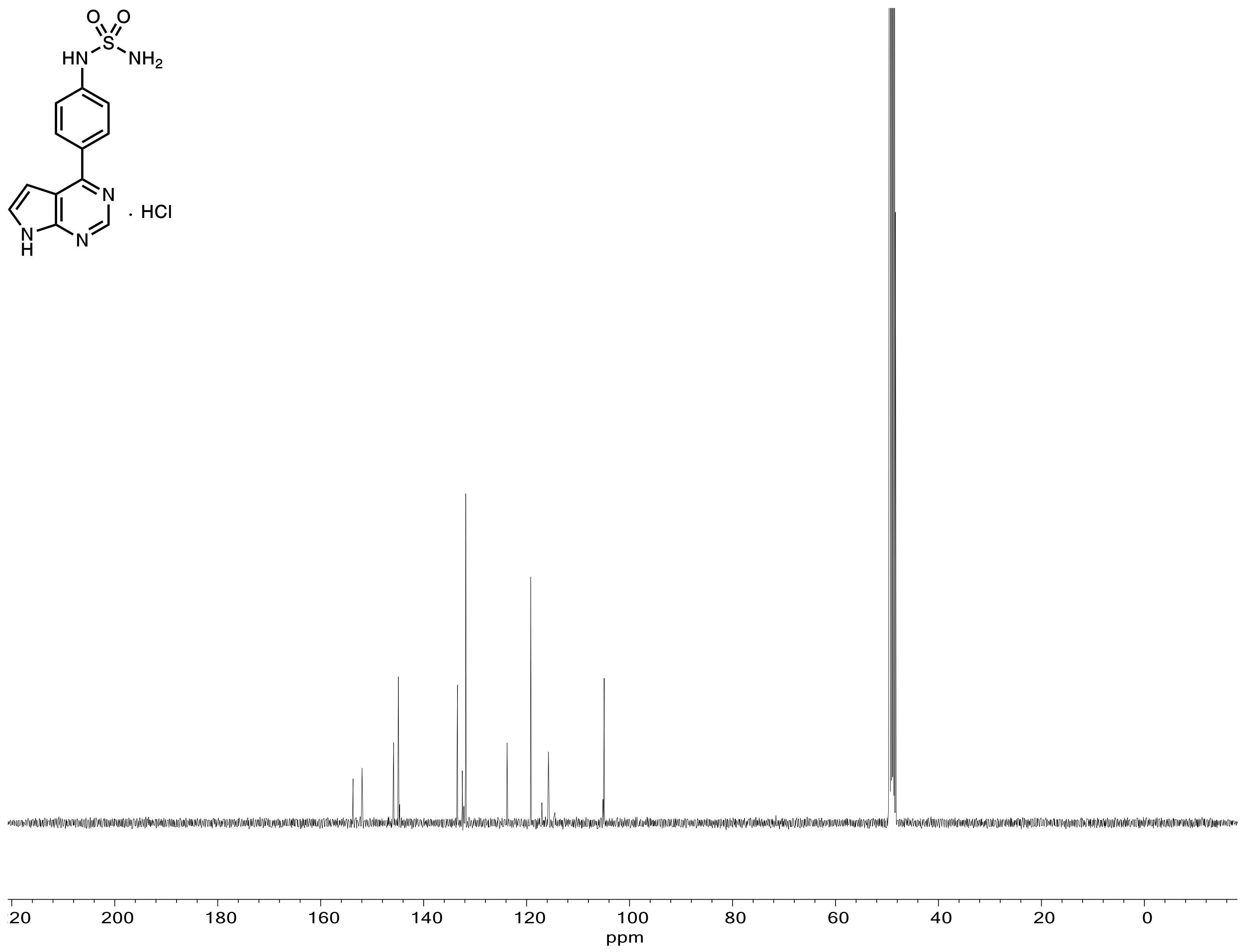

^{13}C NMR (101 MHz, MeOD) of compound **25a**.

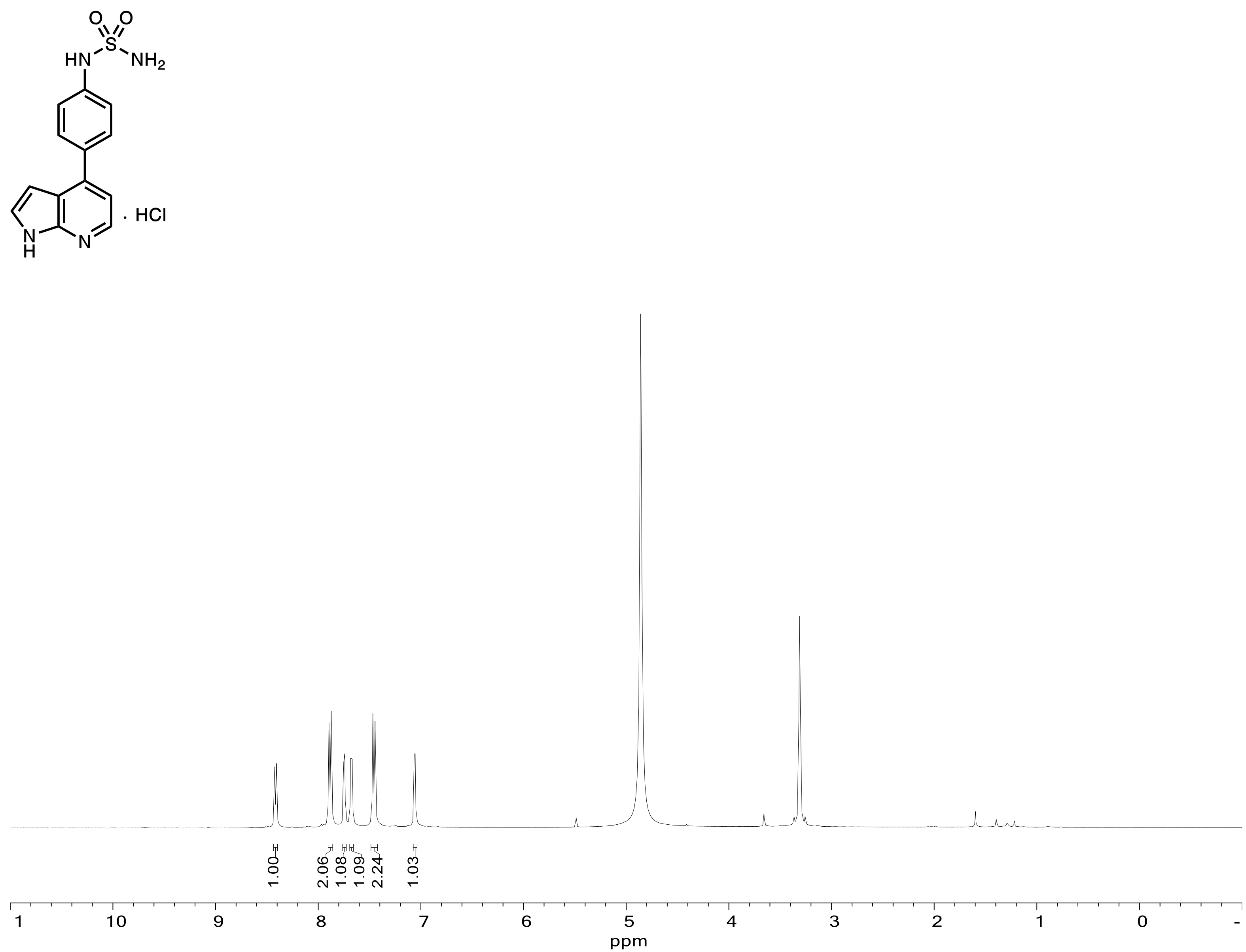

^{1}H NMR (400 MHz, MeOD) of compound **25b**.

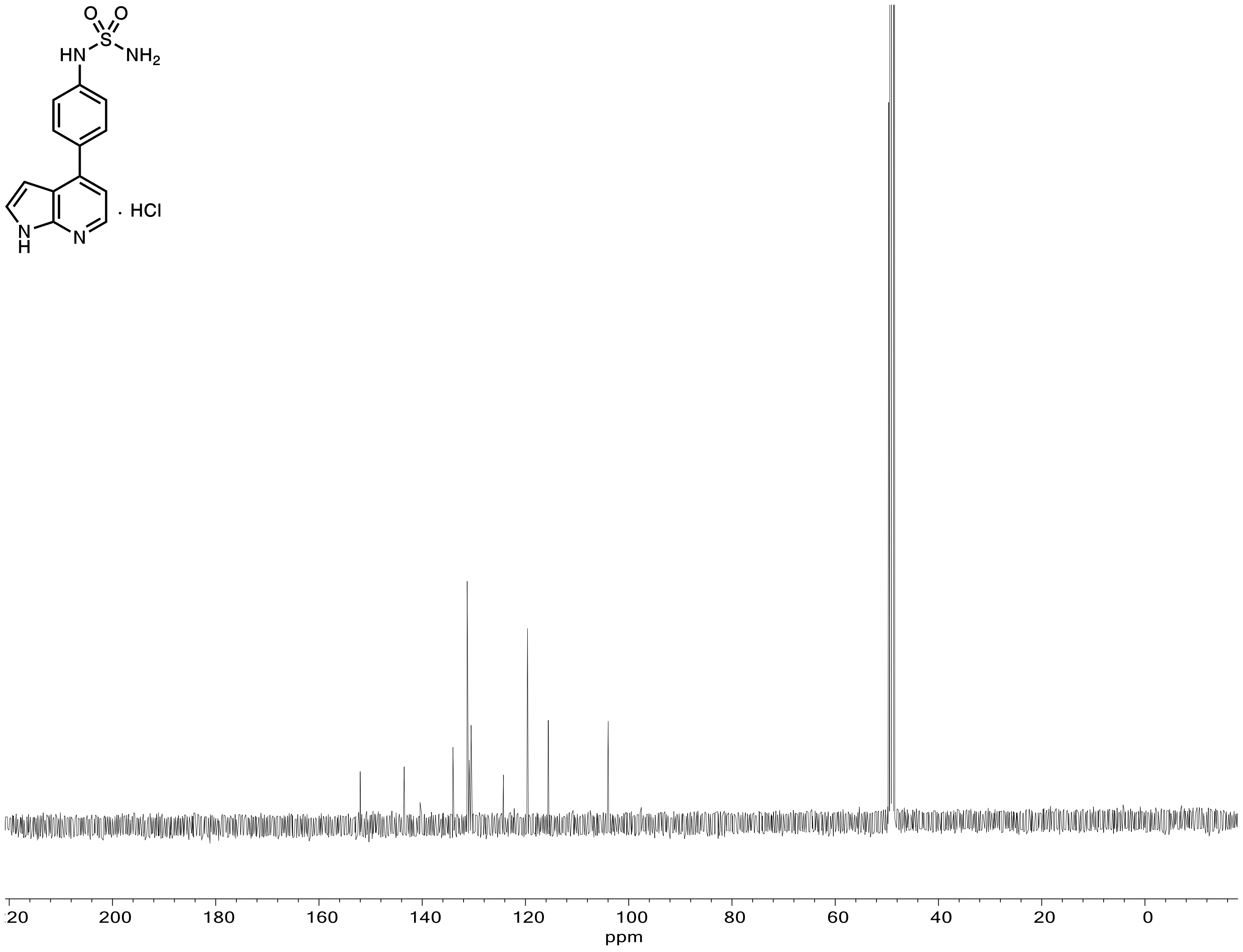

^{13}C NMR (101 MHz, MeOD) of compound **25b**.

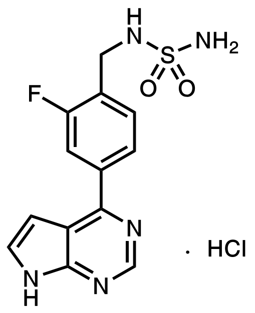

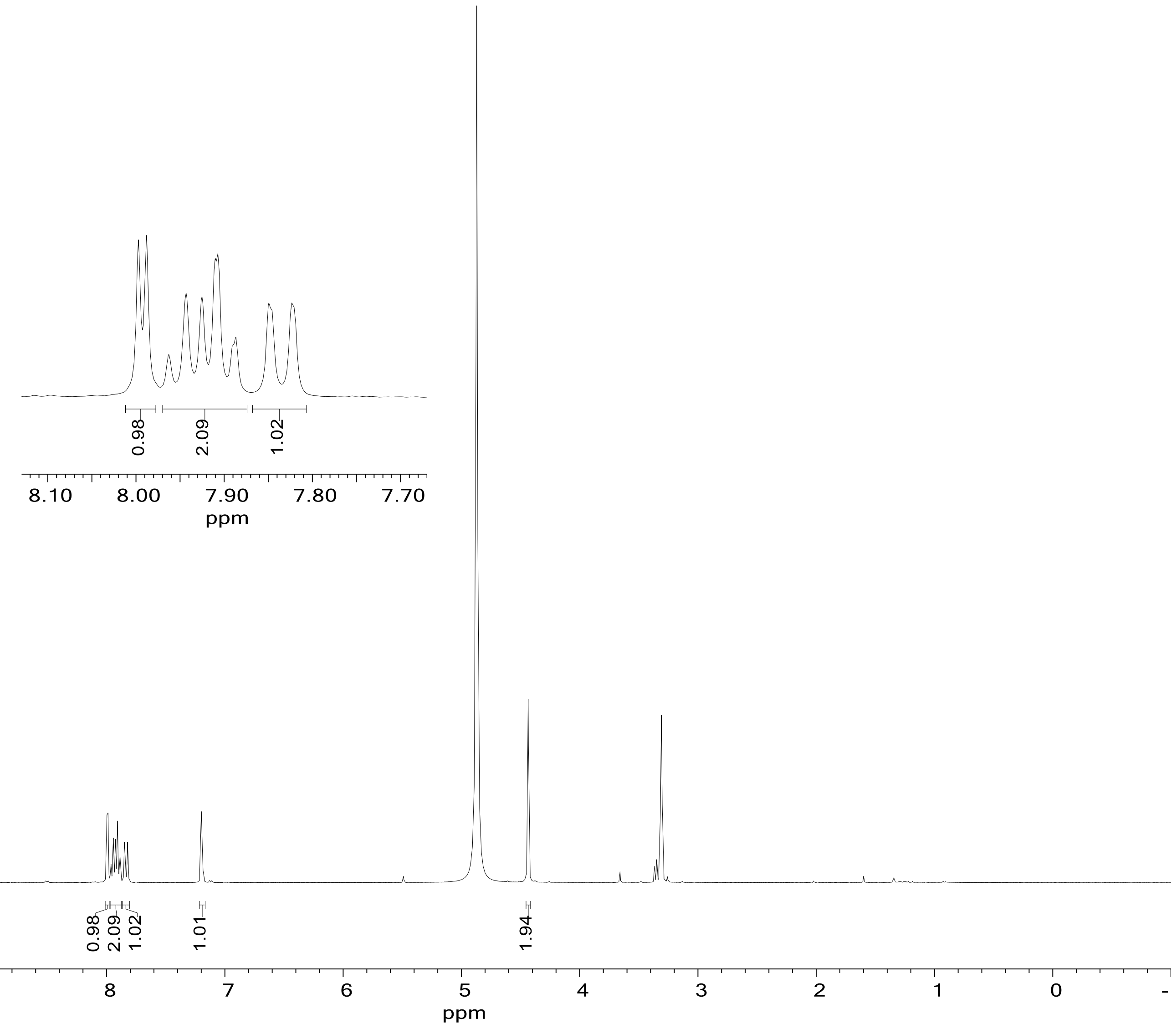

^{1}H NMR (400 MHz, MeOD) of compound **25c**.

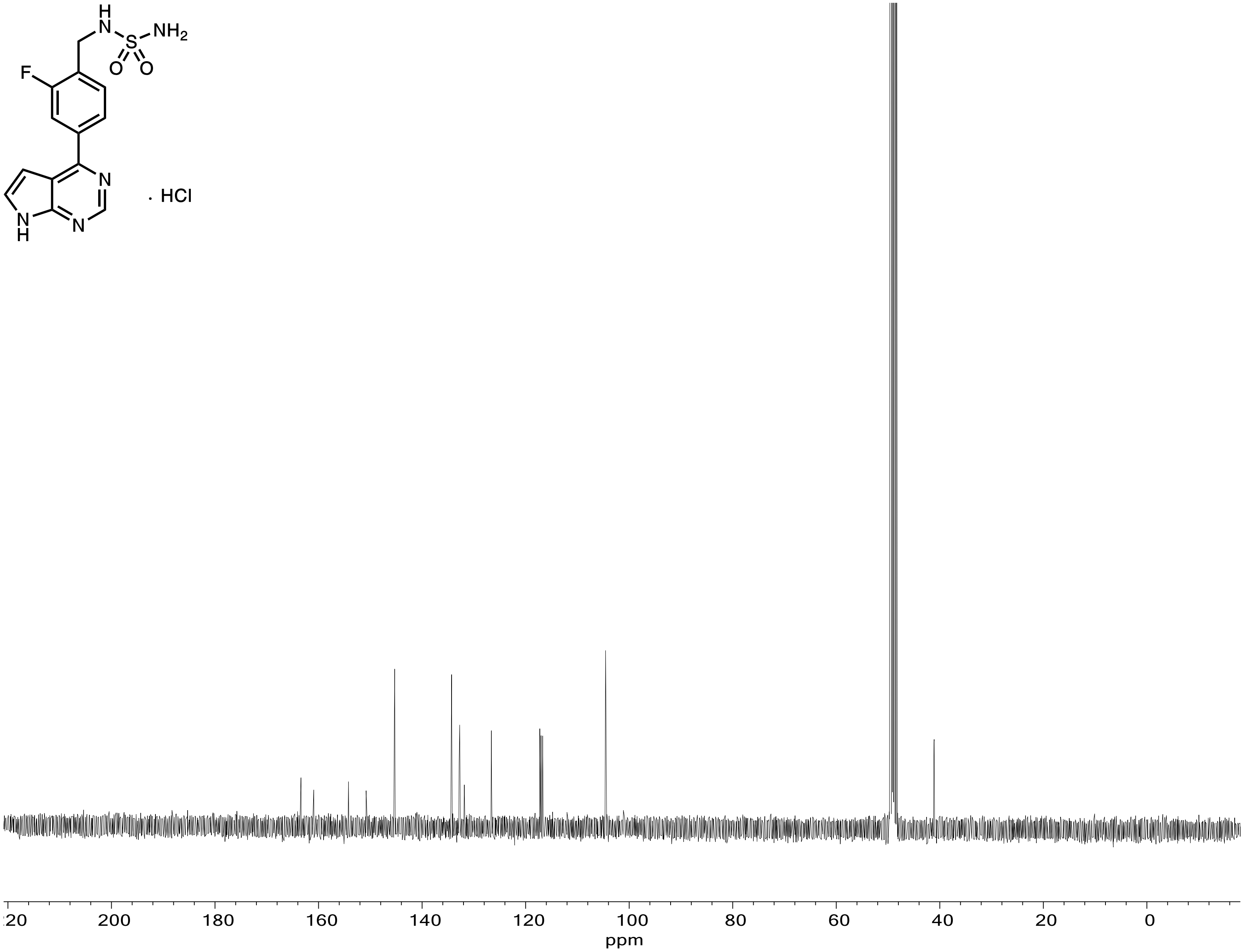

^{13}C NMR (101 MHz, MeOD) of compound **25c**.

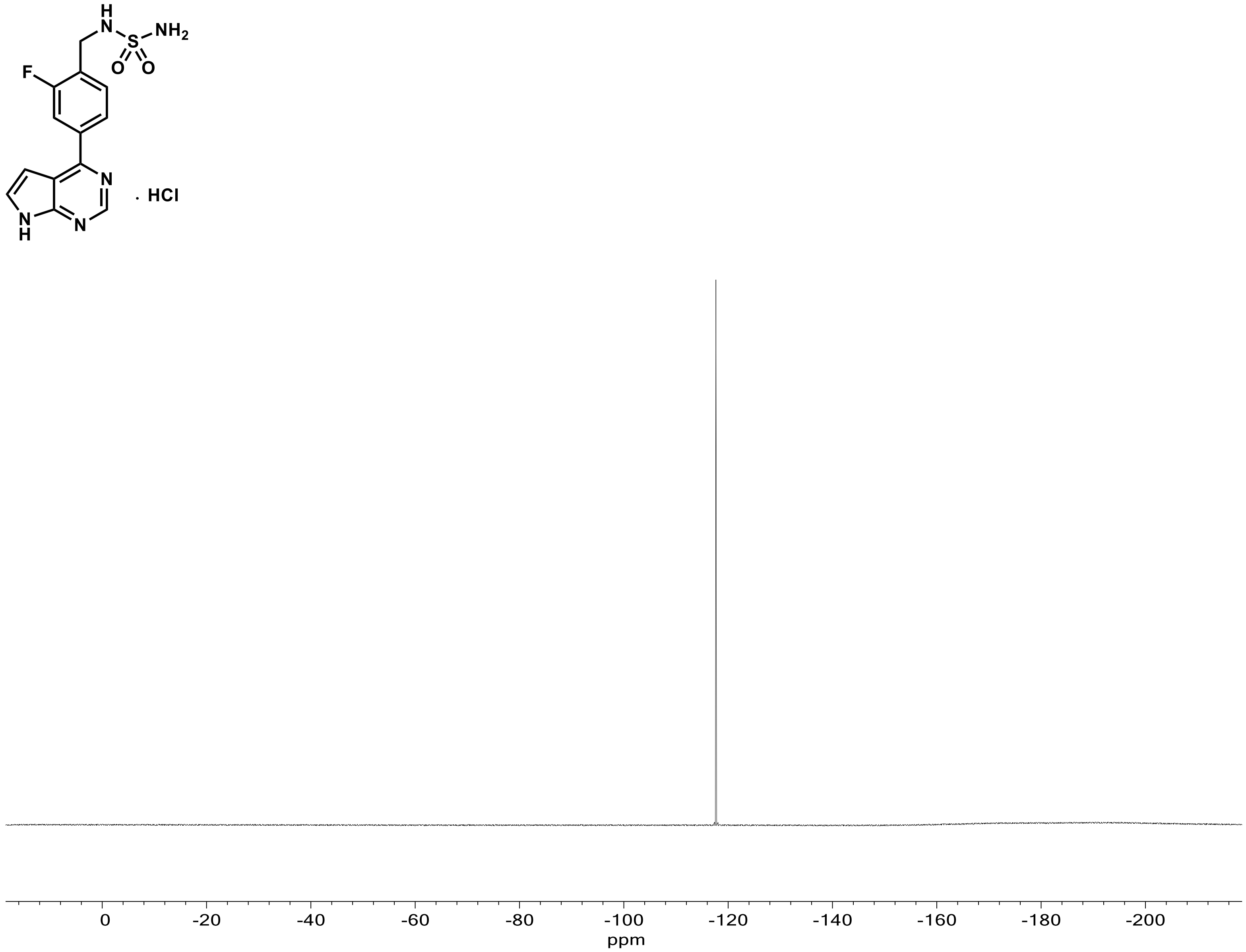

^{19}F NMR (377 MHz, MeOD) of compound **25c**.

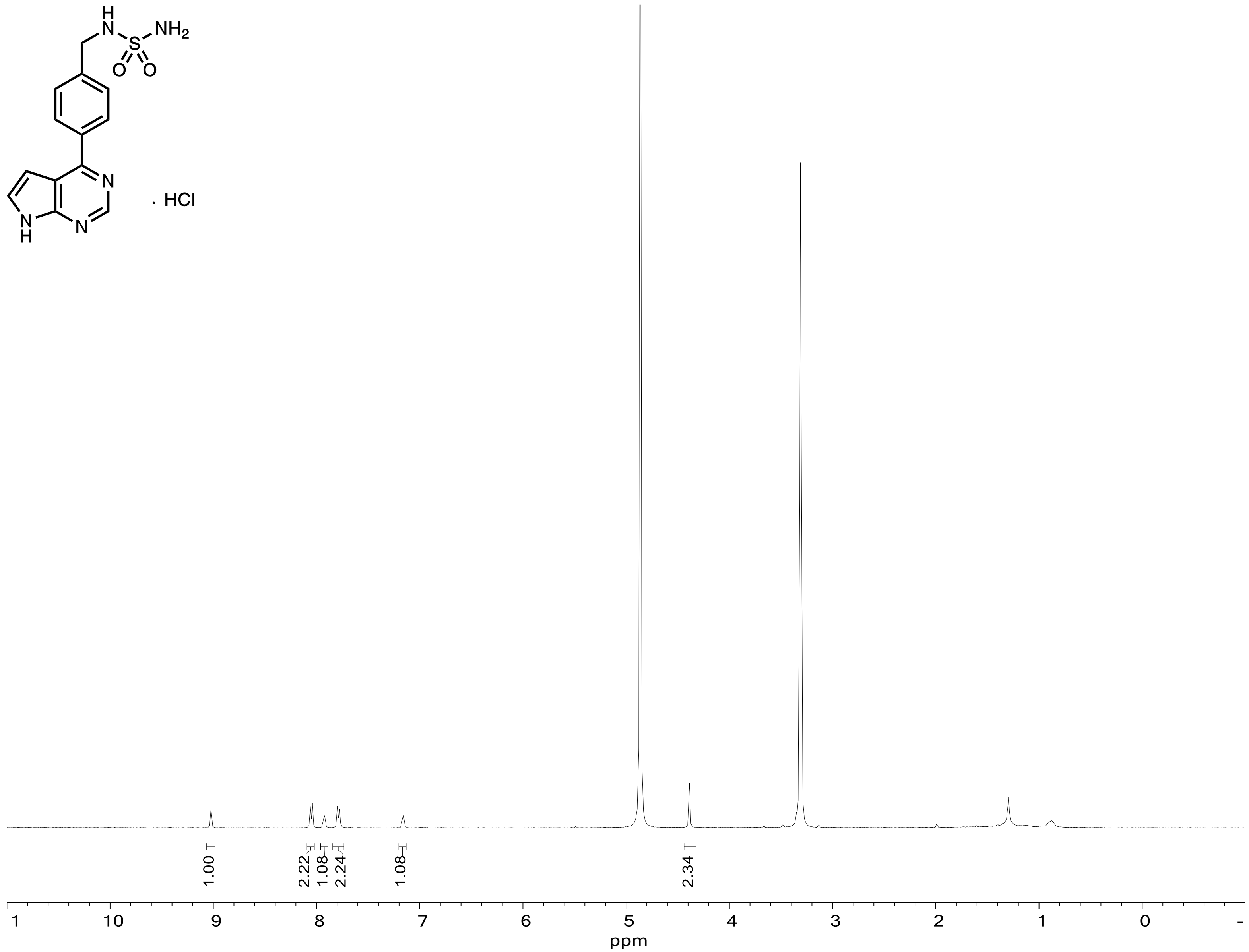

^{1}H NMR (400 MHz, MeOD) of compound **25d**.

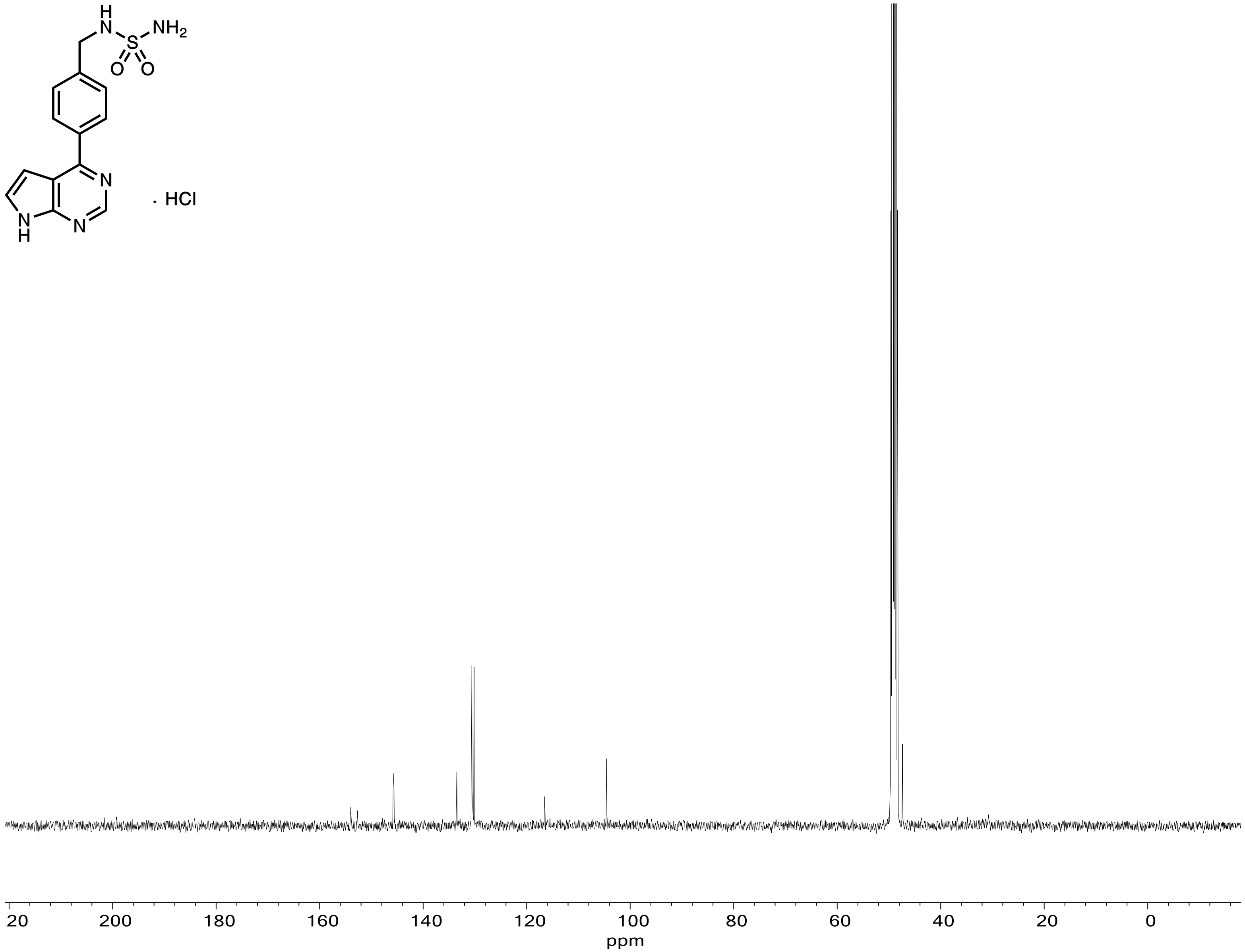

^{13}C NMR (101 MHz, MeOD) of compound **25d**.

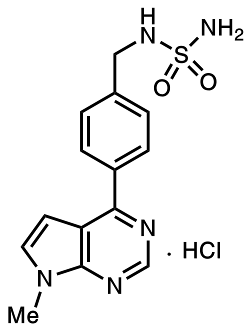

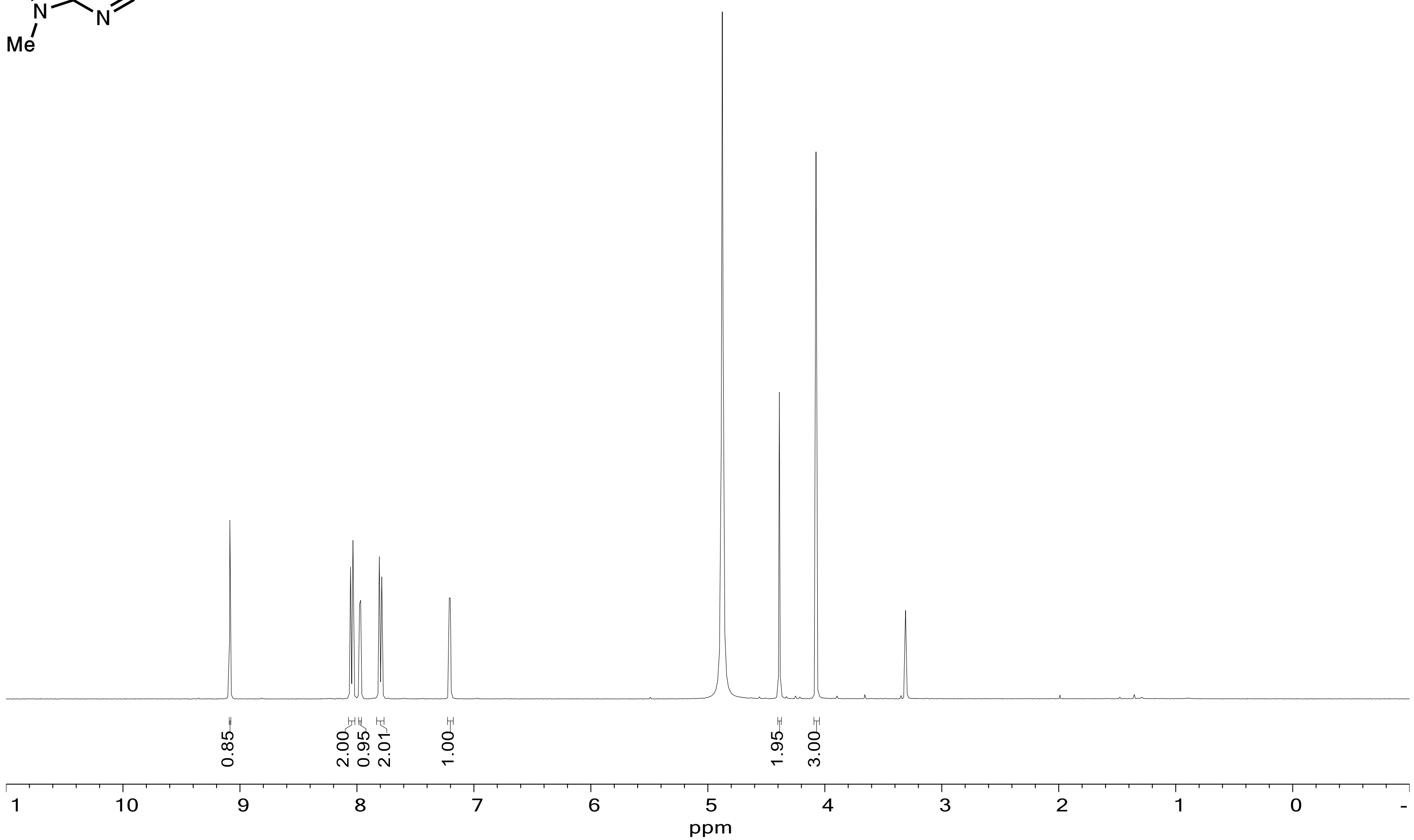

^{1}H NMR (400 MHz, MeOD) of compound **25e**.

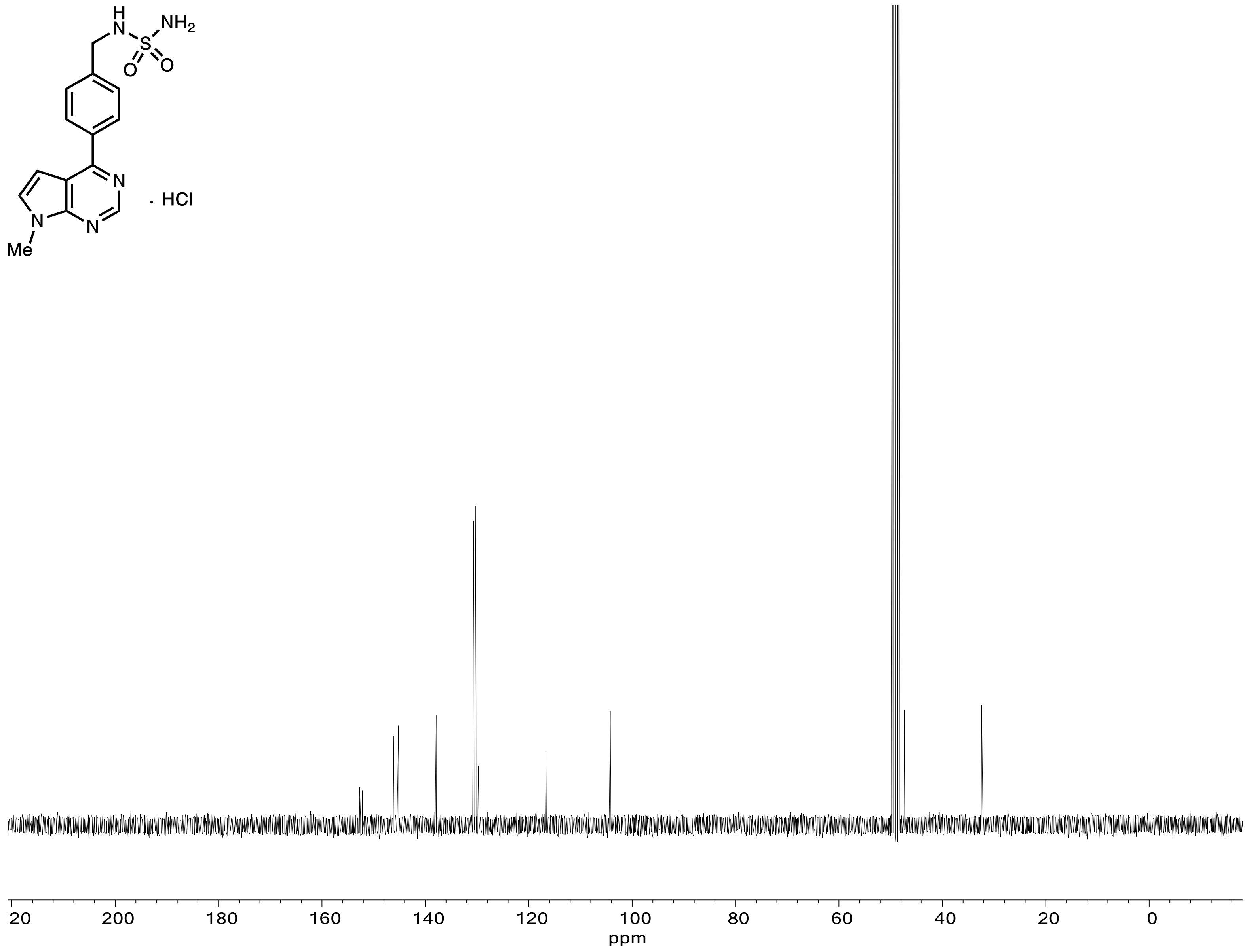

^{13}C NMR (101 MHz, MeOD) of compound **25e**.

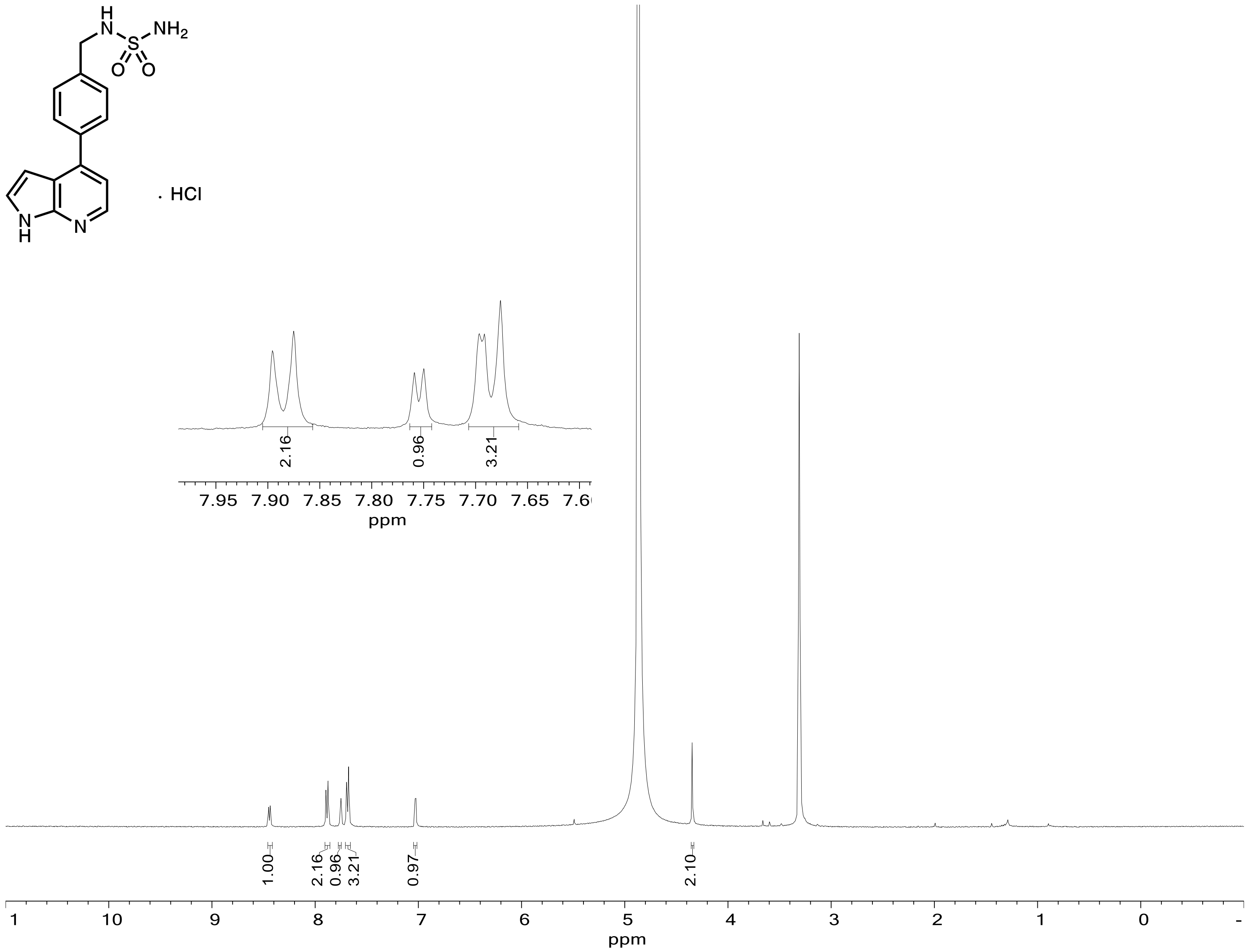

^{1}H NMR (400 MHz, MeOD) of compound **25f**.

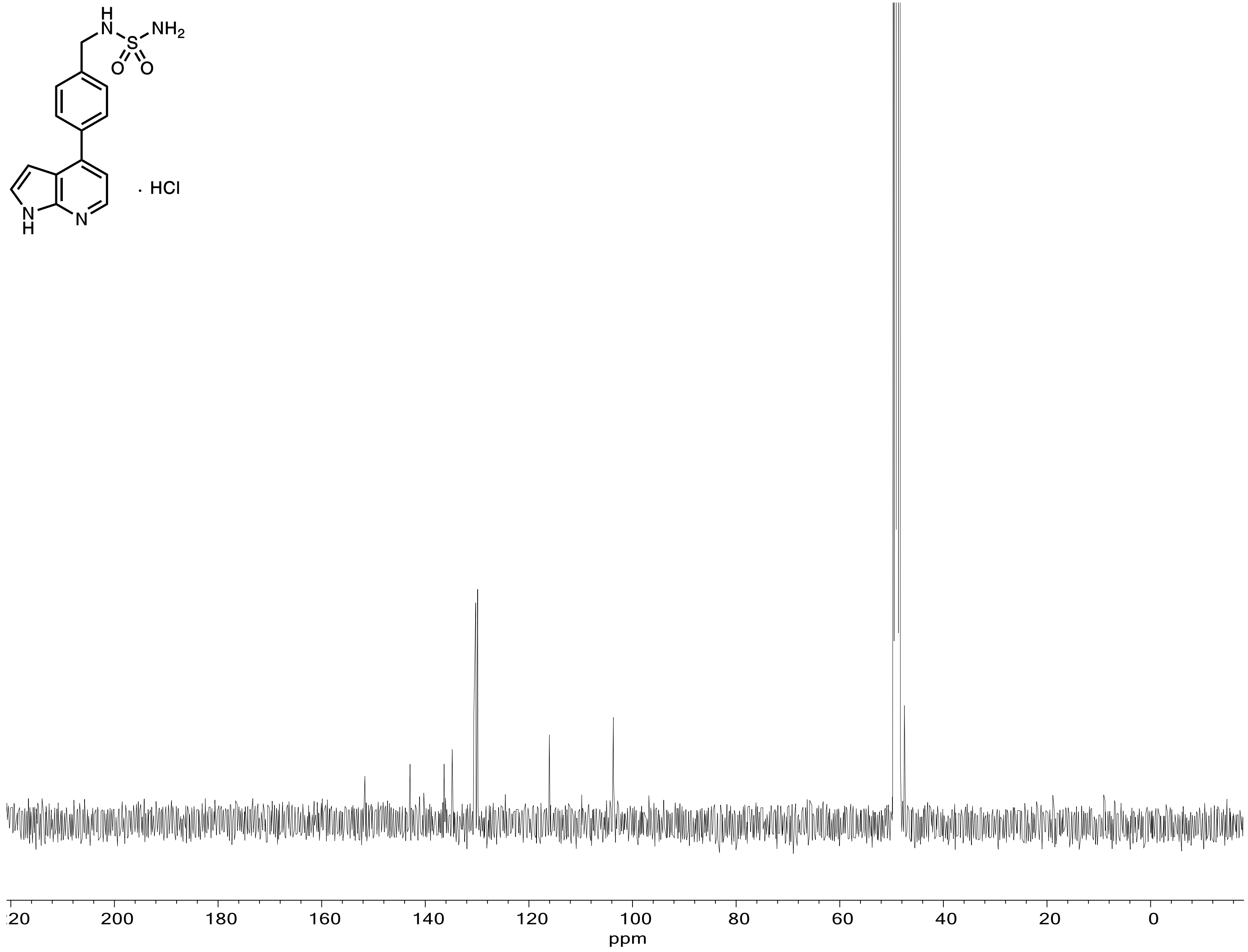

^{13}C NMR (101 MHz, MeOD) of compound **25f**.

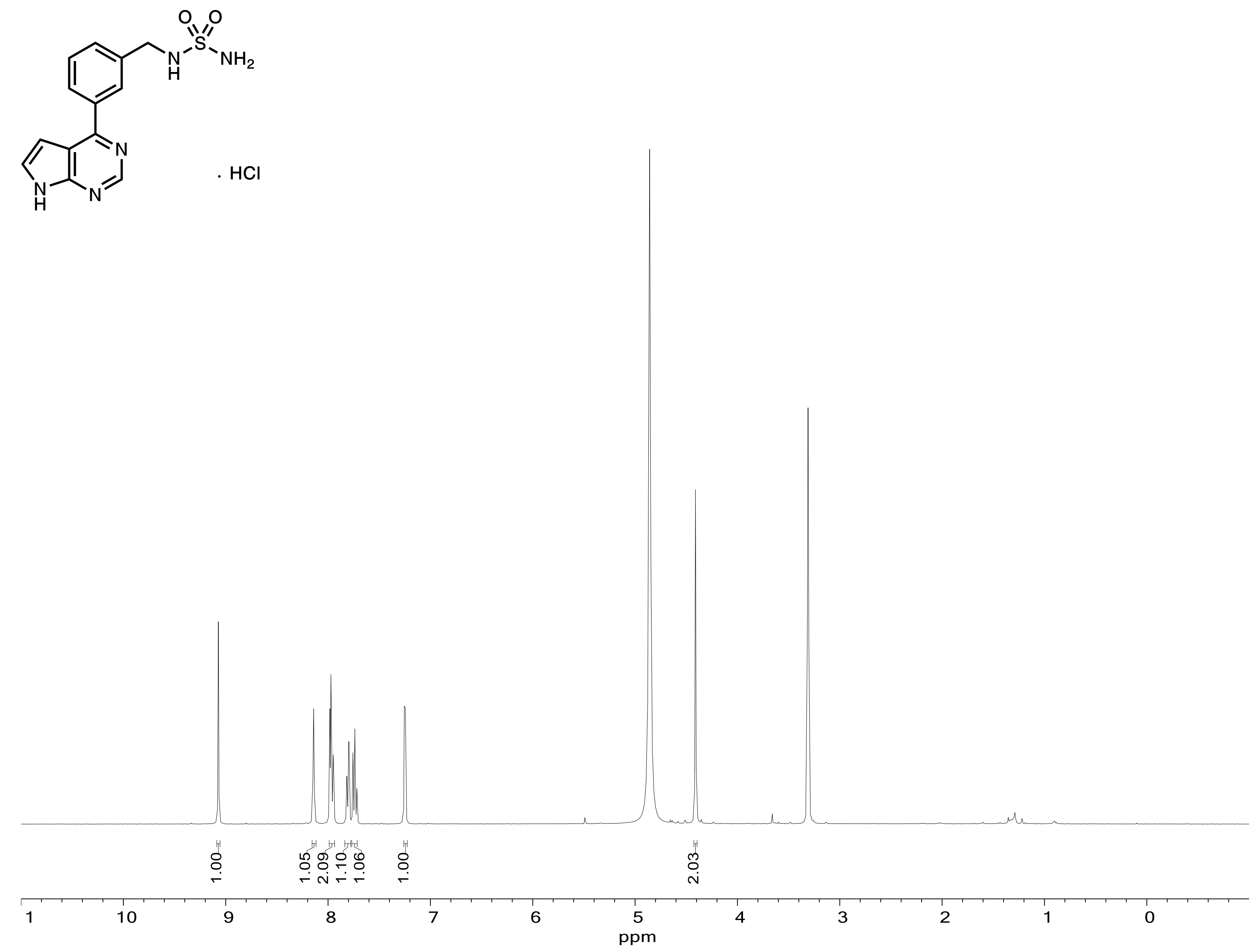

^{1}H NMR (400 MHz, MeOD) of compound **25g**.

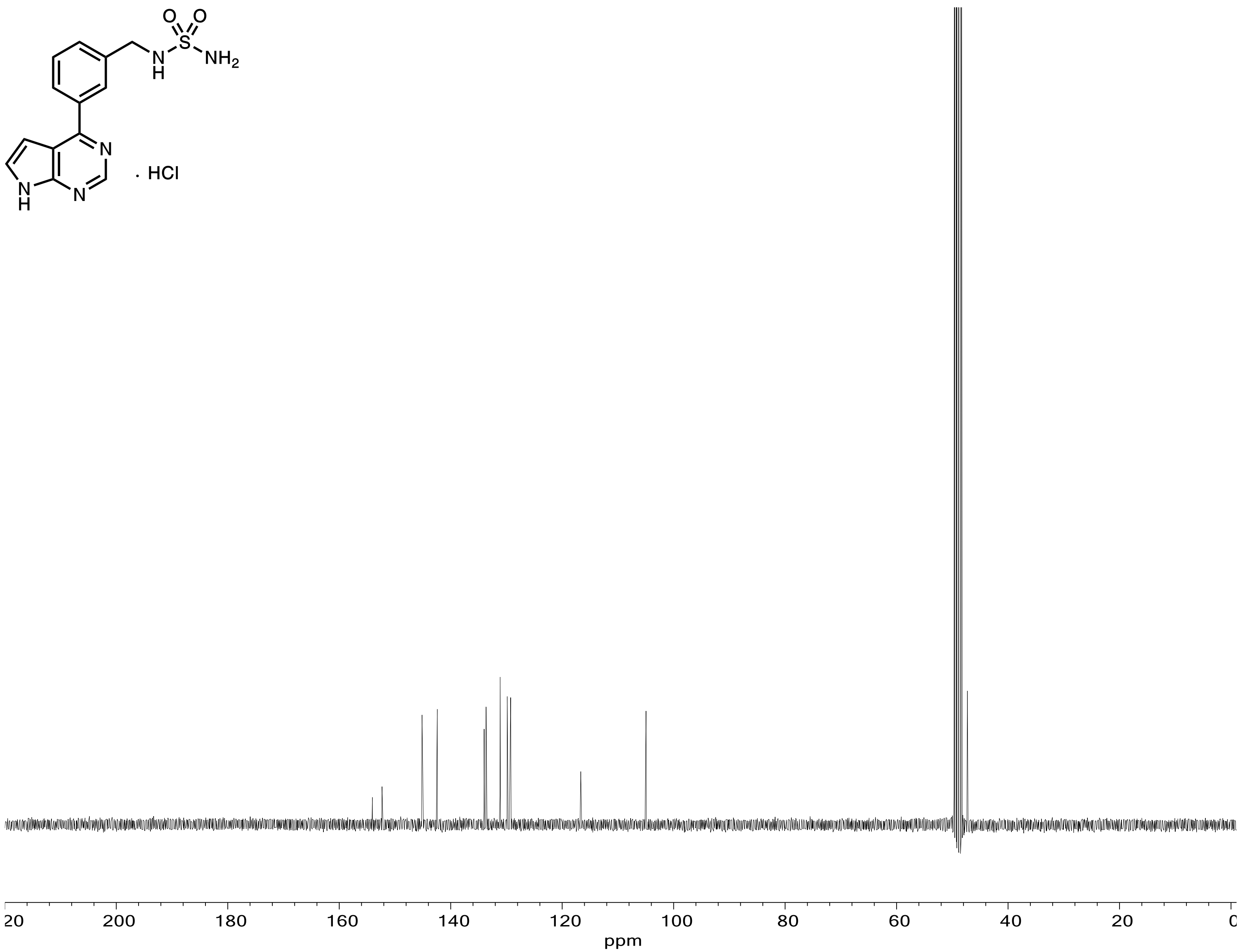

^{13}C NMR (101 MHz, MeOD) of compound **25g**.

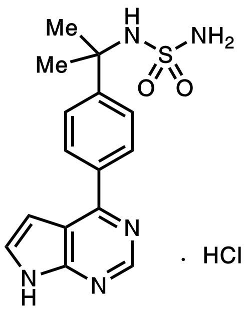

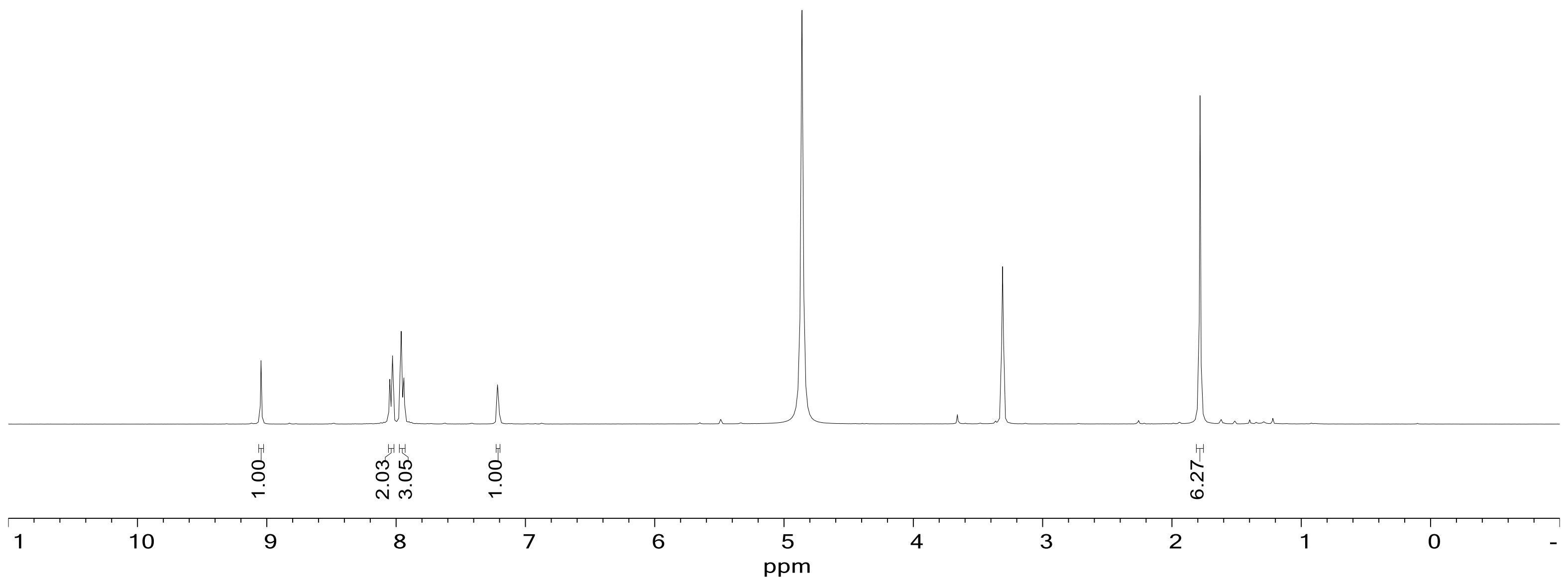

^{1}H NMR (400 MHz, MeOD) of compound **25h**.

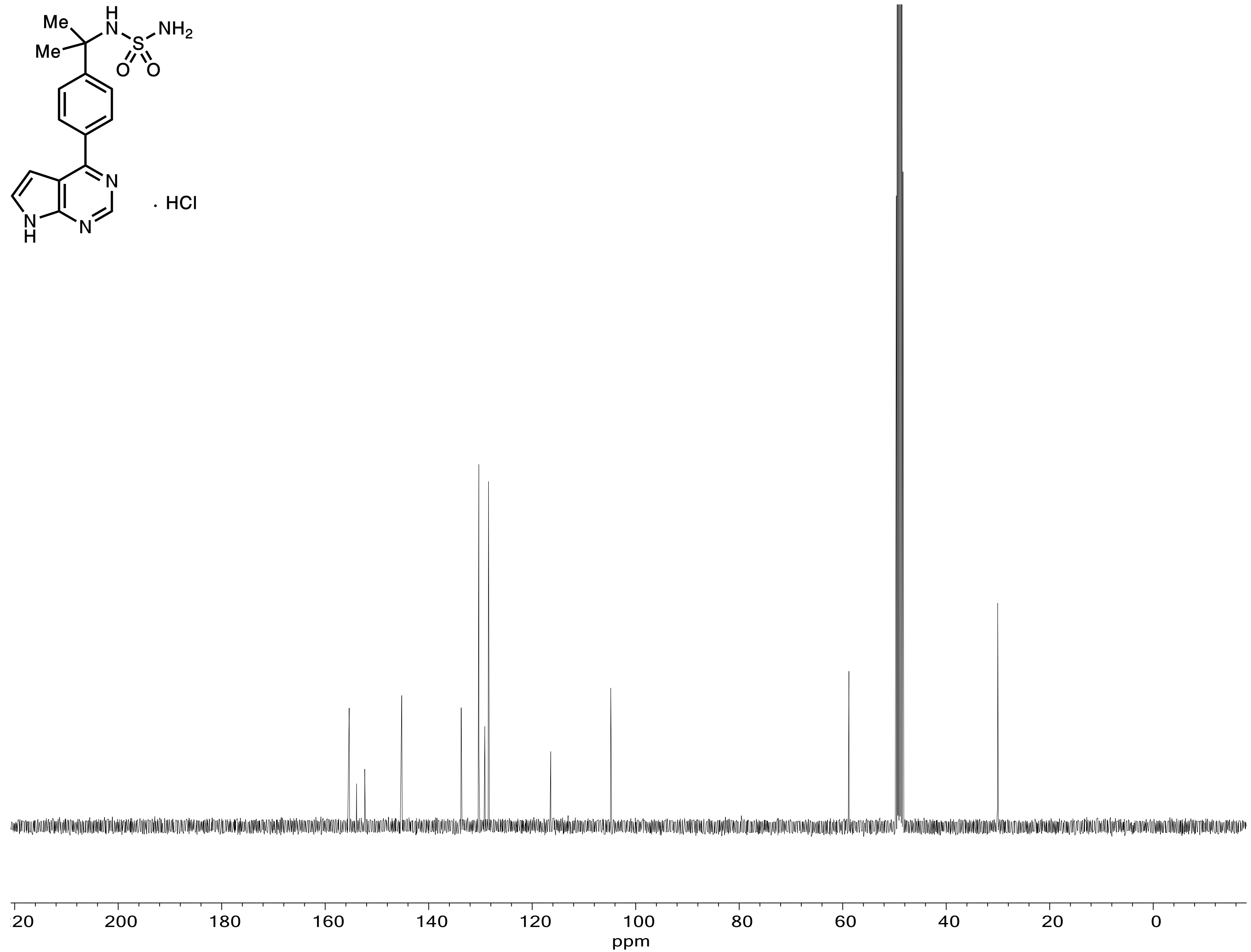

^{13}C NMR (101 MHz, MeOD) of compound **25h**.

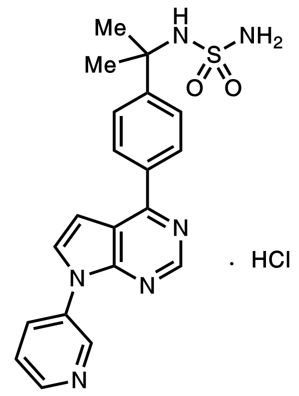

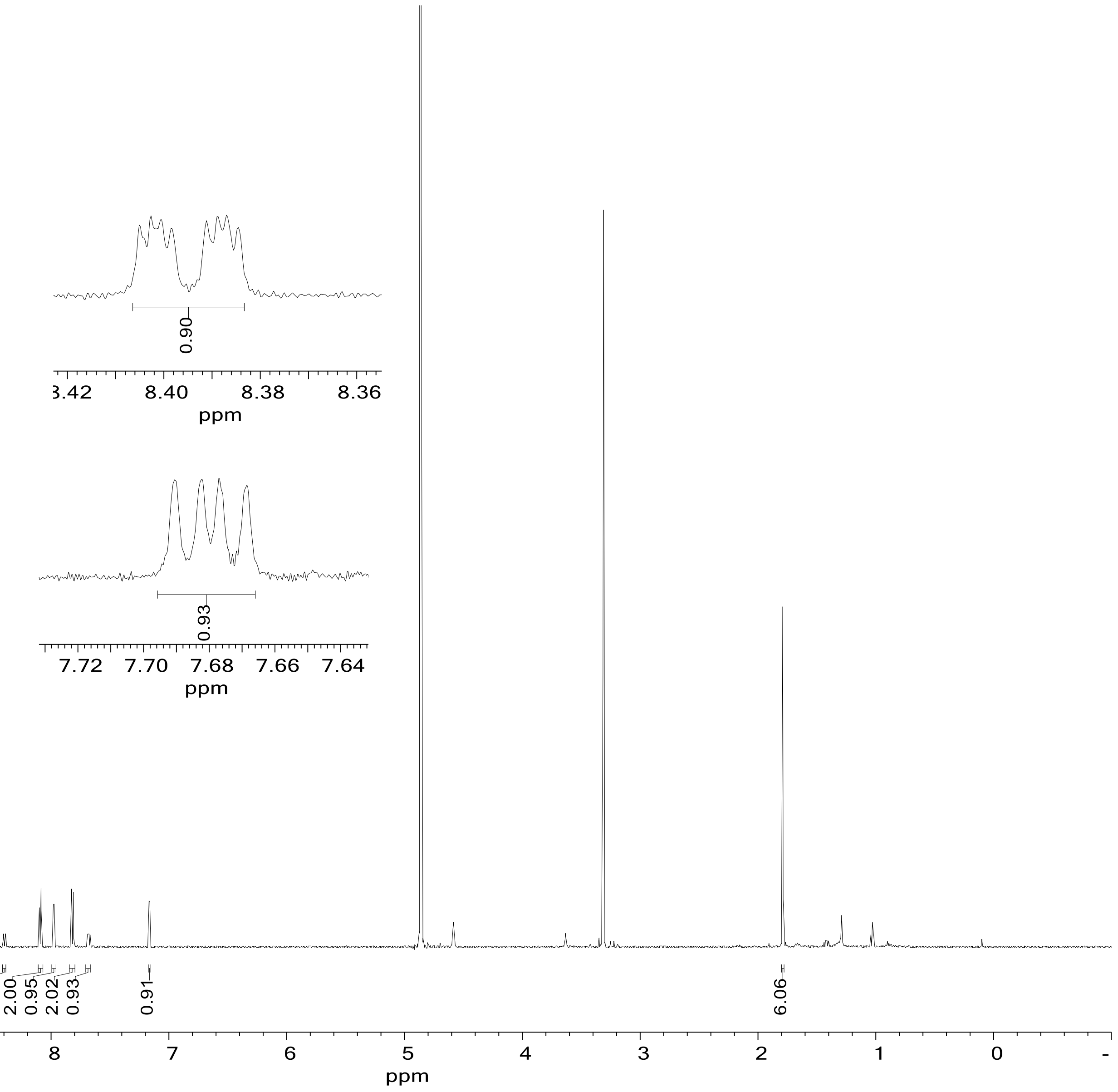

^1H NMR (600 MHz, MeOD) of compound **25i**.

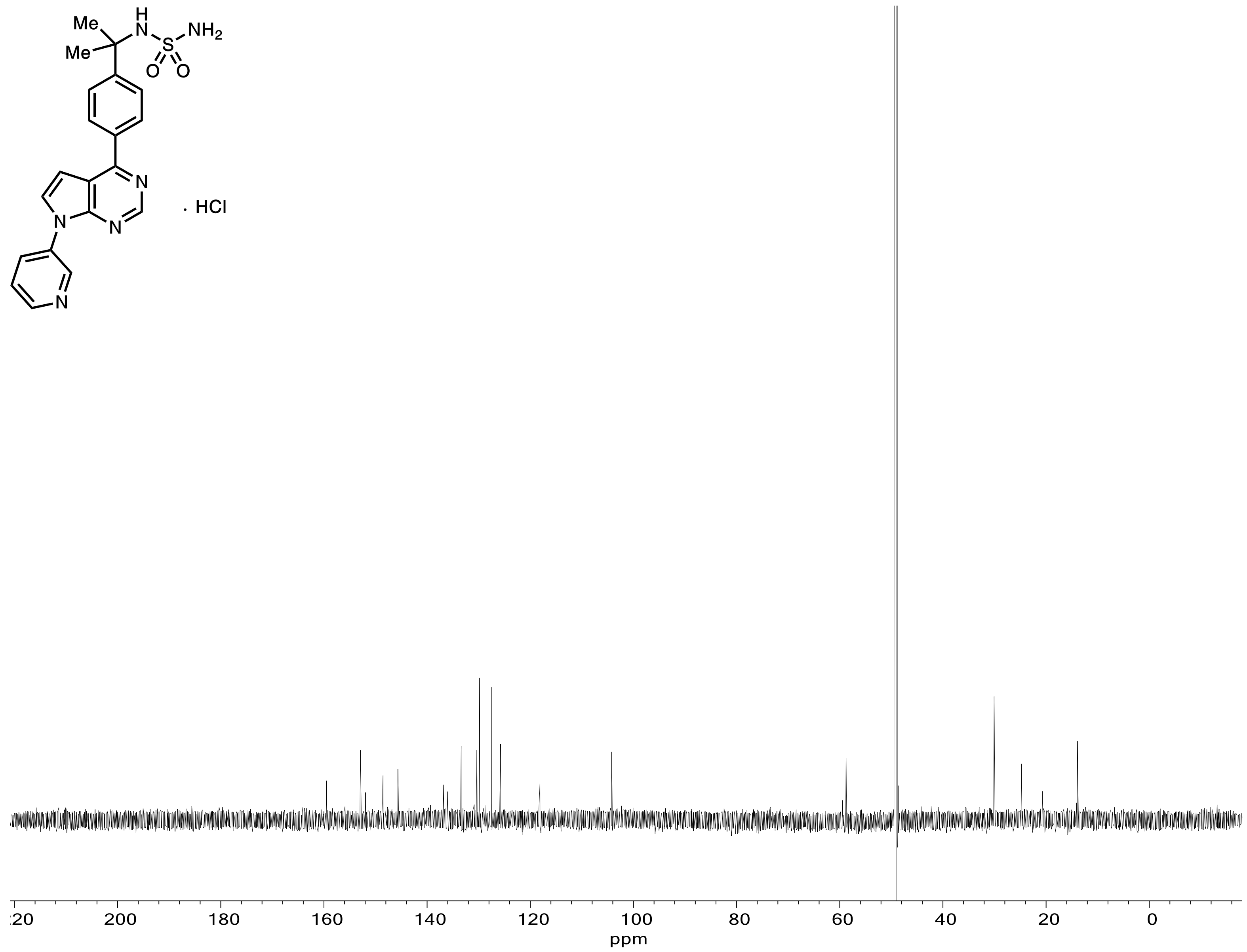

^{13}C NMR (201 MHz, MeOD) of compound **25i**.

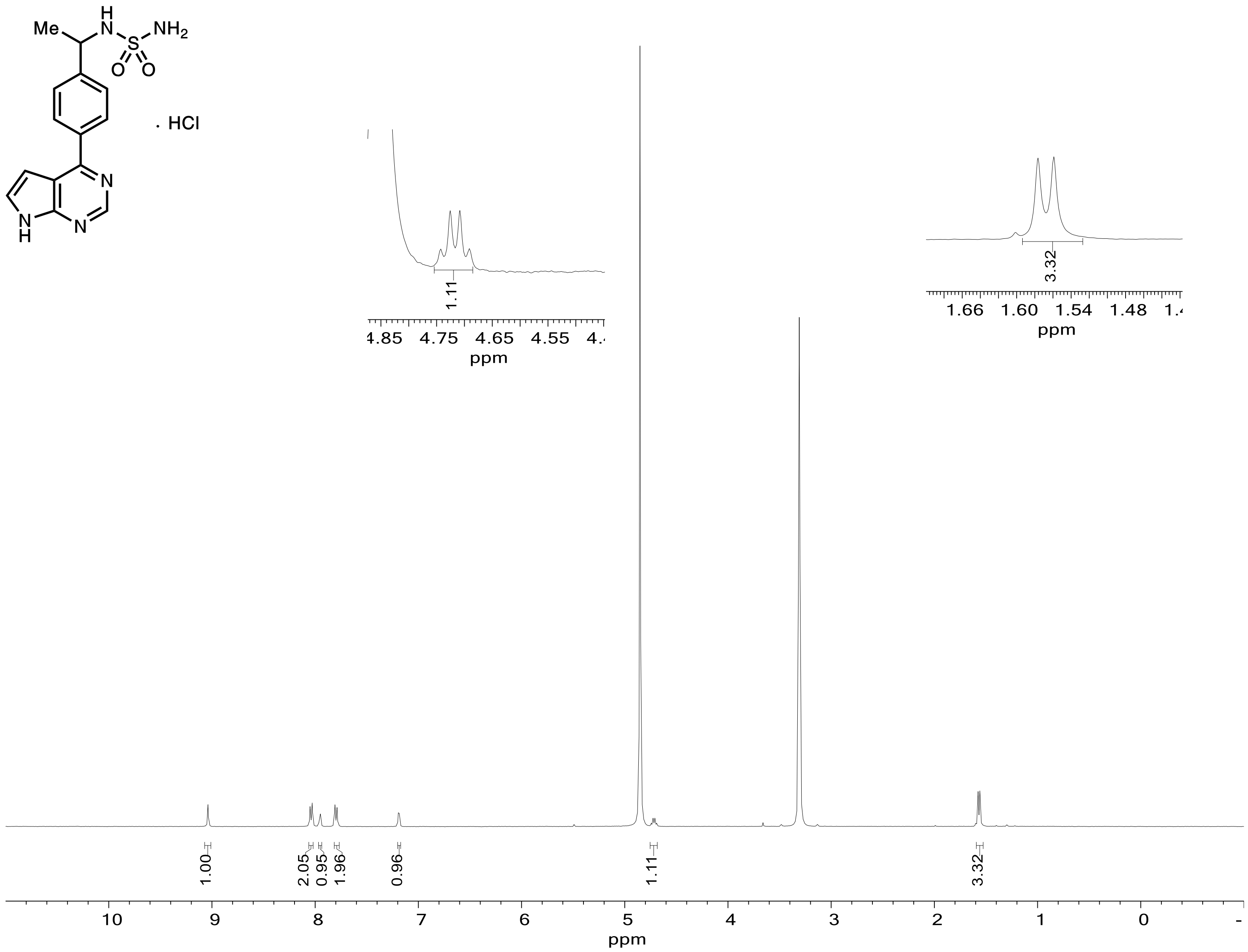

^{1}H NMR (400 MHz, MeOD) of compound **25j**.

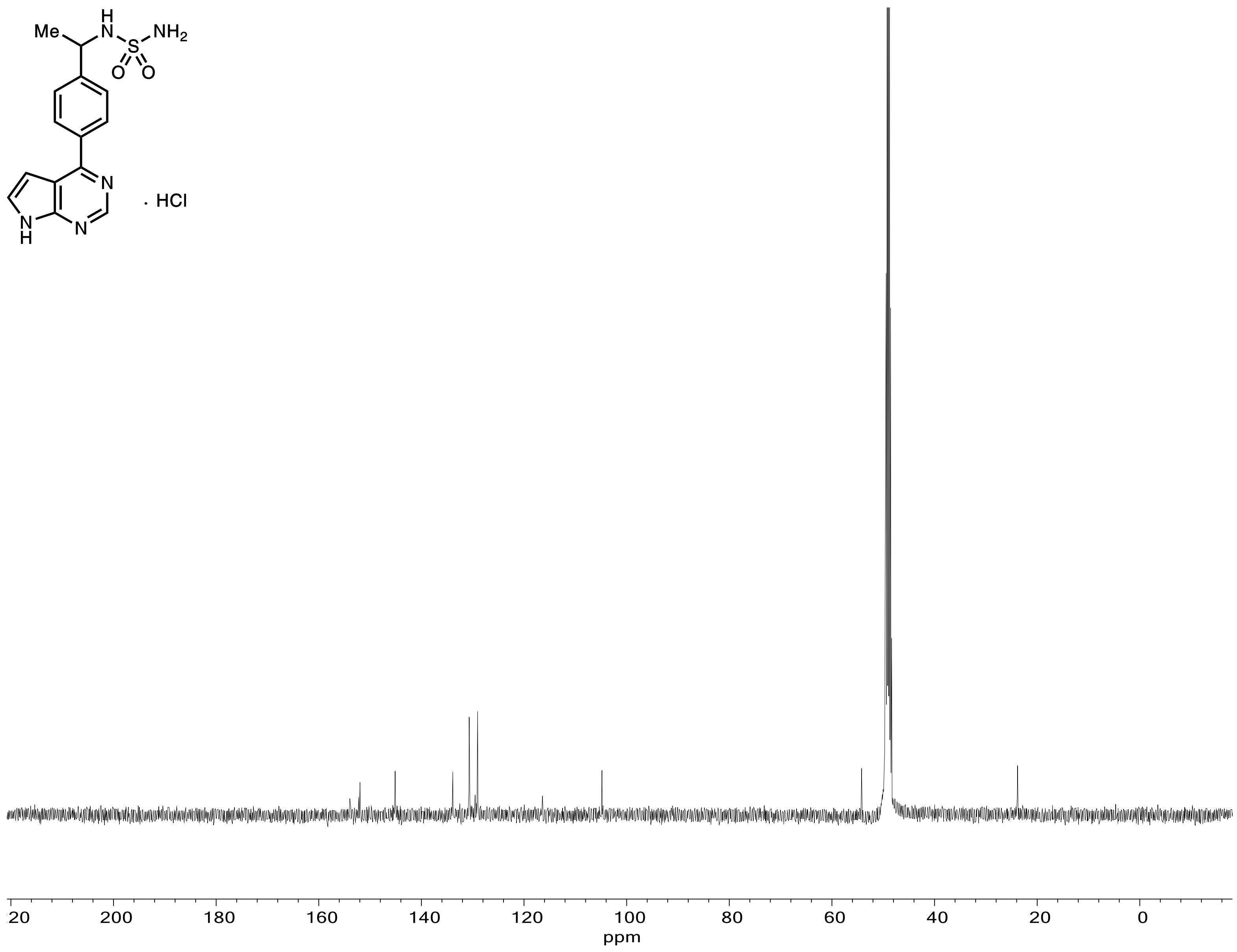

^{13}C NMR (101 MHz, MeOD) of compound **25j**.

HPLC trace of compound **18a**

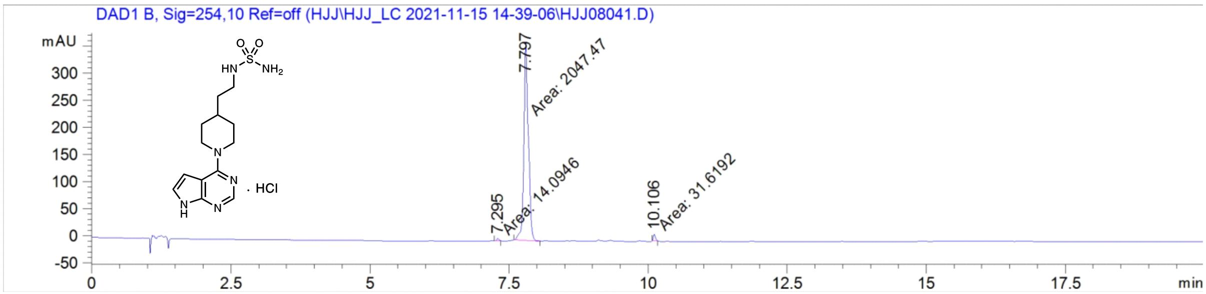

Peak #	RetTime [min]	Type	Width [min]	Area [mAU*s]	Height [mAU]	Area %
1	7.295	MM	0.0489	14.09463	4.79986	0.6734
2	7.797	MM	0.0936	2047.46851	364.63367	97.8161
3	10.106	MM	0.0434	31.61922	12.15347	1.5106
Totals :				2093.18236	381.58700	

HPLC trace of compound **18b**

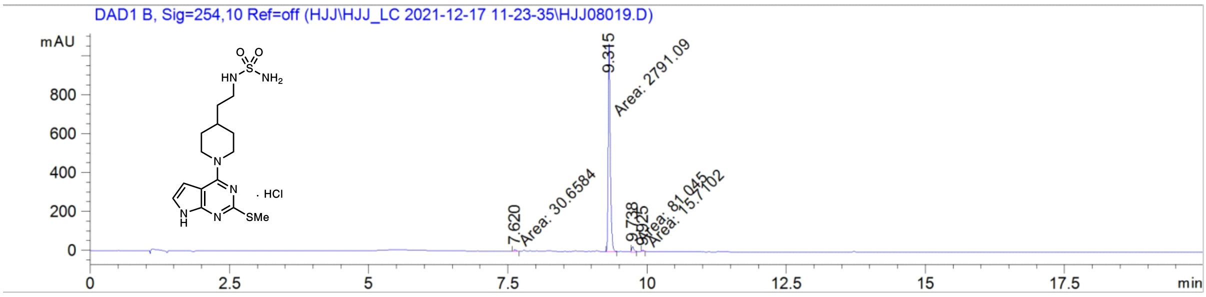

Peak #	RetTime [min]	Type	Width [min]	Area [mAU*s]	Height [mAU]	Area %
1	7.620	MM	0.0541	30.65840	9.45219	1.0505
2	9.315	MF	0.0434	2791.08545	1071.73022	95.6343
3	9.738	MF	0.0470	81.04501	28.72341	2.7769
4	9.925	MM	0.0431	15.71022	6.07605	0.5383
Totals :				2918.49907	1115.98187	

HPLC trace of compound **18c**

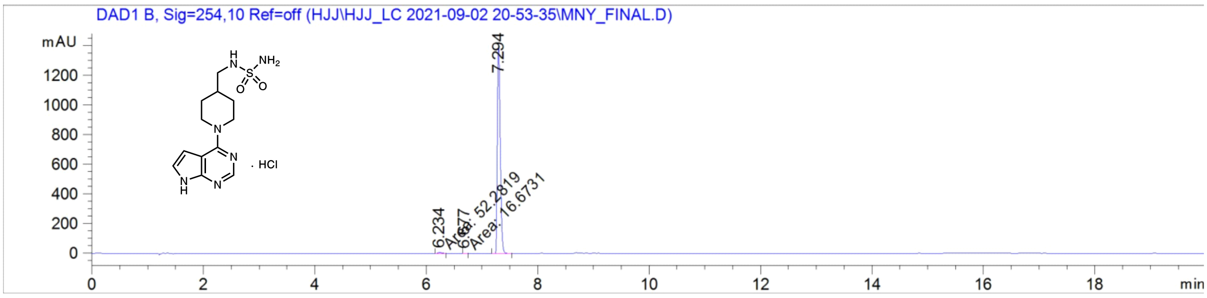

Peak #	RetTime [min]	Type	Width [min]	Area [mAU*s]	Height [mAU]	Area %
1	6.234	MM	0.1065	52.28189	8.18154	1.0028
2	6.677	MM	0.0809	16.67315	3.43693	0.3198
3	7.294	VV	0.0574	5144.58789	1415.72339	98.6774
Totals :				5213.54293	1427.34186	

HPLC trace of compound **18d**

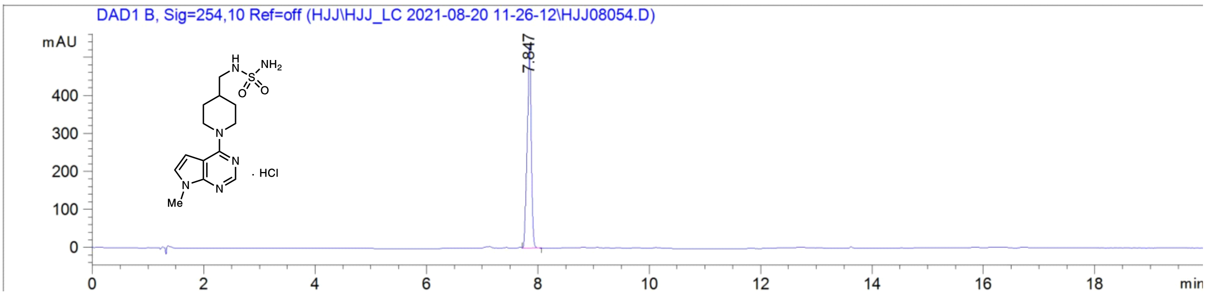

Peak #	RetTime [min]	Type	Width [min]	Area [mAU*s]	Height [mAU]	Area %
1	7.847	VB	0.0706	2695.10669	536.91132	100.0000
Totals :				2695.10669	536.91132	

HPLC trace of compound **18e**

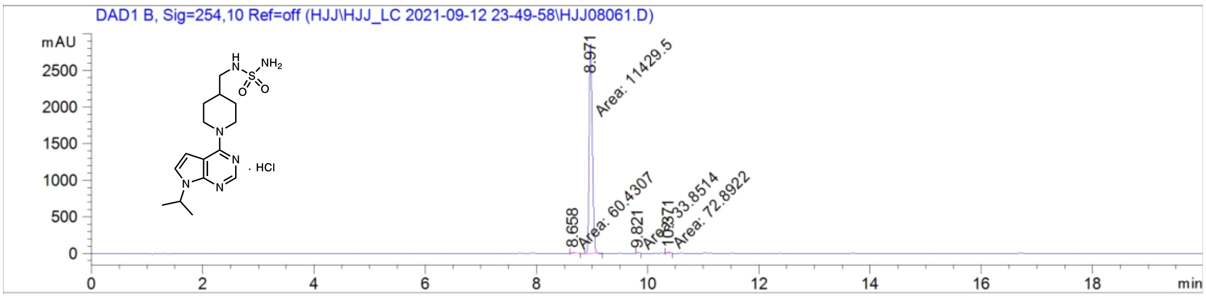

Peak #	RetTime [min]	Type	Width [min]	Area [mAU*s]	Height [mAU]	Area %
1	8.658	MM	0.0657	60.43071	15.33446	0.5211
2	8.971	MM	0.0667	1.14295e4	2857.55420	98.5584
3	9.821	MM	0.0504	33.85139	11.19858	0.2919
4	10.371	MM	0.0552	72.89222	22.00238	0.6286
Totals :				1.15966e4	2906.08962	

HPLC trace of compound **18f**

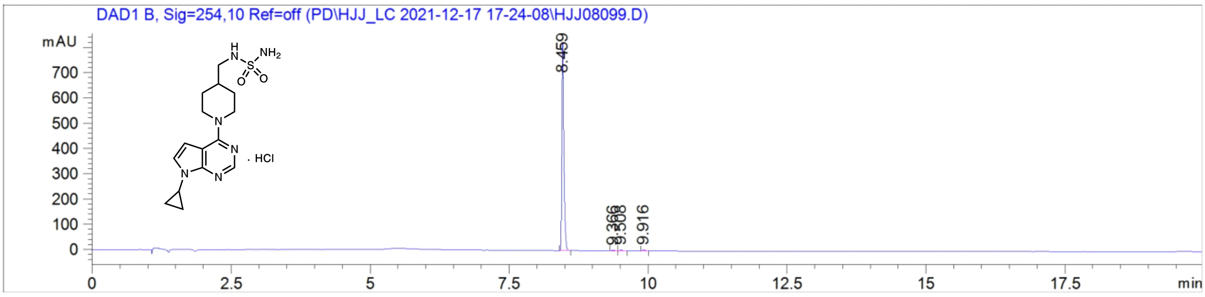

Peak #	RetTime [min]	Type	Width [min]	Area [mAU*s]	Height [mAU]	Area %
1	8.459	BV	0.0378	2088.69336	818.07056	97.9658
2	9.366	VV	0.0620	11.30666	2.69405	0.5303
3	9.508	VB	0.0431	14.73045	5.03612	0.6909
4	9.916	VV	0.0494	17.33250	5.12751	0.8129
Totals :				2132.06297	830.92823	

HPLC trace of compound **18g**

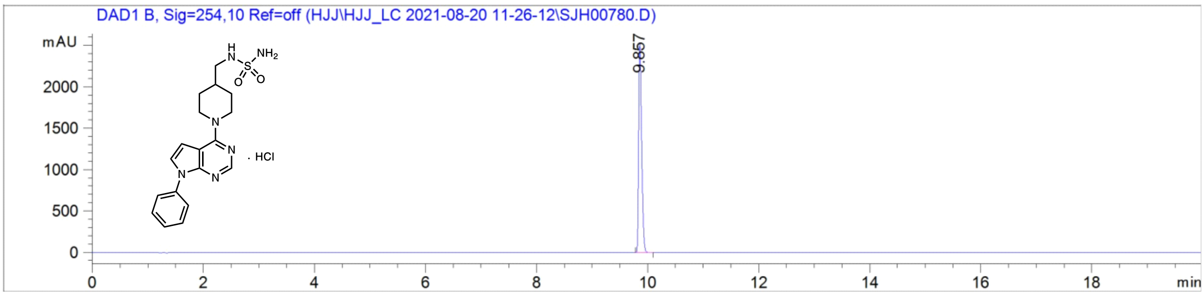

Peak #	RetTime [min]	Type	Width [min]	Area [mAU*s]	Height [mAU]	Area %
1	9.857	BB	0.0583	9557.58496	2517.96289	100.0000

Totals : 9557.58496 2517.96289

HPLC trace of compound **18h**

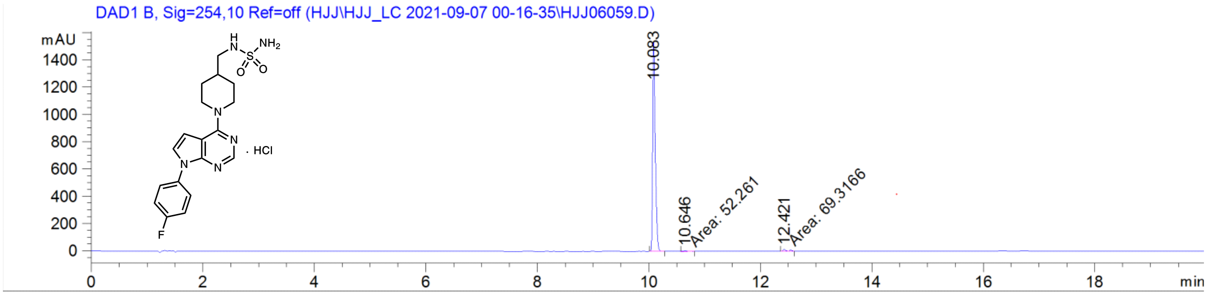

Peak #	RetTime [min]	Type	Width [min]	Area [mAU*s]	Height [mAU]	Area %
1	10.083	BV	0.0474	4917.79834	1535.82141	97.5874
2	10.646	MM	0.1000	52.26095	8.71239	1.0371
3	12.421	MM	0.0844	69.31664	13.69473	1.3755

Totals : 5039.37593 1558.22854

HPLC trace of compound **18i**

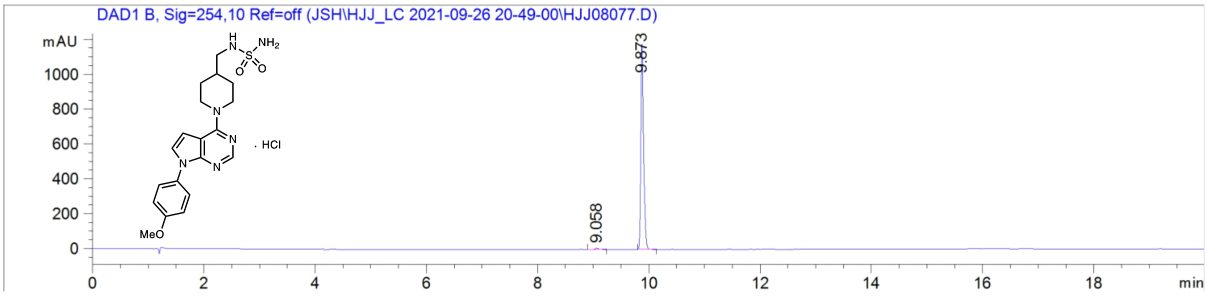

Peak #	RetTime [min]	Type	Width [min]	Area [mAU*s]	Height [mAU]	Area %
1	9.058	VB	0.0730	45.72509	8.60855	1.1074
2	9.873	BB	0.0504	4083.19653	1180.06738	98.8926

Totals : 4128.92163 1188.67593

HPLC trace of compound **18j**

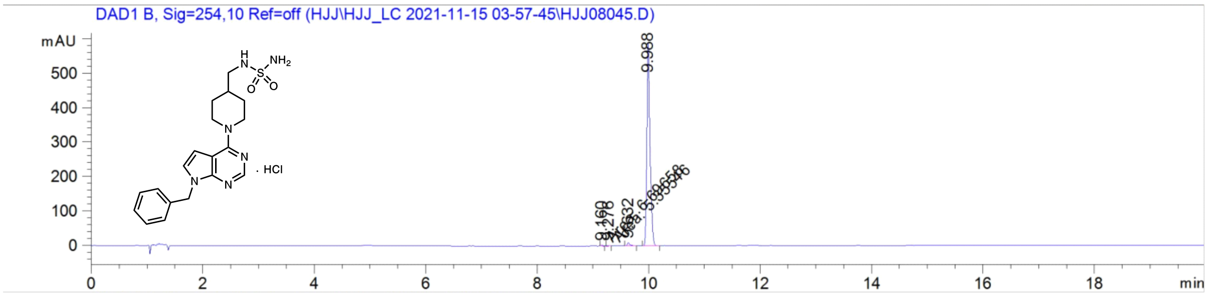

Peak #	RetTime [min]	Type	Width [min]	Area [mAU*s]	Height [mAU]	Area %
1	9.160	MM	0.0508	6.69658	2.19735	0.2842
2	9.276	MM	0.0609	5.35546	1.46450	0.2272
3	9.632	BB	0.0555	36.92263	9.47020	1.5667
4	9.988	VB	0.0568	2307.72217	587.80847	97.9219

Totals : 2356.69684 600.94052

HPLC trace of compound **18k**

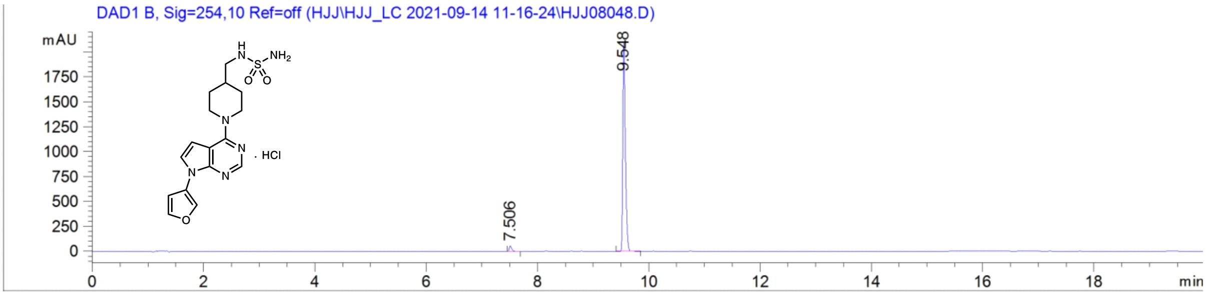

Peak #	RetTime [min]	Type	Width [min]	Area [mAU*s]	Height [mAU]	Area %
1	7.506	VB	0.0462	172.96170	55.75671	2.5211
2	9.548	VB	0.0481	6687.65137	2102.75513	97.4789

Totals : 6860.61307 2158.51184

HPLC trace of compound **18l**

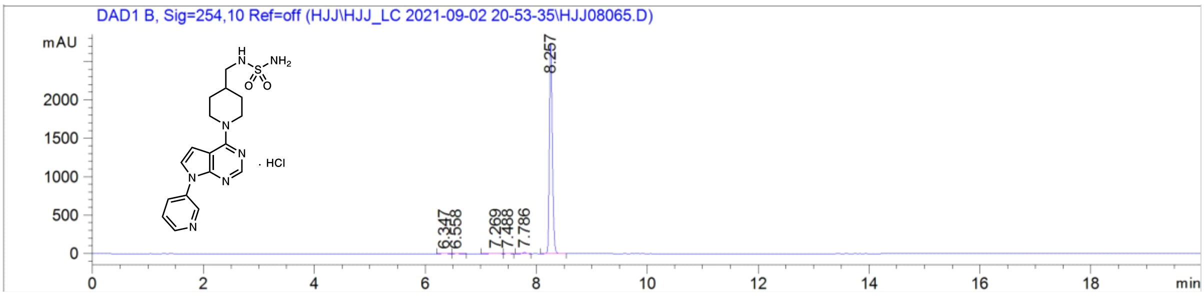

Peak #	RetTime [min]	Type	Width [min]	Area [mAU*s]	Height [mAU]	Area %
1	6.347	BV	0.1017	19.36068	2.79792	0.1869
2	6.558	VB	0.0794	12.70100	2.45332	0.1226
3	7.269	BV	0.0979	49.61424	6.66180	0.4789
4	7.488	VB	0.0515	24.72449	7.11786	0.2386
5	7.786	BV	0.0659	70.55387	15.26108	0.6810
6	8.257	VV	0.0566	1.01832e4	2723.61182	98.2920

Totals : 1.03601e4 2757.90380

HPLC trace of compound **18m**

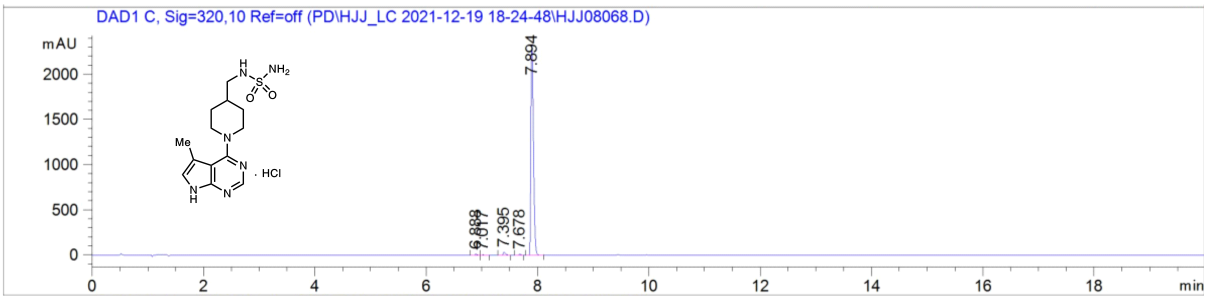

Peak #	RetTime [min]	Type	Width [min]	Area [mAU*s]	Height [mAU]	Area %
1	6.888	VV	0.0390	39.05124	14.67935	0.5015
2	7.017	VV	0.0444	12.92916	4.26678	0.1660
3	7.395	VV	0.0452	107.21523	34.59141	1.3769
4	7.678	VV	0.0377	34.58411	13.59994	0.4441
5	7.894	VV	0.0502	7593.19043	2321.17212	97.5115
Totals :				7786.97016	2388.30959	

HPLC trace of compound **18n**

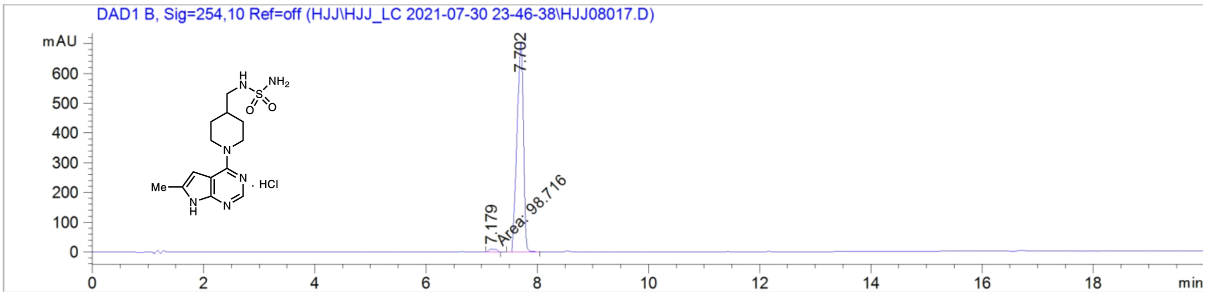

Peak #	RetTime [min]	Type	Width [min]	Area [mAU*s]	Height [mAU]	Area %
1	7.179	MM	0.1576	98.71600	10.43865	1.7013
2	7.702	BB	0.1169	5703.83057	701.47125	98.2987
Totals :				5802.54657	711.90991	

HPLC trace of compound **18o**

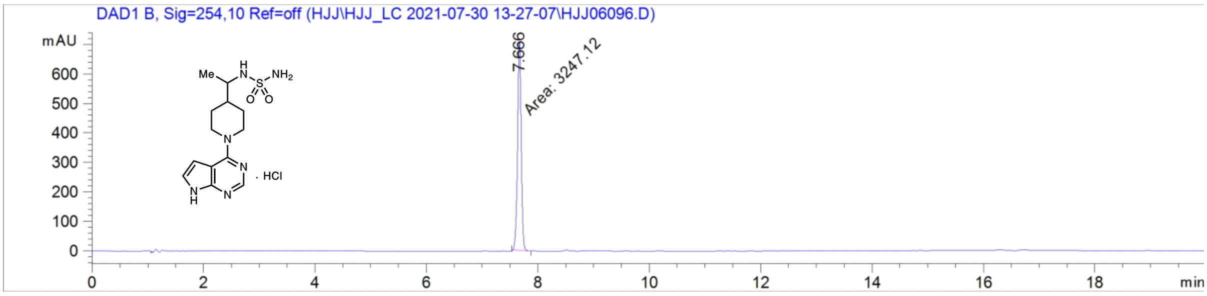

Peak #	RetTime [min]	Type	Width [min]	Area [mAU*s]	Height [mAU]	Area %
1	7.666	MM	0.0768	3247.12354	705.00452	100.0000
Totals :				3247.12354	705.00452	

HPLC trace of compound **18p**

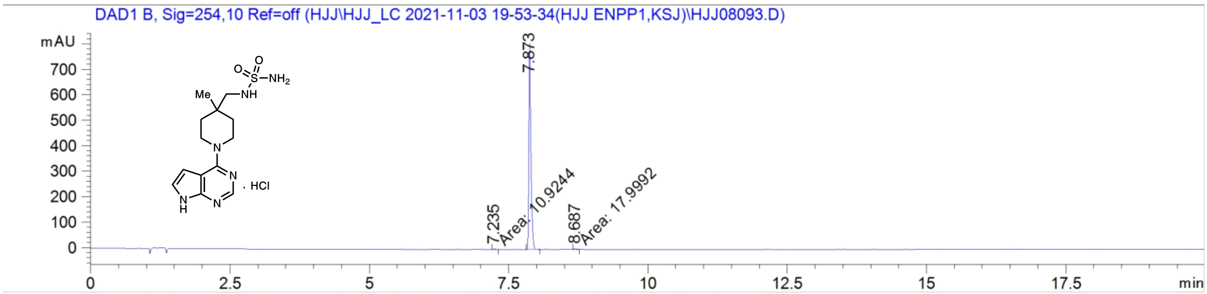

Peak #	RetTime [min]	Type	Width [min]	Area [mAU*s]	Height [mAU]	Area %
1	7.235	MM	0.0535	10.92444	3.40595	0.5146
2	7.873	BB	0.0383	2094.17065	807.23492	98.6377
3	8.687	MM	0.0630	17.99922	4.76079	0.8478
Totals :				2123.09432	815.40167	

HPLC trace of compound **18q**

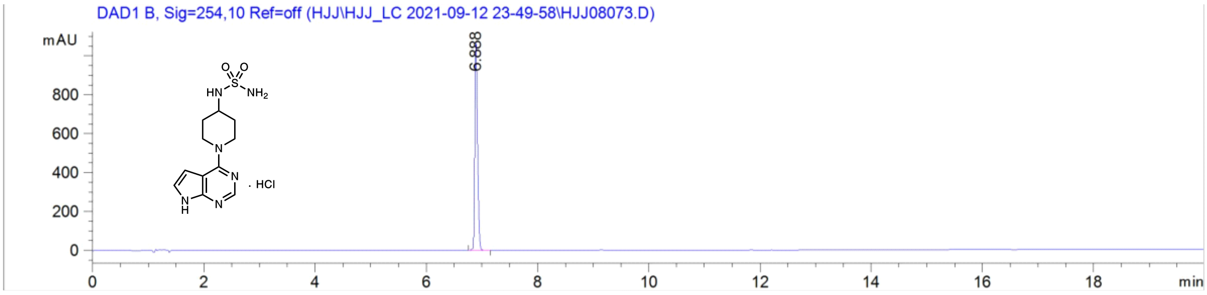

Peak #	RetTime [min]	Type	Width [min]	Area [mAU*s]	Height [mAU]	Area %
1	6.888	BB	0.0505	3623.36084	1070.19214	100.0000
Totals :				3623.36084	1070.19214	

HPLC trace of compound **18r**

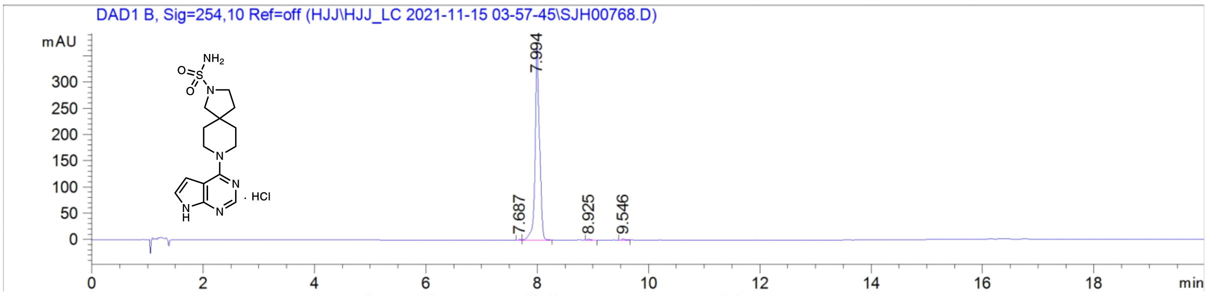

Peak #	RetTime [min]	Type	Width [min]	Area [mAU*s]	Height [mAU]	Area %
1	7.687	BV	0.0473	7.98002	2.43234	0.3812
2	7.994	VV	0.0760	2054.69849	374.78421	98.1503
3	8.925	BB	0.0619	12.84549	2.83061	0.6136
4	9.546	VB	0.0768	17.89736	3.07899	0.8549
Totals :				2093.42136	383.12615	

HPLC trace of compound **20a**

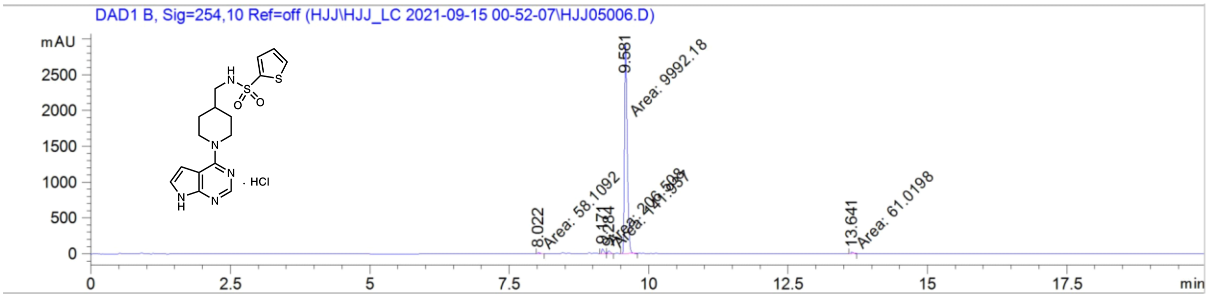

Peak #	RetTime [min]	Type	Width [min]	Area [mAU*s]	Height [mAU]	Area %
1	8.022	MM	0.0533	58.10921	18.18711	0.5556
2	9.171	MF	0.0510	206.50751	67.55150	1.9743
3	9.284	FM	0.0545	141.93692	43.40821	1.3570
4	9.581	MM	0.0568	9992.18164	2932.24121	95.5298
5	13.641	MM	0.0456	61.01983	22.29005	0.5834

Totals : 1.04598e4 3083.67809

HPLC trace of compound **20b**

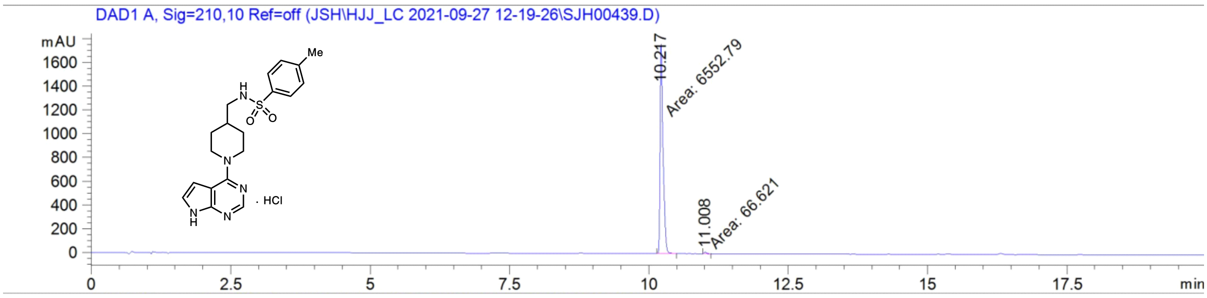

Peak #	RetTime [min]	Type	Width [min]	Area [mAU*s]	Height [mAU]	Area %
1	10.217	MM	0.0617	6552.79102	1770.19495	98.9936
2	11.008	MM	0.0696	66.62105	15.95793	1.0064

Totals : 6619.41206 1786.15287

HPLC trace of compound **20c**

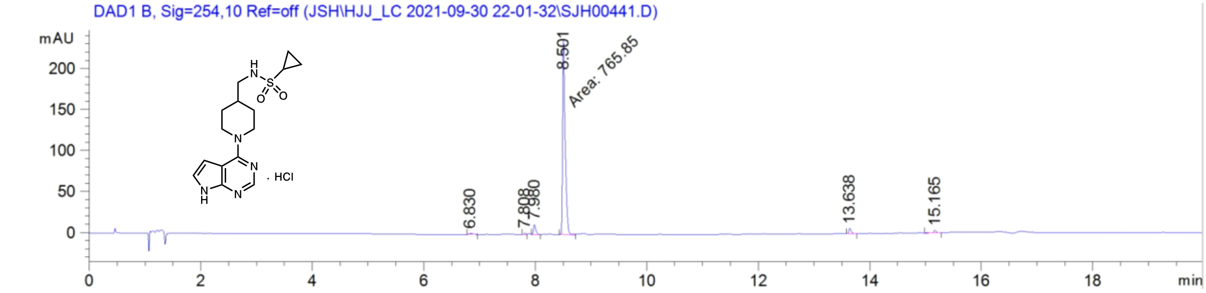

Peak #	RetTime [min]	Type	Width [min]	Area [mAU*s]	Height [mAU]	Area %
1	7.980	MM	0.0439	19.86834	7.54861	0.4995
2	8.501	MM	0.0532	3782.91772	1185.36658	95.1045
3	15.339	MF	0.0726	85.92370	19.71411	2.1602
4	16.266	MM	0.0568	88.93454	26.09015	2.2359

Totals : 3977.64430 1238.71945

HPLC trace of compound **25a**

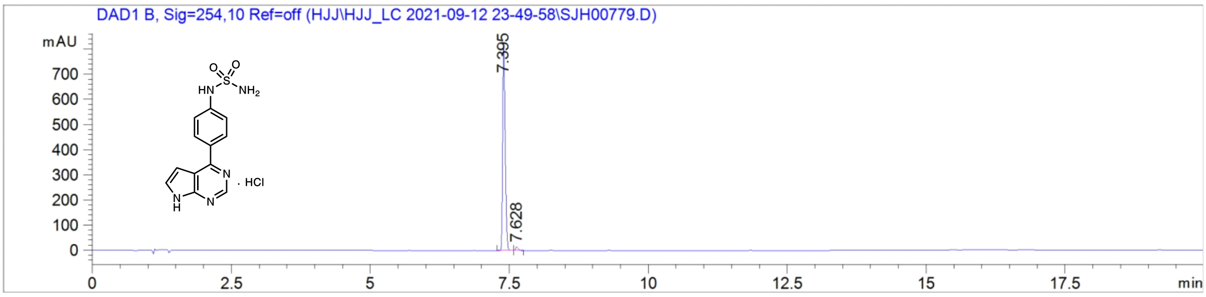

Peak #	RetTime [min]	Type	Width [min]	Area [mAU*s]	Height [mAU]	Area %
1	7.395	BV	0.0472	2622.58594	823.46271	98.0422
2	7.628	VB	0.0529	52.36898	14.57034	1.9578
Totals :				2674.95492	838.03305	

HPLC trace of compound **25b**

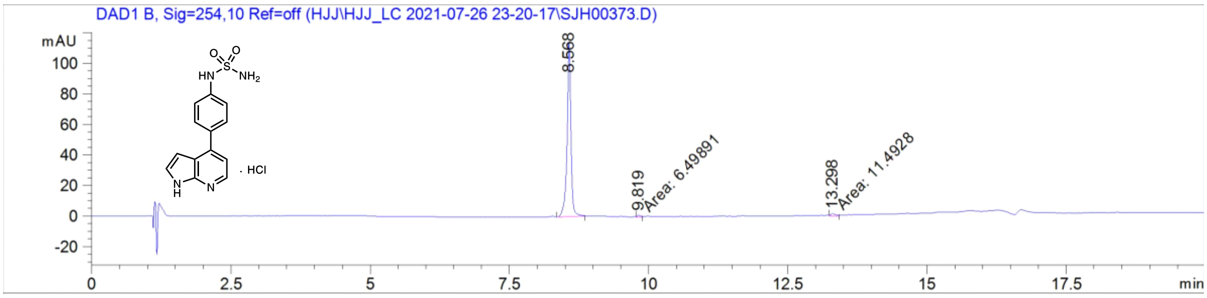

Peak #	RetTime [min]	Type	Width [min]	Area [mAU*s]	Height [mAU]	Area %
1	8.568	VB	0.0694	548.97760	113.56799	96.8267
2	9.819	MM	0.0743	6.49891	1.45752	1.1463
3	13.298	MM	0.1072	11.49285	1.78725	2.0271
Totals :				566.96936	116.81276	

HPLC trace of compound **25c**

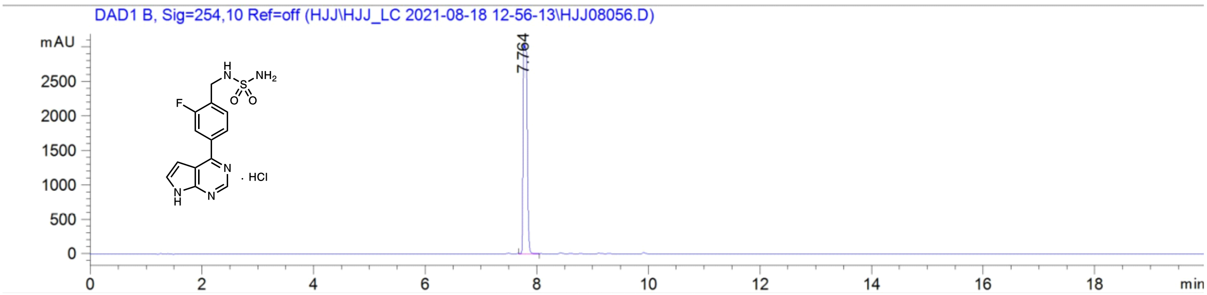

Peak #	RetTime [min]	Type	Width [min]	Area [mAU*s]	Height [mAU]	Area %
1	7.764	VV	0.0635	1.52988e4	3051.84375	100.0000
Totals :				1.52988e4	3051.84375	

HPLC trace of compound **25d**

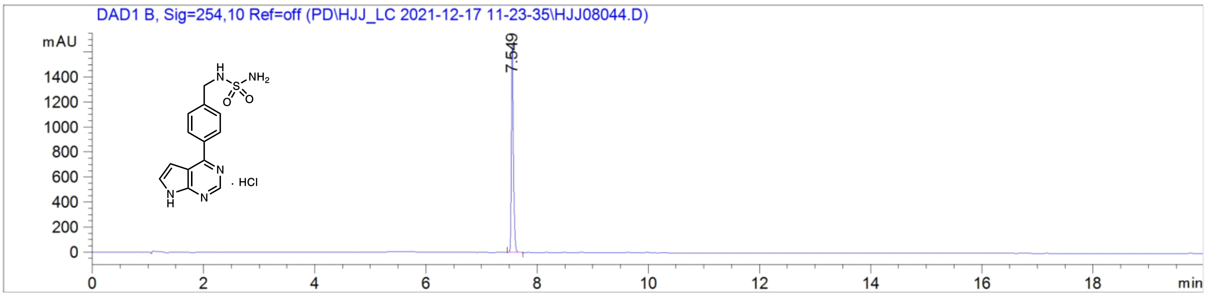

Peak #	RetTime [min]	Type	Width [min]	Area [mAU*s]	Height [mAU]	Area %
1	7.549	VB	0.0373	4188.78271	1667.50879	100.0000
Totals :				4188.78271	1667.50879	

HPLC trace of compound **25e**

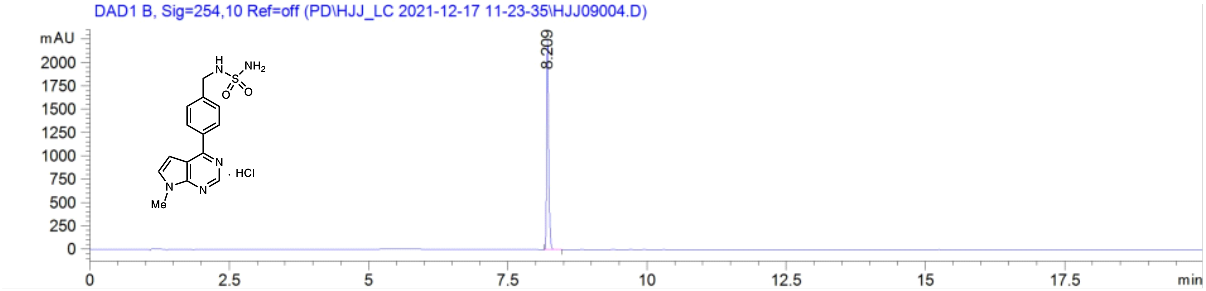

Peak #	RetTime [min]	Type	Width [min]	Area [mAU*s]	Height [mAU]	Area %
1	8.209	BB	0.0374	5647.41162	2243.00586	100.0000
Totals :				5647.41162	2243.00586	

HPLC trace of compound **25f**

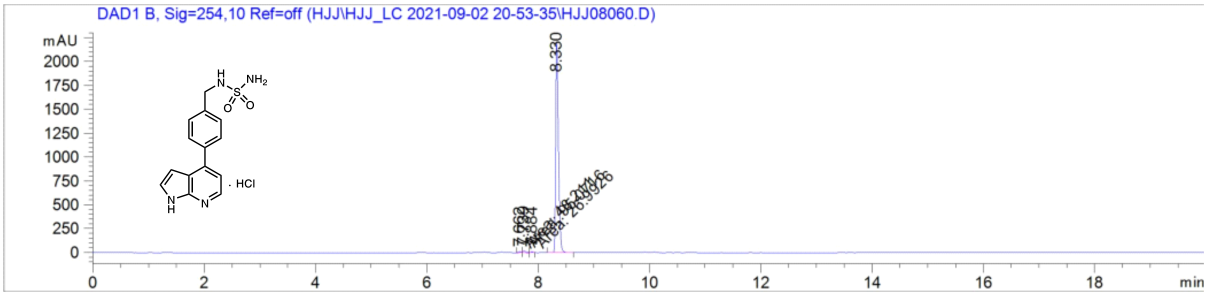

Peak #	RetTime [min]	Type	Width [min]	Area [mAU*s]	Height [mAU]	Area %
1	7.662	MM	0.0603	48.21103	13.31994	0.6153
2	7.739	MM	0.0681	95.07157	23.25786	1.2134
3	7.884	MM	0.0492	26.99259	9.14576	0.3445
4	8.330	VB	0.0517	7664.83789	2198.15088	97.8268
Totals :				7835.11308	2243.87444	

HPLC trace of compound **25g**

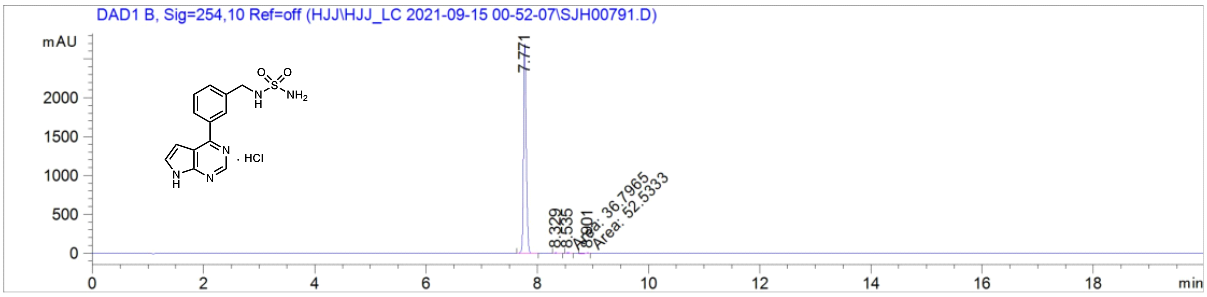

Peak #	RetTime [min]	Type	Width [min]	Area [mAU*s]	Height [mAU]	Area %
1	7.771	VB	0.0509	9210.51953	2696.41162	98.690[illegible]
2	8.329	VB	0.0555	32.86955	8.60794	0.352[illegible]
3	8.535	MM	0.0782	36.79649	7.84243	0.394[illegible]
4	8.901	MM	0.1369	52.53331	6.39456	0.562[illegible]
Totals :				9332.71888	2719.25655	

HPLC trace of compound **25h**

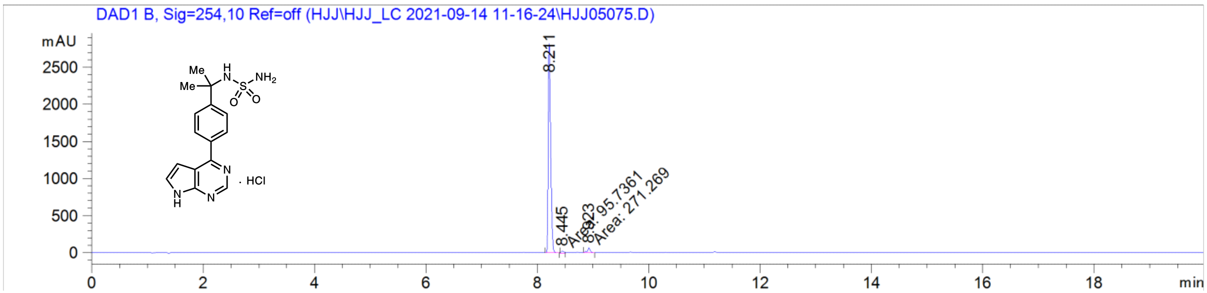

Peak #	RetTime [min]	Type	Width [min]	Area [mAU*s]	Height [mAU]	Area %
1	8.211	VV	0.0451	8501.78418	2822.78931	95.8618
2	8.445	MM	0.0551	95.73613	28.97942	1.0795
3	8.923	MM	0.0681	271.26917	66.41212	3.0587
Totals :				8868.78947	2918.18084	

HPLC trace of compound **25i**

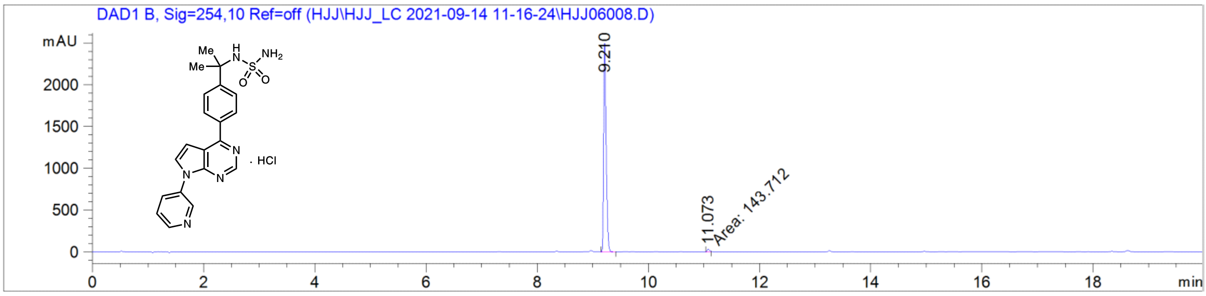

Peak #	RetTime [min]	Type	Width [min]	Area [mAU*s]	Height [mAU]	Area %
1	9.210	VV	0.0465	7806.18555	2498.31909	98.1923
2	11.073	MM	0.0588	143.71153	40.72609	1.8077
Totals :				7949.89708	2539.04518	

HPLC trace of compound **25j**

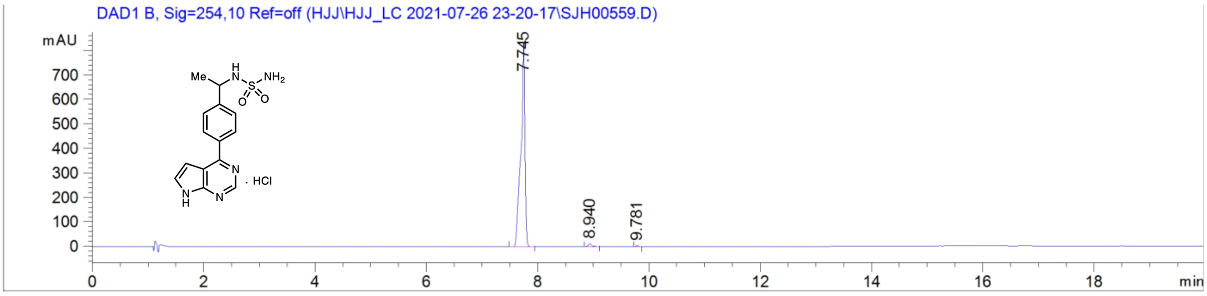

Peak #	RetTime [min]	Type	Width [min]	Area [mAU*s]	Height [mAU]	Area %
1	7.745	BB	0.0675	4179.05664	833.55334	98.3053
2	8.940	BB	0.0597	57.48204	13.50038	1.3522
3	9.781	BV	0.0472	14.56271	4.69951	0.3426

Totals : 4251.10139 851.75323
